# A Conserved MicroRNA Regulatory Circuit Is Differentially Controlled during Limb/Appendage Regeneration

**DOI:** 10.1371/journal.pone.0157106

**Published:** 2016-06-29

**Authors:** Benjamin L. King, Viravuth P. Yin

**Affiliations:** 1 Kathryn W. Davis Center for Regenerative Biology and Medicine, Mount Desert Island, Biological Laboratory, Salisbury Cove, Maine, United States of America; 2 Graduate School of Biomedical Science and Engineering, University of Maine, Orono, Maine, United States of America; Deakin School of Medicine, AUSTRALIA

## Abstract

**Background:**

Although regenerative capacity is evident throughout the animal kingdom, it is not equally distributed throughout evolution. For instance, complex limb/appendage regeneration is muted in mammals but enhanced in amphibians and teleosts. The defining characteristic of limb/appendage regenerative systems is the formation of a dedifferentiated tissue, termed blastema, which serves as the progenitor reservoir for regenerating tissues. In order to identify a genetic signature that accompanies blastema formation, we employ next-generation sequencing to identify shared, differentially regulated mRNAs and noncoding RNAs in three different, highly regenerative animal systems: zebrafish caudal fins, bichir pectoral fins and axolotl forelimbs.

**Results:**

These studies identified a core group of 5 microRNAs (miRNAs) that were commonly upregulated and 5 miRNAs that were commonly downregulated, as well as 4 novel tRNAs fragments with sequences conserved with humans. To understand the potential function of these miRNAs, we built a network of 1,550 commonly differentially expressed mRNAs that had functional relationships to 11 orthologous blastema-associated genes. As miR-21 was the most highly upregulated and most highly expressed miRNA in all three models, we validated the expression of known target genes, including the tumor suppressor, *pdcd4*, and TGFβ receptor subunit, *tgfbr2* and novel putative target genes such as the anti-apoptotic factor, *bcl2l13*, Choline kinase alpha, *chka* and the regulator of G-protein signaling, *rgs5*.

**Conclusions:**

Our extensive analysis of RNA-seq transcriptome profiling studies in three regenerative animal models, that diverged in evolution ~420 million years ago, reveals a common miRNA-regulated genetic network of blastema genes. These comparative studies extend our current understanding of limb/appendage regeneration by identifying previously unassociated blastema genes and the extensive regulation by miRNAs, which could serve as a foundation for future functional studies to examine the process of natural cellular reprogramming in an injury context.

## Background

The capacity to regenerate damaged and amputated limb/appendage tissues is a trait that is unevenly distributed throughout the animal kingdom [1–3]. Mammals, including humans, have limited regenerative capacity of limb tissues, able to only replace distal digit tips [4–6]. On the contrary, adult ray-finned fish and urodele amphibians can regenerate fully-functional appendages following amputation, replacing bone, muscle, connective tissue, epidermis, nerves, blood vessels and pigment cells [3,7]. How and why certain organisms are better equipped to replace missing or damaged tissues has perplexed biologists for over three centuries. We postulate that a shared, conserved genetic regulatory circuit coordinates regeneration and patterning of limb/appendage tissues in animals that are separated by millions of years in evolution.

The defining feature of animals endowed with limb/appendage regenerative capacity is the formation of a dedifferentiated, highly proliferative tissue termed, blastema. This regenerative tissue arises through dedifferentiation of spared cells located proximal to an amputation plane [8]. Genetic defects in blastema formation results in abrogation of limb/appendage regeneration [9,10]. Yet, despite its importance, the genetic determinants required for blastema formation and maintenance has been understudied. While previous mRNA profiling studies from zebrafish and axolotl provide insight into the mechanisms of the individual models [11–13], there are no studies to date that directly compare gene expression changes across models at equivalent stages of regeneration. The Bichir has recently emerged as a model system for appendage regeneration [14], however, a comprehensive transcriptome analysis focused on blastema formation has not been conducted.

The transition from differentiated tissue into highly proliferative cells is underscored by dramatic shifts in expression of developmental genetic programs. This rapid transformation involves regulation at multiple levels, including at the posttranscriptional level by miRNAs [15–19]. MiRNAs are short, highly conserved noncoding RNAs that inhibit gene expression through complementary base-pairing with the 3’ untranslated region (UTR) of target mRNAs, culminating in destabilization and/or degradation of bound mRNAs [20–22]. MiRNAs are ideal candidates for comparative studies of genetic regulation as they are highly conserved during evolution and can regulate large sets of critical target genes for tissue regeneration [23–26]. Recently, zebrafish appendage regeneration studies have revealed two differentially regulated miRNAs, miR-133 [27] and miR-203 [28], as essential regulators of caudal fin regeneration. Whether these and other miRNAs are controlled in an analogous manner in the axolotl and bichir remains an open question.

In this study, we performed transcriptome analysis of both miRNA and mRNA expression during blastema formation in three systems, *Danio rerio* (zebrafish) caudal fins, *Polypterus senegalus* (bichir) pectoral fins and *Ambystoma mexicanum* (axolotl) forelimbs. These studies identified a core group of 5 miRNAs that were commonly upregulated and 5 miRNAs that were commonly downregulated. To understand the potential function of these miRNAs, we built a network of 1,550 commonly differentially expressed mRNAs that had functional relationships to 11 orthologous blastema-associated genes. Next, we established a gene network for common miRNA target genes for miR-21, miR-31 and miR-181. As miR-21 was the most highly upregulated and most highly expressed miRNA in all three models, we validated the expression of known target genes, including the tumor suppressor, *pdcd4*, and TGFβ receptor subunit, *tgfbr2* [29,30] and novel putative targets such as the anti-apoptotic factor, *bcl2l13*, the Choline Kinase Alpha, *chka* and the Regulator of G-protein signaling, *rgs5*. Together, our study demonstrates the high value of framing studies in a comparative biological context and to the best of our knowledge, this cross-species comparative study revealed for the first time, a conserved miRNA and mRNA genetic network for blastema formation during limb/appendage regeneration.

## Results

### Blastema formation in zebrafish, bichir and axolotl

**Fig 1:**
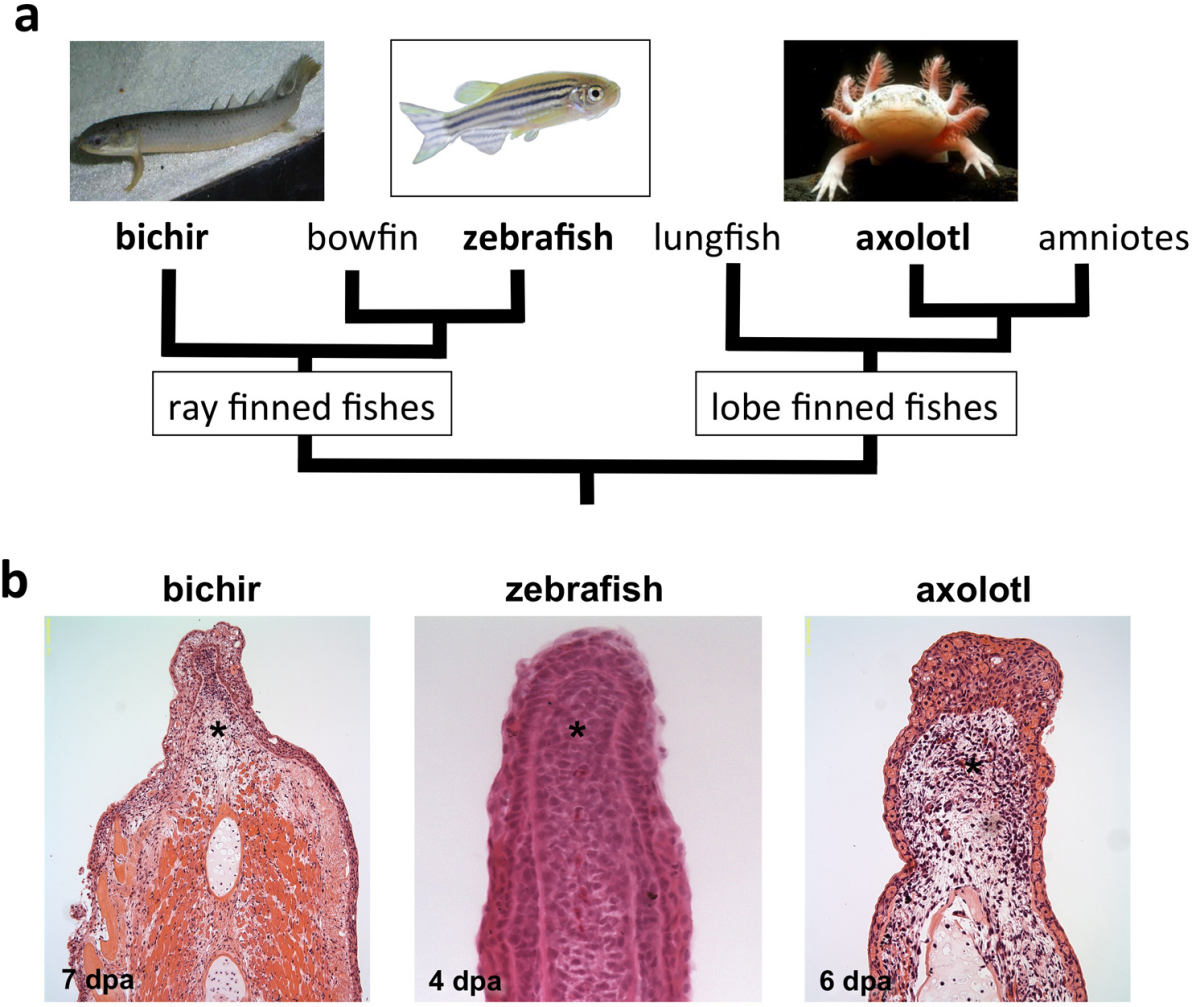
Vertebrate models used to study limb/appendage regeneration. **(a)** Phylogenetic relationship among three vertebrate taxa studied to determine conserved gene expression patterns in regenerating limb/appendages following blastema formation. These animal systems last shared a common ancestor ~420 million years ago. **(b)** Blastema tissues as shown by hematoxylin and eosin staining on paraffin tissue sections of regenerating zebrafish caudal fin, bichir pectoral fins and axolotl forelimbs. (* = blastema; dpa = days post-amputation).

Appendage regeneration is driven by the formation of a dedifferentiated and proliferative tissue called a blastema. To better understand if a regulatory unit has been conserved throughout evolution that controls blastema formation, we performed a comparative genomic study that includes three highly regenerative model systems, zebrafish, bichir and axolotl, animals that last shared a common ancestor ~420 million years ago [31,32]. Bichir and zebrafish represent the ray-finned clade (actinopterygian) and axolotl belongs to the lobe-finned (sarcopterygian) lineage (Fig 1A). The zebrafish is a widely utilized biomedical model organism with a sequenced genome, an increasing number of genetic tools for functional studies, and an expanding genetic and cellular understanding for appendage regeneration. Bichir belongs to the lineage of the most basal living ray-finned fish, polypteriforms [31], and was selected to represent a ray-finned fish lineage that is distinct from zebrafish and other teleosts. The axolotl, an amphibian that has been widely studied for its regenerative capacity, was chosen to represent the lobe-finned fish lineage.

Although all zebrafish appendages are capable of regeneration, we selected the caudal fin for our studies due to the defined timing of blastema formation [33,34]. By 4 days post-amputation (dpa), the caudal fin blastema is established, as revealed by hematoxylin and eosin staining on paraffin tissue sections (Fig 1B). Amputated axolotl forelimbs and bichir pectoral fins form blastemas by 6 and 7 dpa, respectively (Fig 1B) [14,35,36]. To initiate our studies of shared genetic regulatory units for these model systems, we amputated 50% of the zebrafish caudal and bichir pectoral fins, and induced an amputation mid-zeugopod (mid-radius) of the axolotl forelimb. We extracted tissue from uninjured and regenerating samples at 4 dpa for zebrafish, 7 dpa for bichir and 6 dpa for axolotl to coincide with blastema formation and proliferation. Total RNA was isolated for transcriptome analysis of mRNAs and noncoding small RNAs using the Illumina high-throughput sequencing platform. We performed transcriptome analysis of the zebrafish genome in triplicate, with each replicate consisting of 5–8 fin samples for each stage of regeneration. Bichir and axolotl experiments were conducted with a single replicate consisting of at least 4-pooled tissues for each stage.

### Characterization of miRNAs expressed in regenerating tissues

Mature miRNA sequences are highly conserved throughout evolution and have documented roles during tissue repair and regeneration [37–40], making them ideal candidate factors to stimulate blastema formation. Our comparative transcriptome profiling studies identified previously annotated and novel miRNAs for the three taxa. We found a total of 221 known miRNAs in zebrafish, 154 in bichir and 143 in axolotl (S1 File). Although zebrafish miRNAs have been examined in numerous studies [25,27,41–43], our analysis revealed novel paralogs of 18 miRNAs that do not currently have zebrafish records in miRBase (version 21), including miR-181a, miR-20a, miR-23b, miR-24, miR-29a, miR-103, miR-128, miR-148, miR-181b, miR-199, miR-204, miR-212, miR-221, miR-338, miR-724, miR-2184, let-7b and let-7e. These additional paralogs are mapped to distinct genomic loci that produce the same mature miRNA product for a given miRNA. Moreover, we identified new miRNA family members for miR-551, miR-7132 and miR-7133. Four miRNAs in miRBase, miR-722, miR-735 and miR-740 and miR-7146, were excluded from the analysis as their mapped genomic locations conflicted with miRBase annotation.

**Fig 2:**
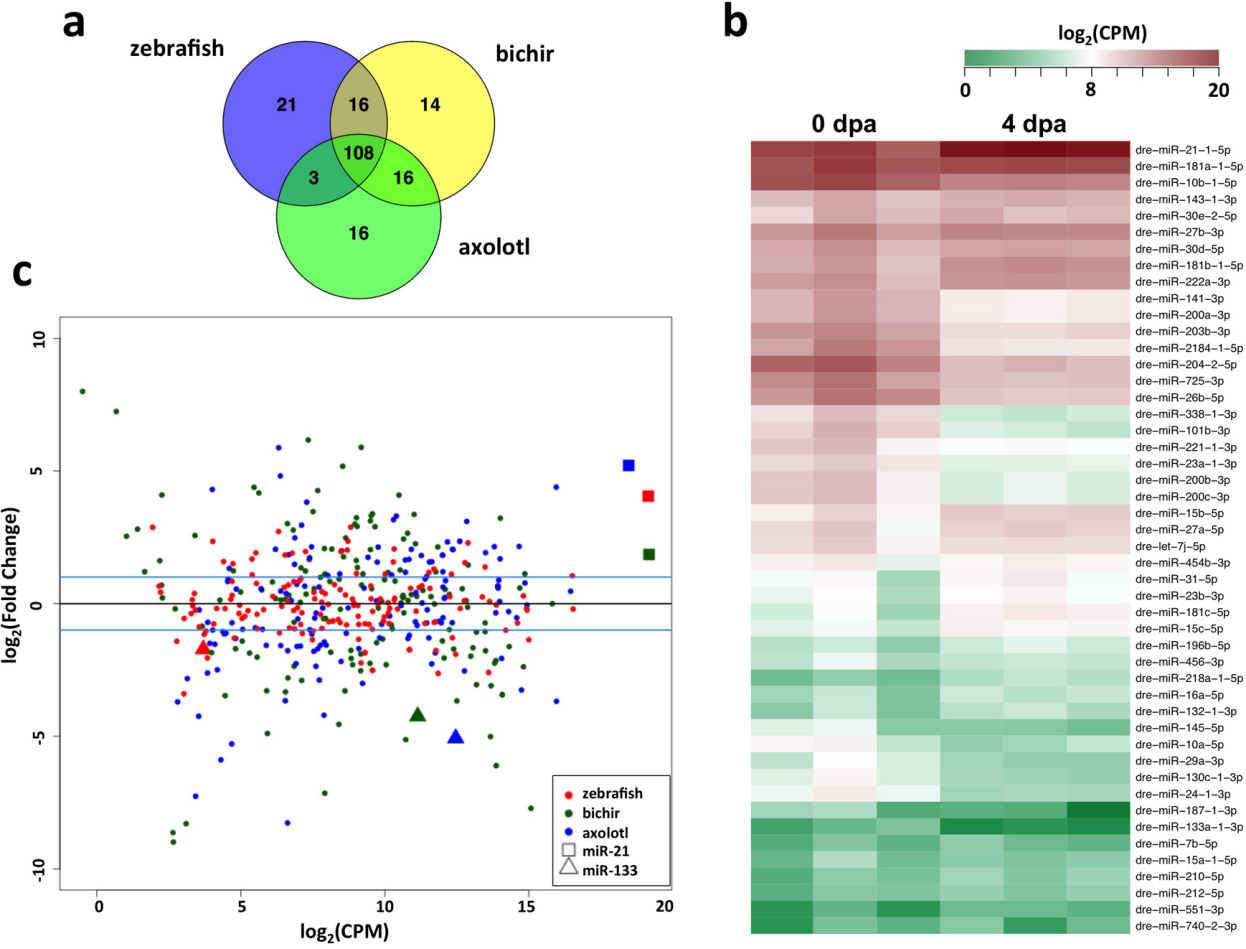
miRNA expression profiling by small RNA sequencing. **(a)** Overview of shared and unique miRNAs detected in regenerating limb/appendages following blastema formation in zebrafish caudal fin, bichir pectoral fins and axolotl forelimbs. **(b)** Gene expression patterns of differentially expressed zebrafish miRNAs shown as a heat map of log_2_-transformed read counts per million, in triplicate. **(c)** Relationship among fold-change and average level of expression for each miRNA in each taxa with miR-21 and miR-133. (dpa = days post-amputation).

Since miRNAs in bichir and axolotl are not yet represented in miRBase [44], we annotated known and putative novel miRNAs using miRMiner [45]. The annotation workflow for miRNAs is best done by aligning reads to a genome assembly, looking for characteristic stem-loop precursor structures and examining the pattern of sequence tag alignments [46]. As bichir and axolotl don’t have genome assemblies, the miRMiner software was used to compare adapter clipped and collapsed small RNA sequence reads from bichir and axolotl to mature sequences in miRBase to identify miRNAs. These analyses revealed 154 miRNAs in bichir and 143 in axolotl (S1 File), of which, 108 mature miRNA products were shared by all three systems, 16 unique miRNAs were shared between zebrafish and bichir, and 3 unique miRNAs were shared between zebrafish and axolotl (Fig 2A and S1 Table). As expected, since zebrafish and bichir are both ray-finned fishes, they had more shared miRNAs than comparisons to axolotl.

### Differential miRNA expression during blastema formation

**Fig 3:**
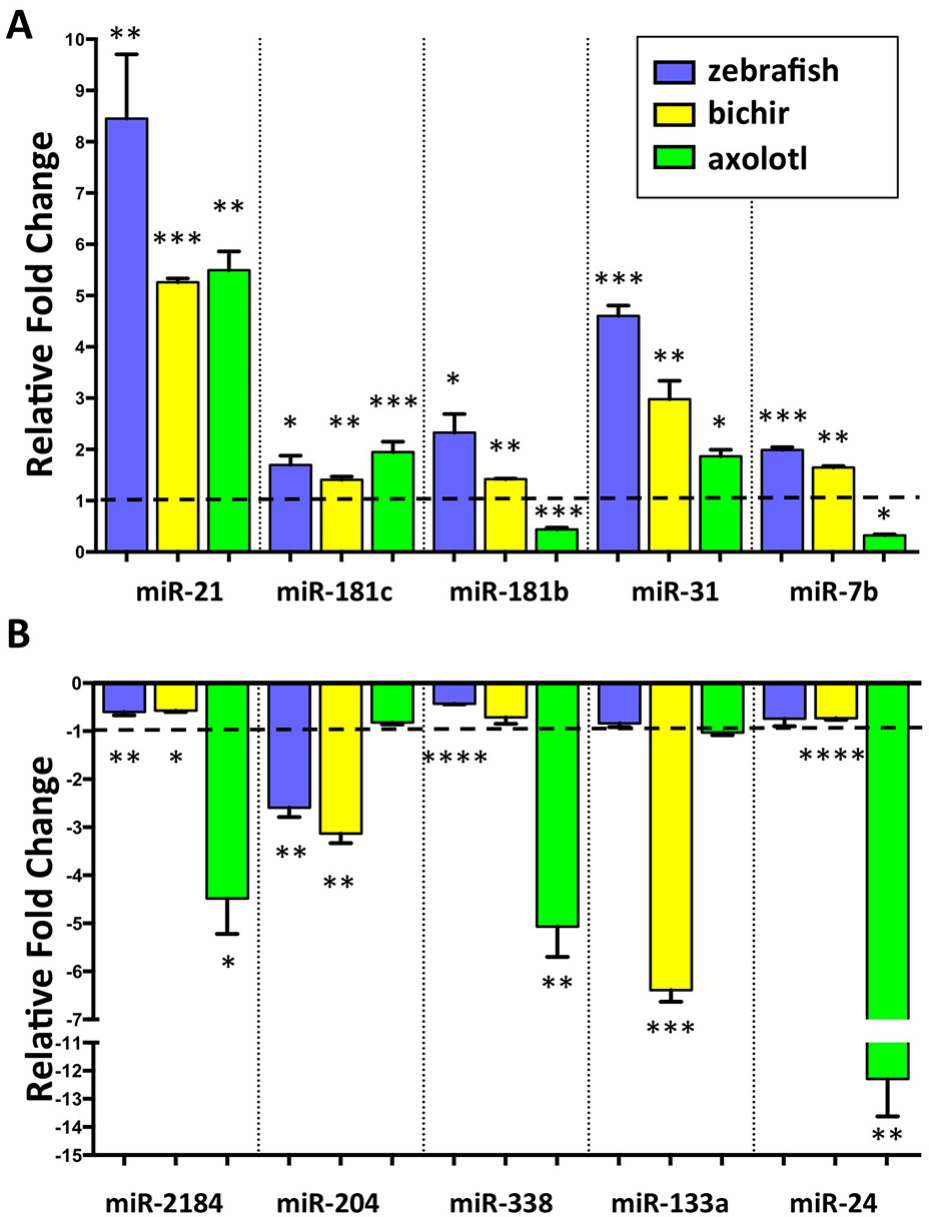
Differentially regulated miRNAs shared among zebrafish, bichir and axolotl. **(a, b)** qRT-PCR validation of differential expression of commonly upregulated and downregulated miRNAs in regenerating zebrafish caudal fins (0 dpa vs. 4 dpa), bichir pectoral fins (0 dpa vs. 7 dpa) and axolotl forelimbs (0 dpa vs. 6 dpa). At least 4 regenerating tissues per replicate were used, in triplicate. Average values +/- SEM are plotted. (*, **, *** = p-value < 0.01, 0.005 and 0.001 respectively).

To identify miRNAs that are potentially important for blastema formation, we quantified sequence tag counts for each annotated miRNA for the three organisms. The zebrafish has a sequenced and well-annotated genome, and thus, was used as a foundation to identify shared differentially expressed miRNAs in response to limb/appendage injury. From the 221 miRNAs identified from zebrafish, 27 were significantly upregulated and 21 were downregulated between uninjured and 4 dpa regenerating caudal fins (Fig 2B and S2 Table). Within this subset of differentially regulated zebrafish miRNAs, we identified 10 miRNAs: miR-21, miR-181c, miR-181b, miR-31, miR-7b, miR-2184, miR-24, miR-133a, miR-338 and miR-204, that showed conserved expression changes with both bichir and axolotl regenerating samples (Table 1). miR-21 was the most abundantly expressed miRNA and the most highly upregulated in all three systems (Fig 2C and S2–S4 Tables). In addition, our analysis identified 12 differentially regulated miRNAs shared between zebrafish and bichir and 2 miRNAs were shared between zebrafish and axolotl. A comparison between regenerating bichir and axolotl samples revealed 42 miRNAs that were commonly up- or downregulated (Table 2 and S1 Fig). To validate expression changes of shared, differentially expressed miRNAs across all three systems, we employed real-time qPCR studies. These studies confirmed miR-21, miR-181c and miR-31 were consistently upregulated in all three organisms and miR-181b and miR-7b were upregulated in both zebrafish and bichir (Fig 3). Conversely, miR-204 was downregulated in zebrafish and bichir, miR-133a was downregulated in bichir, and miR-2184, miR-338 and miR-24 were downregulated in axolotl.

**Table 1:** Expression of miRNAs during blastema formation in zebrafish caudal fin, bichir pectoral fins and axolotl forelimbs.

**Zebrafish + Bichir + Axolotl**				**Zebrafish + Bichir**		
**Symbol**	**Zebrafish log**_**2**_ **Fold-change (p-value)**	**Bichir log**_**2**_ **Fold-change**	**Axolotl log**_**2**_ **Fold-change**	**Symbol**	**zebrafish log**_**2**_ **Fold-change (p-value)**	**Bichir log**_**2**_ **Fold-change**
miR-21	+4.05 (3.28e-8)	+1.85	+5.21	miR-218a	+2.72 (9.75e-6)	+1.23
miR-181c	+2.88 (2.41e-6)	+2.43	+2.13	miR-15b	+2.06 (5.92e-4)	+1.62
miR-181b	+2.29 (2.52e-4)	+1.17	+1.64	miR-210	+1.91 (1.91e-3)	+1.34
miR-31	+2.02 (6.14e-4)	+5.18	+2.96	miR-16a	+1.90 (1.16e-3)	+0.99
miR-7b	+1.50 (2.0e-2)	+4.26	+2.14	miR-132	+1.83 (1.71e-3)	+0.52
miR-2184	-2.63 (2.54e-5)	-2.25	-2.50	miR-222a	+1.54 (1.13e-2)	+3.24
miR-24	-1.36 (1.9e-2)	-1.41	-0.73	miR-454b	+1.14 (4.93e-2)	+0.14
miR-133a	-1.72 (2.67e-3)	-4.25	-5.07	miR-101b	-2.52 (3.44e-5)	-3.43
miR-338	-2.23 (1.90e-4)	-2.90	-1.57	miR-26b	-1.91 (1.84e-3)	-3.67
miR-204	-2.60 (4.76e-5)	-0.57	-2.36	miR-203b	-1.77 (3.45e3	-0.21
				miR-10b	-1.36 (2.90e-2)	-1.78
				miR-725	-1.29 (3.23e-2)	-1.62
**Zebrafish + Axolotl**				**Zebrafish**		
Symbol	Zebrafish log_2_ Fold-change (p-value)	Axolotl log_2_ Fold-change		Symbol	Zebrafish log_2_ Fold-change (p-value)	
miR-27a	+1.57 (7.96e-3)	+2.15		miR-27b	+1.38 (2.44e-2)	
miR-29b	-2.05 (1.28e-2)	-0.97		miR-143	+1.31 (2.89e-2)	
				miR-30e	+1.18 (4.80e-2)	
				miR-200c	-1.85 (1.72e-3)	
				miR-200a	-1.74 (3.66e-3)	
				miR-23a	-1.35 (2.05e-2)	

**Table 2:** Expression of miRNAs following blastema formation in regenerating bichir pectoral fins and axolotl forelimbs with log_2_(fold change) greater than +0.58 or less than -0.58 sorted by descending fold change. The first set of miRNAs were those that were commonly up- or down-regulated in bichir and axolotl. The second and third sets of miRNAs were those that were up- or down-regulated in bichir or axolotl, but not commonly up- or down-regulated.

Bichir + Axolotl			Bichir		Axolotl	
Symbol	Bichir log(Fold Change)	Axolotl log(Fold Change)	Symbol	Bichir log(Fold Change)	Symbol	Axolotl log(Fold Change)
miR-451	3.36	5.88	miR-16c	8.00	miR-203b	4.81
miR-21	1.85	5.21	miR-727	7.24	miR-203c	4.30
miR-203a	1.12	4.39	miR-214	4.38	miR-2970	3.82
miR-223	4.09	3.30	miR-456	4.17	miR-375	3.10
miR-142a	2.05	3.16	miR-430c	4.09	miR-144	2.48
miR-31	5.18	2.96	miR-193a	3.47	miR-182	2.35
miR-7b	4.26	2.14	miR-130b	3.31	miR-183	2.33
miR-181c	2.43	2.13	miR-222a	3.24	miR-27a	2.15
let-7b	2.91	2.04	miR-205	3.22	miR-25	2.15
miR-200	1.45	2.04	let-7j	3.12	miR-425	2.05
miR-155	3.02	1.99	let-7g	3.00	miR-200c	1.92
miR-92a	1.20	1.82	miR-365	2.90	miR-1329	1.84
miR-196a	1.77	1.67	miR-1306	2.81	miR-725	1.83
miR-193b	3.38	1.67	miR-19c	2.77	miR-10a	1.81
miR-181b	1.17	1.64	miR-150	2.65	miR-191	1.74
miR-221	5.89	1.56	miR-147	2.57	miR-181a	1.69
miR-146a	0.59	1.20	miR-875	2.54	miR-27d	1.58
miR-34a	2.08	1.06	miR-99	2.47	miR-30a	1.57
miR-1788	0.69	0.93	let-7h	2.26	miR-222b	1.48
miR-2188	6.17	0.83	miR-30c	2.13	miR-1662	1.32
miR-19a	0.83	0.66	miR-29a	2.06	let-7e	1.18
miR-26a	-2.05	-0.67	miR-457a	2.04	miR-96	1.12
miR-33a	-1.87	-0.71	miR-731	1.73	miR-20a	1.08
miR-27b	-2.07	-0.72	miR-15b	1.62	miR-196d	1.02
miR-24	-1.41	-0.73	miR-302	1.62	miR-429a	0.93
miR-34c	-1.77	-0.97	miR-125a	1.36	let-7f	0.89
miR-460	-4.90	-0.98	miR-210	1.35	miR-100	0.86
miR-458	-7.15	-1.06	miR-30b	1.27	miR-93	0.78
miR-106a	-1.41	-1.14	miR-218a	1.23	miR-9	0.73
miR-192	-2.30	-1.15	miR-139	1.23	miR-101b	0.66
miR-16b	-0.98	-1.20	miR-211	1.20	miR-454a	0.63
let-7c	-2.30	-1.31	miR-22a	1.12	miR-26b	0.62
miR-217	-3.33	-1.31	miR-92b	1.11	miR-30d	0.59
miR-338	-2.90	-1.57	miR-16a	0.99	miR-130b	-0.66
miR-218b	-1.85	-1.58	miR-29b	0.92	miR-19d	-0.68
miR-145	-1.90	-1.70	miR-17a	0.90	miR-106b	-0.72
miR-2184	-2.25	-2.50	miR-19b	0.85	miR-205	-0.73
miR-206	-6.11	-3.26	miR-15a	0.81	miR-17a	-0.78
miR-140	-3.06	-3.68	miR-135a	0.77	miR-125b	-0.83
miR-499	-4.55	-4.21	miR-20b	0.70	miR-19c	-0.91
miR-133a	-4.25	-5.07	miR-208	-0.70	miR-29b	-0.97
miR-152	-2.53	-8.27	let-7a	-0.73	miR-137	-1.00
			miR-27a	-0.81	miR-15a	-1.04
			miR-7133	-0.85	miR-132	-1.12
			miR-144	-0.96	miR-130a	-1.12
			miR-181a	-1.06	miR-30b	-1.29
			miR-30a	-1.07	let-7j	-1.29
			miR-100	-1.34	miR-455	-1.45
			miR-222b	-1.47	miR-208b	-1.50
			miR-725	-1.62	miR-281	-1.53
			let-7d	-1.67	miR-125a	-1.57
			miR-138	-1.70	miR-99	-1.57
			miR-27d	-1.72	miR-222a	-1.60
			miR-10b	-1.78	miR-456	-1.61
			miR-737	-1.84	miR-16a	-1.70
			let-7f	-1.98	miR-218a	-1.78
			miR-27e	-2.02	miR-454b	-1.80
			miR-30d	-2.15	miR-30c	-1.85
			miR-148	-2.25	miR-130c	-2.07
			miR-24b	-2.36	miR-15b	-2.18
			miR-30e	-2.38	miR-139	-2.26
			miR-9	-2.80	miR-199	-2.27
			let-7e	-3.10	miR-204	-2.36
			miR-730	-3.29	miR-1a	-2.54
			miR-10a	-3.29	let-7i	-2.62
			miR-101b	-3.43	miR-194a	-2.83
			miR-454a	-3.47	miR-214	-2.88
			miR-26b	-3.67	miR-301a	-3.01
			miR-143	-5.01	miR-128	-3.66
			miR-126a	-5.13	miR-124	-3.71
			miR-1b	-7.72	miR-146b	-4.25
			miR-2187	-8.30	miR-328	-5.29
			miR-153b	-8.63	let-7h	-5.89
			miR-315	-8.99	miR-135	-7.26
			miR-736	-10.91		
			miR-190a	-11.27		

**Fig 4:**
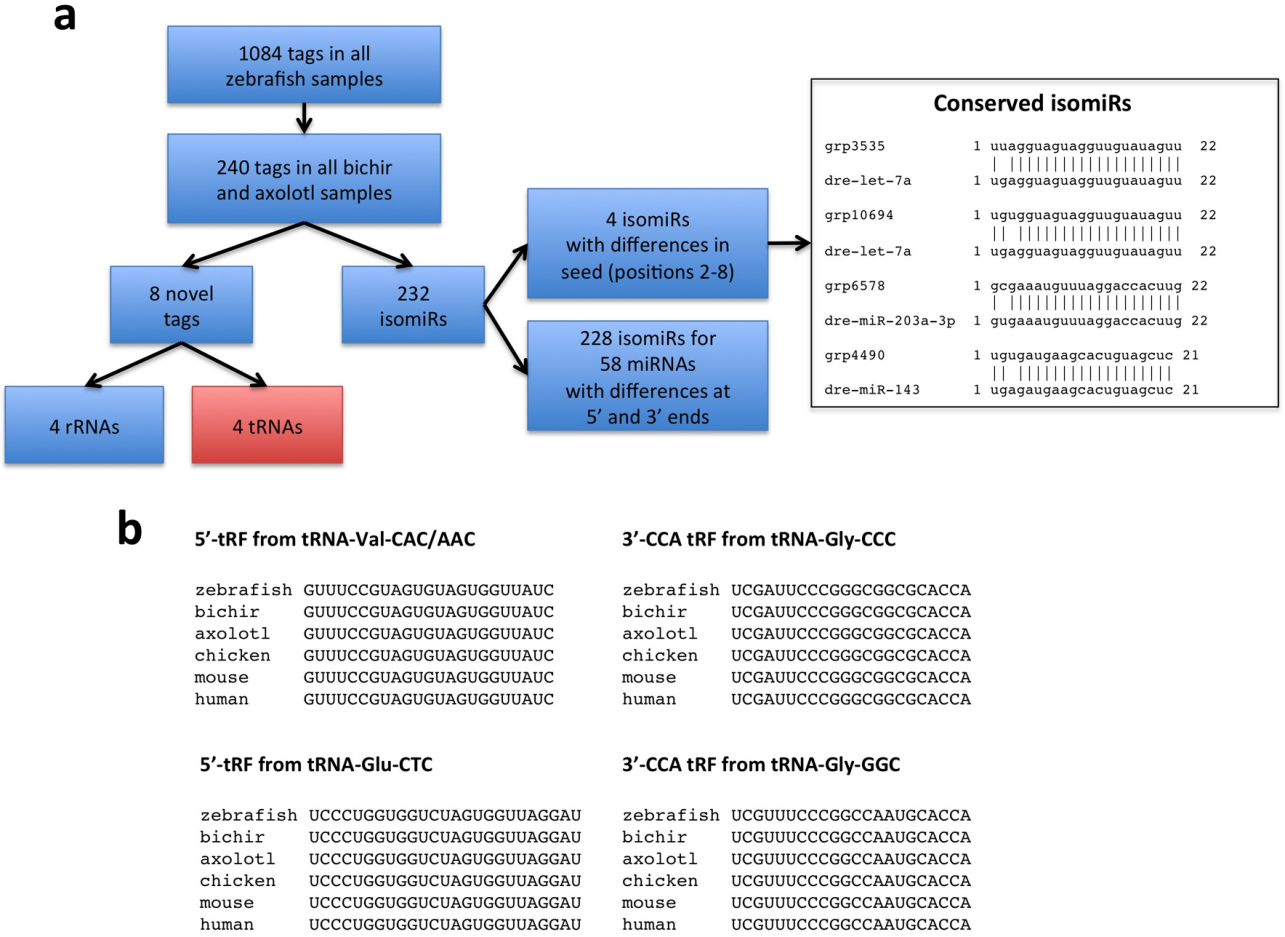
Analyses of conserved sequence tags reveal processed tRNA fragments and isomiRs. **(a)** Analysis workflow used to identify conserved sequence tags. Two-hundred-forty sequence tags expressed in all zebrafish samples were also expressed in all bichir and axolotl samples. 232 sequence tags were isomiRs including 4 that had substitutions in the seed sequence. Of the eight sequence tags were not derived from miRNAs, 4 mapped to ribosomal RNAs (rRNAs) and 4 were processed tRNA fragments. **(b)** The 4 processed tRNA fragments expressed in all zebrafish, bichir and axolotl samples. We found these processed tRNAs to also be small RNA sequence data from in chicken, mouse and human tissues [78].

To complement our analysis of known miRNAs, we also queried our dataset for 20-25-mer sequences that could represent potential novel miRNAs. We identified a total of 1,084 sequence tags that were represented in all zebrafish samples (S5–S8 Tables). Within the group, 240 sequence tags were shared with bichir and axolotl samples, of which, 232 sequence tags resembled isomiRs, mature miRNA products that differ from the canonical 5p or 3p sequences (S8 Table) [47]. Since variation in miRNA seed sequence can control distinct target genes, we further filtered our dataset to reveal isomiRs with sequence differences within positions 2-8-nt of the mature sequence. These filtering criteria revealed 4 isomiRs: 2 variants of let-7a and 1 variant of miR-203a-1-3p and miR-143 each (Fig 4A).

The remaining 8 sequence tags shared among zebrafish, bichir and axolotl have not been previously identified, and thus, represent novel sequence tags. This subset of putative novel small RNAs represents 4 rRNAs and 4 tRNAs (Fig 4A). Mature tRNA-derived noncoding RNAs are processed from precursor stem loop structures and integrated into Argonaute (Ago) complexes for translational repression in a manner analogous to known miRNAs [48–50]. Interestingly, a comparison of the 4 tRNAs with other species revealed sequence identity to several vertebrates, including humans (Fig 4B). Given that Ago-bound tRNAs have functional roles akin to miRNAs, it is intriguing to speculate that they may be important regulators of limb/appendage regeneration circuits. In sum, our analysis of 20-25-mer sequences revealed common subsets of differentially controlled miRNAs and potential novel miRNAs across zebrafish, bichir and axolotl.

### Network of consistently expressed genes with functional relationships to known blastema-associated genes

**Fig 5:**
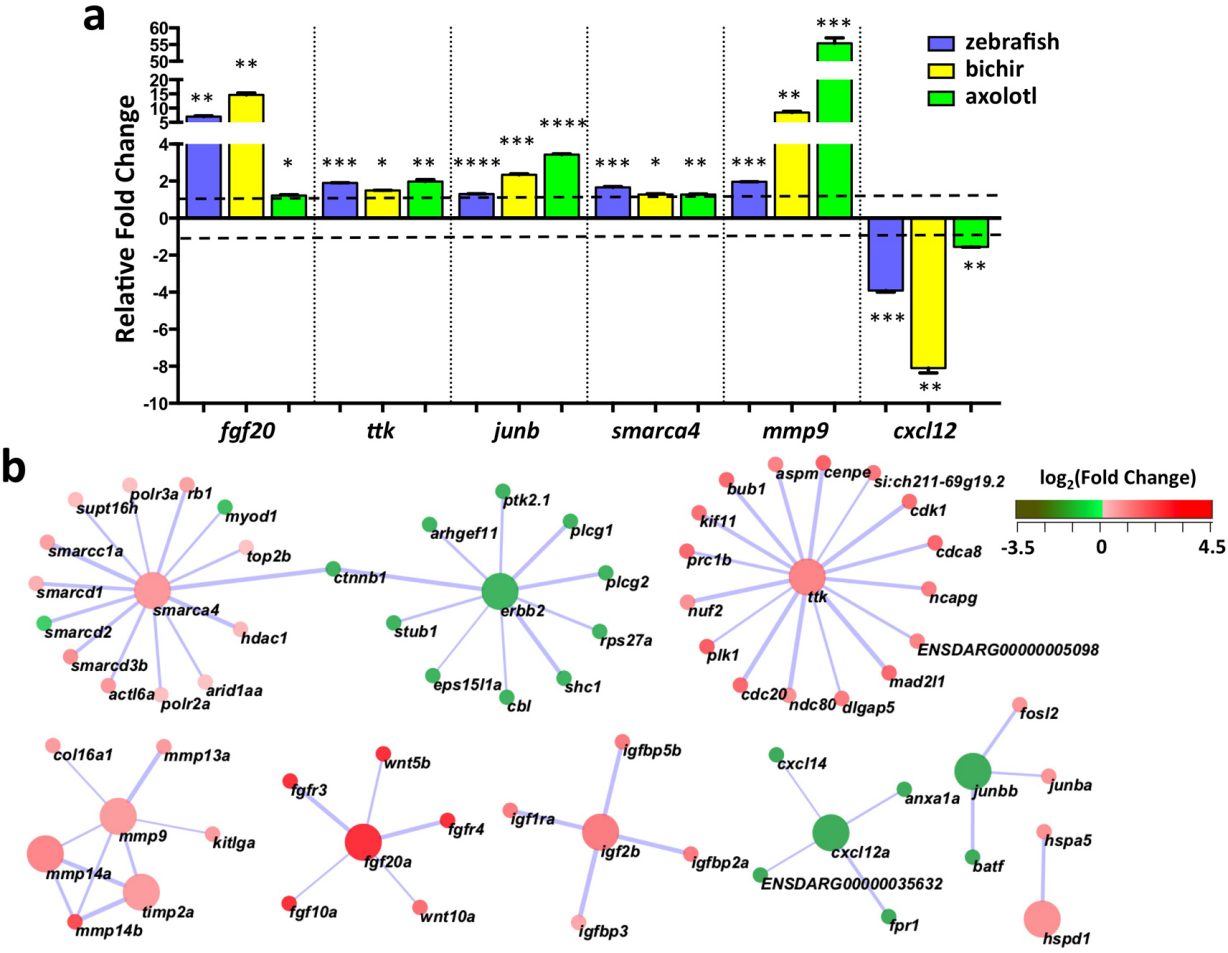
Commonly expressed blastema-associated genes. **(a)** qRT-PCR validation of common blastema-associated genes differentially expressed in regenerating zebrafish caudal fins (0 dpa vs. 4 dpa), bichir pectoral fins (0 dpa vs. 7 dpa) and axolotl forelimbs (0 dpa vs. 6 dpa), in triplicate. Each replicate contained at least 4 regenerating tissues. Average values +/- SEM are plotted. (*, **, *** = p-value < 0.01, 0.005 and 0.001 respectively). **(b)** STRING interactions among 11 blastema-associated genes (larger nodes) and 60 additional common differentially expressed genes (smaller nodes) with interaction scores greater than 98% of all 3,262 possible interactions. Nodes are colored according to fold-change observed in zebrafish and labeled according to zebrafish gene nomenclature provided by Ensembl.

In order to understand the regulatory mechanisms of miRNAs during limb/appendage regeneration, we complemented our small RNA analysis with mRNA gene expression studies. Since bichir and axolotl do not have genome sequences, we generated *de novo* transcriptome assemblies for regenerating bichir and axolotl tissues using Trinity software pipeline [51]. To improve these assemblies, we included RNA-Seq reads from two additional time points, 3 and 14 dpa. Together, the transcriptome assemblies contained a total of 94,273 components, transcript groups that resemble genes, for bichir and 73,787 components for axolotl (S9 Table). From these assemblies, we predicted homologous relationships for 9,598 (56.4%) of the 16,951 expressed zebrafish genes for both bichir and axolotl (S10–S14 Tables). We identified a set of 1,856 genes that were commonly upregulated in zebrafish, bichir and axolotl, and 1,345 that were downregulated (S15–S18 Tables). Among this filtered dataset, we identified 11 known blastema genes with differential expression patterns (S19 Table). Nine of these genes (*fgf20* [9], *hspd1* [10], *igf2* [52], *junb* [53], *mmp14* [11], *mmp9* [53], *smarca4* [53], *timp2* [54] and *ttk* [55]) had increased expression following injury while *cxcl12* [56] and *erbb2* [57]) displayed decreased expression (Table 3). Real-time qRT-PCR confirmed these expression changes in all three systems (Fig 5A).

**Table 3:** Eleven blastema-associated genes significantly differentially expressed during regenerating zebrafish caudal fin, bichir pectoral fins and axolotl forelimbs.

Symbol	Function	Zebrafish			Bichir		Axolotl	
		Fold Change	log(CPM)	FDR	Fold Change	log(CPM)	Fold Change	log(CPM)
***Growth Factor Signaling***								
*fgf20a*	FGF signaling	20.90	3.27	9.08e-52	8.97	1.87	1.80	1.49
*igf2b*	IGF signaling	3.84	6.28	5.52e-20	3.00	3.12	1.96	4.16
***Receptor Tyrosine Kinase***								
*erbb2*	Cell proliferation	-2.19	6.03	3.10e-19	-1.26	5.80	-1.23	6.02
***Protein Tyrosine Kinase***								
*ttk*	Cell proliferation	3.76	3.79	1.79e-17	>100	2.95	1.84	4.06
***Transcription Factors***								
*junbb*	Cell proliferation	2.59	6.91	1.28e-06	5.30	8.46	8.49	7.51
*smarca4*	Transcriptional coactivator	2.28	7.04	1.11e-17	2.50	5.34	2.29	5.18
***Extracellular Matrix (ECM)***								
*mmp9*	Proteolysis of ECM	2.33	6.98	2.03e-10	44.11	9.24	81.25	10.75
*mmp14a*	Positive regulator of cell growth	3.18	7.80	8.56e-31	3.39	8.80	3.18	8.76
*timp2a*	MMP Inhibitor	2.40	8.98	3.52e-18	1.37	7.71	1.36	7.63
***Chemokine***								
*cxcl12a*	Chemokine	-3.66	6.88	6.90e-17	-3.07	2.36	-6.30	2.48
***Chaperonin***								
*hspd1*	Stress response	1.69	7.07	9.24e-05	1.62	7.28	3.33	7.87

These 11 known blastema-associated genes were then used as a foundation to identify functional interactions with commonly expressed genes using STRING database [58]. STRING provides evidence of interactions among protein-coding genes based on physical protein interactions, gene co-expression and other functional associations. This analysis revealed a set of 1,550 genes from the 3,201 common upregulated and downregulated genes that had interactions with at least 1 of the 11 blastema-associated genes (S20 Table). Categorizing these genes by Gene Ontology (GO) terms, we identified three major classes of genes: 1) cell cycle process (GO:0022402) (p = 4.90 x 10^−14^), in which 72 genes were represented and 69 of which, were up-regulated, 2) regeneration (GO:0031099) (p = 2.15 x 10^−4^) harbored 24 annotated genes, 18 of which were upregulated and 3) cell migration (GO:0016477) (p = 9.15 x 10^−5^), which contained 57 annotated genes with 40 genes being up-regulated.

Within this network of 1,550 blastema-associated differentially expressed genes, we identified a subset of 71 genes that had interaction scores greater than the top 2% of all 3,262 interactions, suggesting critical roles during blastema formation (Fig 5B). These genes included β-catenin (*ctnnb1*) that links *erbb2* with *smarca4*, additional extracellular matrix genes (*mmp13* and *col16a1*), FGF receptors (*fgfr3* and *fgfr4*) and *fgf10*, IGF signaling pathway members (*igfbp2*, *igfbp3*, *igfbp5* and *igf1r*) and the *cxcl14* chemokine. Taken together, our studies of mRNA expression changes across 3 regenerating limb/appendage systems extend our understanding of the genetic circuits of regeneration by identifying previously unassociated blastema genes.

### Subset of network targeted by common regulated miRNAs

In order to understand the potential influence of miRNAs on blastema-associated gene expression, we identified a subset of genes with predicted miRNA binding sites in the 3’-UTR that exhibit expression patterns inversely correlated with miRNA expression. These filtering criteria identified 136 downregulated genes with predicted binding sites in the 3’-UTRs for any of the 5 common upregulated miRNAs (miR-21, miR-31, miR-181b, miR-181c and miR-7b) (S21 Table). Fifty-eight of the 136 genes were members of the blastema-associated network, of which, 5 genes: *tgfbr2*, *erbb2*, *efna5*, activin receptor a type IIA (*acvr2a*) and yes-related kinase (*yrk*) represented the enriched GO Biological Process term “enzyme linked receptor protein signaling pathway” (GO:0007167) (p = 4.80x10^-4^) (S22 Table).

**Fig 6:**
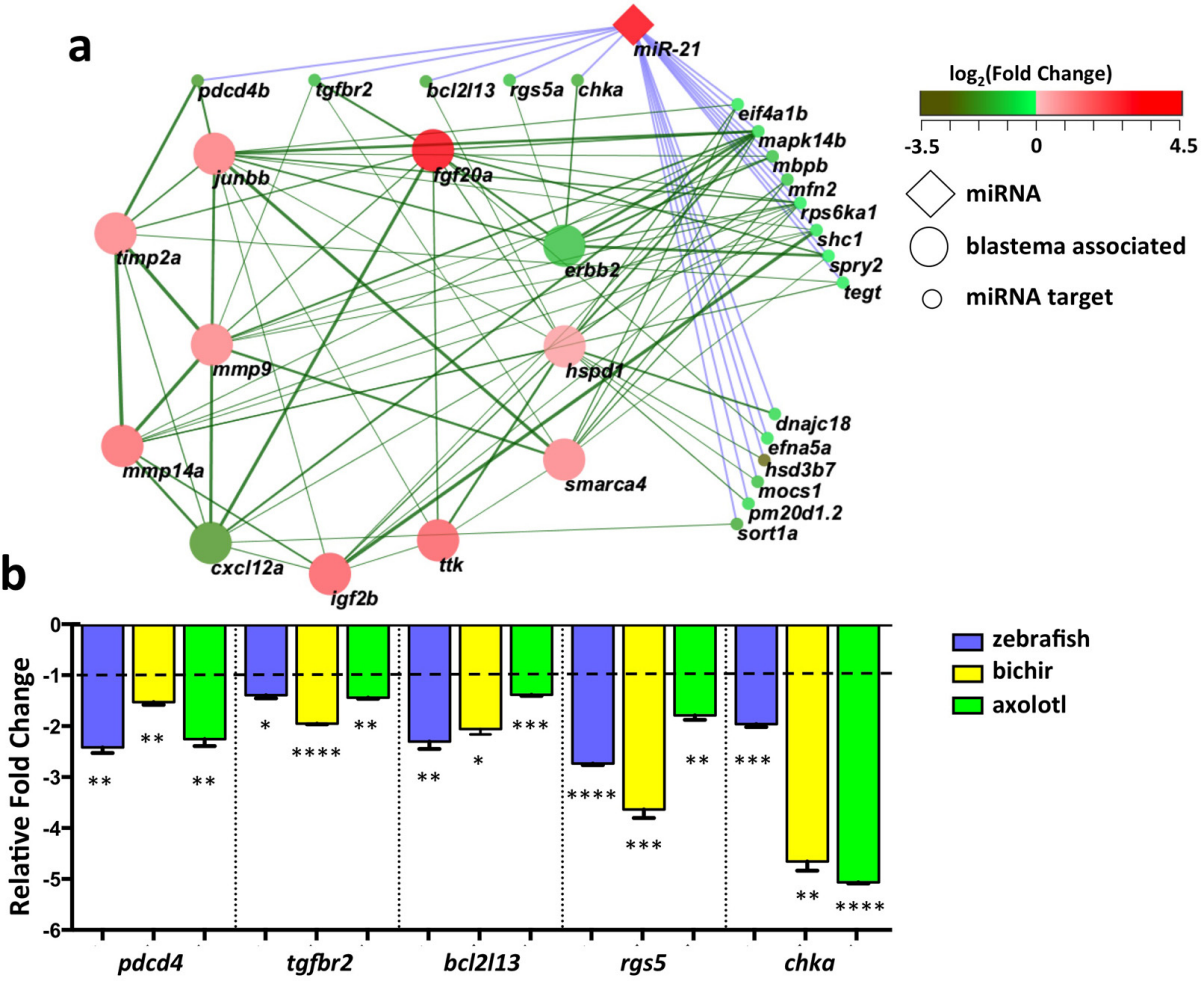
miR-21 regulation of blastema-associated genes. **(a)** Network of blastema-associated genes, miR-21 and predicted miR-21 targets differentially expressed in three model systems. STRING interactions with 11 common blastema-associated genes, miR-21, miR-31, miR-181, and 50 additional common differentially expressed genes with common predicted miRNAs binding sites. STRING interactions are scaled by the thickness and color of edges score. Nodes representing the miRNAs, predicted target mRNAs and blastema-associated genes are colored according to fold change observed in zebrafish and labeled according to zebrafish gene nomenclature provided by Ensembl. **(b)** qRT-PCR validation of five common predicted miR-21 target genes downregulated in regenerating zebrafish caudal fins (0 dpa vs. 4 dpa), bichir pectoral fins (0 dpa vs. 7 dpa) and axolotl forelimbs (0 dpa vs. 6 dpa). Each data point represents average values +/- SEM from triplicate reactions, each containing at least 4 biological samples. (*, **, *** = p-value < 0.01, 0.005 and 0.001 respectively).

Among the differentially upregulated miRNAs across all three regenerating systems, miR-21 was the most highly expressed and upregulated miRNA (Figs 2C and 3A). We identified a subset of 19 downregulated transcripts from the 58 blastema-associated genes with a predicted miR-21 binding site (Fig 6A and S2 File). Using real-time qPCR studies, we confirmed the downregulated expression of *pdcd4* [29], *tgfbr2* [59], *rgs5* [60], *chka* [61] and *bcl2l13* [62] (Fig 6B). Both *pdcd4* and *tgfbr2* were chosen for validation because they are known miR-21 targets. The other three genes, *bcl2l13*, *rgs5* and *chka*, were selected because we predicted them to be targeted by both miR-21 and miR-181c and they have inhibitory roles in cellular proliferation.

Interestingly, *erb-b2* receptor tyrosine kinase 2 (*erbb2*) was the only blastema-associated transcript predicted to be targeted by one of the common regulated miRNAs, miR-181b and miR-181c. *erbb2* is required for cellular proliferation and migration during appendage regeneration [57] and, was recently shown to be essential for cardiac regeneration [62]. Ephrin 5 (*efna5*), a receptor protein tyrosine kinase that inhibits axon bundling during outgrowth [63] was the only gene targeted by all 5 miRNAs. In addition to *rgs5*, both *bcl2l13* and *chka* had predicted binding sites for 4 miRNAs (miR-21, miR-181b, miR-181c and miR-7b).

We performed similar analyses to capture potential target genes for the 5 commonly downregulated miRNAs (miR-2184, miR-204, miR-338, miR-133a and miR-24). Our studies revealed 205 candidate genes, 107 of which are members of the blastema-associated network (S23 and S24 Tables). Extracellular matrix genes were enriched (p = 9.84 x 10^−4^) among the subset of 107 genes, including *mmp9*, *mmp13*, *mmp14*, laminin β4 (*lamb4*) and *timp2*. Ten genes were identified among those with the top 2% of interactions in the network, including *fgf10*, which is predicted to be targeted by miR-204, miR-338 and miR-2164. Three of the 107 genes are previously identified targets of the downregulated miRNAs, including *mmp14*, a known target of miR-133 [64], *mmp9* (targeted by miR-204 and miR-338) and *timp2* (targeted by miR-24 and miR-204). In addition, our analyses revealed 4 transcripts, *dot1l*, *fgf10*, *ptprf* and *SLC7A5*, which contain predicted binding sites for 3 miRNAs while 11 mRNAs were predicted to be regulated by 2 miRNAs (S24 Table). Taken together, our studies implicate evolutionarily conserved putative miRNA-mRNA regulatory units as important stimulators of blastema formation during limb/appendage regeneration.

## Discussion

**Fig 7:**
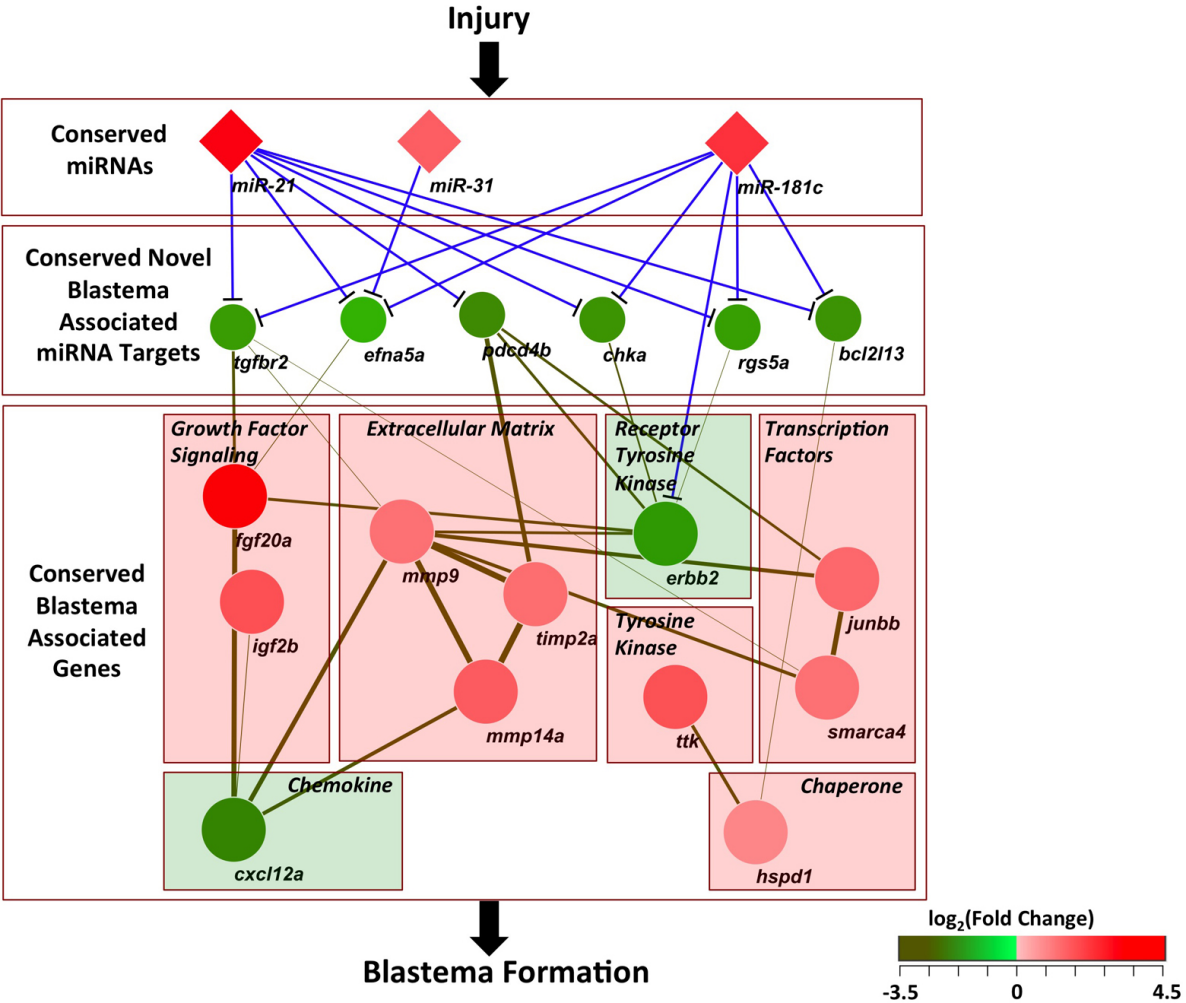
Conserved gene regulatory circuit for appendage regeneration. Following injury, three miRNAs (miR-21, miR-31 and miR-181c) are commonly upregulated and target a set of five commonly downregulated genes shown with blue lines. These five target genes have functional interactions (green lines) with a set of 11 common blastema-associated genes from STRING [58]. Higher interaction scores are shown by thicker edges with darker green colors. These blastema-associated genes have diverse functions including growth factor signaling, extracellular matrix remodeling, signal transduction, transcription and protein homeostasis. Nodes representing the miRNAs and mRNAs are colored according to fold change observed in zebrafish and labeled according to zebrafish gene nomenclature provided by Ensembl.

In this study, we performed transcriptome-profiling studies at the equivalent regenerative stage for the zebrafish caudal fin, bichir pectoral fin and axolotl forelimb in an effort to identify expression changes in genetic programs that accompany blastema formation. Our comparative studies identified a multi-layered genetic network defined by 8 shared, differentially regulated miRNAs and their putative target genes. As miR-21 is the most highly upregulated and most highly expressed miRNA in all three models, we further investigated miR-21 target genes. This analysis identified 2 known miR-21 target genes (*pdcd4* and *tgfbr2*) and 3 putative novel targets (*bcl2l13*, *rgs5* and *chka*) that are implicated during blastema formation. Fig 7 summarizes the gene regulatory circuit for miR-21, miR-31 and miR-181c, the 3 validated, shared upregulated miRNAs, with downregulated putative target genes that have functional relationships with conserved blastema-associated genes. Upregulation of miR-21, miR-31 and miR-181c leads to the downregulation of inhibitors and suppressors such as *pdcd4*, *tgfbr2*, *bcl2l13*, *rgs5* and *chka*, downregulated genes with anti-proliferative functions.

Although our study focused on identification of similarly regulated miRNAs, we also noted a subset of miRNAs that exhibit either different expression changes or no change in expression. For instance, while miR-181b and miR-7 levels were highly upregulated in injured zebrafish and bichir fins, analysis of regenerating axolotl forelimbs showed expression levels were significantly downregulated. It is possible that this difference is a reflection of differences in cell types and/or regenerative mechanisms that are independent of cellular dedifferentiation. Skeletal muscle, while absent in zebrafish and bichir fins, is highly abundant in the axolotl limb. Although transplant studies from the Tanaka lab showed that the primary contribution for muscle regeneration is via dedifferentiation of myotubes [65], it is possible that activation of uncharacterized progenitor cells could be an accompanying mechanism during regeneration [66–68]. Alternatively, miRNAs that do not exhibit significant changes in expression are likely to have injury independent roles during tissue repair, perhaps functioning in tissue homeostasis.

The network of conserved miRNAs and target genes identified in this study, serves as a platform to inform new hypotheses about miRNA regulation of limb/appendage regeneration. Morphological and histological studies of miR-21, miR-31 and/or miR-181 inhibition combined with identification of target genes would demonstrate their roles in blastema formation. Previous profiling studies have also identified miR-21 as a highly upregulated miRNA in response to injury in multiple tissues and numerous organisms [2,27,41,69]. Defining miR-21 mechanism of action in these different contexts thus provide an important opportunity with which to define the cellular and molecular mechanisms of endogenous repair and regeneration processes that have been conserved during evolution.

This comparative genomic study also revealed novel, conserved miRNAs. Mature tRNA-derived noncoding RNAs are a new class of regulators that are processed in an Ago-Dicer complex, akin to miRNA processing [48–50]. Interestingly, the 4 tRNAs we identified in our studies also have identical sequences in organisms as complex as humans (Fig 5). Given the steady-state expression in regeneration systems and in animals with limited regenerative capacity, it is likely that tRNAs may be important for tissue homeostasis rather than regenerative proliferation. Future functional studies will provide valuable insight into the contributions of these tRNAs. To the best of our knowledge, this is the first comparative study to identify evolutionarily conserved miRNA-mRNA regulatory circuits during limb/appendage regeneration in 3 animal systems.

## Conclusions

Limb/appendage regeneration is guided primarily by dedifferentiation of spared tissues proximal to the plane of injury. Although there are fundamental differences in tissue composition and complexity among zebrafish caudal fins, bichir pectoral fins and axolotl forelimbs, our study suggests that natural cellular reprogramming of differentiated cells during regeneration is guided by a core group of shared, differentially controlled miRNAs. Given that miRNAs exert effects on biology through cell autonomous and non-autonomous mechanisms, future studies revealing the spatial distribution of these gene regulators will illuminate the extent that these shared circuits are similar and different among the model systems. Together, our study demonstrates the high value of framing studies in a comparative biological context, an approach that is often overlooked in the development and application of computational strategies.

## Methods

### Animal husbandry and tissue collection

All animal studies were conducted under approved MDI Biological Laboratory IACUC protocols assigned to V.P.Y. Anesthesia and euthanasia of animals were performed with an overdose of MS-222. Adult Ekkwill zebrafish (MDI Biological Laboratory), bichir (Maine Pet and Aquarium, Ellsworth, ME) and axolotl (Ambystoma Genetic Stock Center, Lexington, KY) were housed at the MDI Biological Laboratory Animal Core and maintained at 27°C, 27°C and 18°C respectively, in a 14:10 hour light/dark cycle. Approximately, 50% of limbs/appendages were amputated with scalpels following anesthesia (0.6mM MS222). Tissues were collected from zebrafish caudal fins at 0 and 4 days post-amputation (dpa), bichir pectoral fins at 0, 3, 7 and 14 dpa and axolotl forelimbs at 0, 3, 6 and 14 dpa. Injured animals were returned to recirculating housing systems appropriate for each animal and monitored daily for the first 3 days to ensure they exhibit normal swimming and feeding behavior. Animals that did not meet these metrics were euthanized.

### RNA preparation and sequencing

Total RNA samples were extracted from tissue sample using TRI Reagent (Molecular Research Center, Inc., Cincinnati, OH) following manufacturer’s protocol. For zebrafish caudal fin RNA samples, barcoded Illumina (San Diego, CA) TruSeq small RNA libraries were prepared and sequenced on a single lane of an Illumina HiSeq2000 at Delaware Biotechnology Institute following manufacturer’s protocols. Indexed strand-specific polyA+ selected mRNA libraries were prepared and paired-end sequenced on an Illumina HiSeq2500 at HudsonAlpha Institute for Biotechnology following manufacturer’s protocols. These experiments were performed in triplicate, with each replicate consisting of 5–8 biological fin samples. For each bichir pectoral fin and axolotl forelimb RNA samples, both small RNA and mRNA libraries were prepared and sequenced using an Illumina Genome Analyzer IIx at the Centre for Applied Genomics, Hospital for Sick Children, Toronto, Canada. These experiments were conducted with a single replicate consisting of at least 4-pooled biological tissues for each stage.

### miRNA annotation and analysis

Small RNA-Seq reads were adapter clipped, trimmed by quality and collapsed into tags using the FASTX Toolkit (http://hannonlab.cshl.edu/fastx_toolkit/). Known miRNAs in the set of 20-25-mer sequence tags were annotated using miRMiner and miRBase version 21 for bichir and axolotl. Known and novel zebrafish miRNAs were annotated using miRMiner with miRBase version 21 and the Zv9 zebrafish genome assembly [70]. Sequence tag counts for the annotated miRNAs were analyzed using R/edgeR [71].

### qRT-PCR validation of miRNAs

Expression of candidate miRNAs and mRNAs were assayed using quantitative real-time PCR (qRT-PCR). miRNA and mRNA cDNAs were prepared from total RNA samples using the qScript miRNA and mRNA cDNA Synthesis Kits, respectively (Quanta, Gaithersburg, MD). qRT-PCR reactions were performed in triplicate, using SYBR Green Brilliant III QPCR Master Mix (Agilent, Santa Clara, CA) on a LightCycler 480 System (Roche Diagnostics, Indianapolis, IN). All primers used are listed in S25 Table.

### *De novo* transcriptome assembly and analysis

Bichir and axolotl RNA-Seq reads were adapter clipped and quality trimmed using Trimmomatic [72] and assembled using Trinity [51]. RNA-Seq reads from each sample were mapped to the respective assembly using RSEM [73] to generate read counts per transcript per sample. Read counts were analyzed using R/edgeR [71] to determine those that were differentially expressed.

### miRNA target prediction

miRNA targets were predicted by analyzing annotated 3’-UTR sequences from Ensembl version 76 [74] for zebrafish and open reading frame predictions for bichir and axolotl using miRanda [75]. Target predictions were made using mature miRNA sequences and mature miRNA sequences that allow a nucleation bulge at positions 5–6 [76] for the common upregulated and downregulated miRNAs (S3 File).

### Gene set enrichment

Gorilla [77] was used to test for enriched GO terms among gene lists using zebrafish orthologs. The background used for both analyses were the zebrafish orthologs of the 9,598 commonly expressed genes.

### Data Access

Zebrafish, bichir and axolotl gene expression data are accessible from NCBI Gene Expression Omnibus under accession numbers GSE74415, GSE74374 and GSE74372, respectively. Short RNA and mRNA sequence data for zebrafish, bichir and axolotl are accessible from the NCBI Short Read Archive under accession numbers SRP065355, SRP065300 and SRP065299, respectively.

### Statistical analysis

All statistics were performed using Student’s t-test with Welch’s correction. A p-value< 0.05 was deemed statistically significant.

## Supporting Information

S1 Fig Overview of shared and unique miRNAs detected in regenerating appendages following blastema formation in bichir pectoral fins and axolotl forelimbs. Venn diagram shows 42 miRNAs are shared between bichir and axolotl, approximately 50% of all miRNAs identified from each respective genome.
(JPG)

S1 File Annotated miRNAs expressed in zebrafish, bichir and axolotl during limb/appendage regeneration. **(a)** Precursor and mature sequences for miRNAs expressed during zebrafish caudal fin regeneration annotated from small RNA sequence data. **(b)** Zebrafish mature sequences used for differential miRNA analysis. **(c)** Mature miRNA sequences expressed during bichir appendage regeneration annotated from small RNA sequence data. **(d)** Mature miRNA sequences expressed during bichir appendage regeneration annotated from small RNA sequence data.
(DOC)

S2 File Predicted miR-21 binding sites for genes shown in Fig 6A. Predicted miR-21 binding sites predicted by miRanda [75] within 3’-UTRs for zebrafish, bichir and axolotl target genes. The 3’-UTRs were taken from Ensembl (version 76) for zebrafish while the 3’-UTRs for bichir and axolotl were derived from open reading frame predictions from annotated *de novo* transcriptome assemblies.
(DOC)

S3 File Mature miRNA sequences used for miRNA target prediction. Sequences of mature miRNAs for the common up- and downregulated miRNAs along with sequences that allow a nucleation bulge at positions 5–6 [76] for zebrafish, bichir and axolotl.
(DOC)

S1 Table miRNAs expressed in regenerating zebrafish caudal fins, bichir pectoral fins and axolotl forelimbs. One-hundred-eight common miRNAs were found to be expressed in zebrafish, bichir and axolotl regenerating limb/appendages. Sixteen common miRNAs were expressed in both zebrafish and bichir, but not axolotl. Another 16 miRNAs were expressed in common between bichir and axolotl, but not zebrafish. Three common miRNAs were expressed in zebrafish and axolotl, but not bichir. Twenty-one, 14 and 16 miRNAs were uniquely expressed in zebrafish, bichir and axolotl, respectively.
(XLSX)

S2 Table miRNA expression profiles from zebrafish appendage regeneration. Mature miRNA expression analysis results for regenerating zebrafish caudal fins at 4 dpa compared to 0 dpa. Fold-change, statistics and normalized read counts are given for the most highly expressed mature miRNA product. Fold-change is expressed as log_2_. The average level of normalized expression across all six samples are expressed as log_2_ counts per million (cpm). Statistics include the p-value and false discovery rate (FDR) adjusted p-value.
(XLSX)

S3 Table miRNA expression profiles from bichir appendage regeneration. Mature miRNA expression analysis results for regenerating bichir pectoral fins at 7 dpa compared to 0 dpa. Fold-change is expressed as log_2_. The average level of normalized expression across 0 dpa, 3 dpa, 7 dpa and 14 dpa samples and normalized read counts per sample are reported as expressed as log_2_ cpm. Statistics include the p-value and false discovery rate (FDR) adjusted p-value.
(XLSX)

S4 Table miRNA expression profiles from axolotl limb regeneration. Mature miRNA expression analysis results for regenerating axolotl forelimbs at 6 dpa compared to 0 dpa. Fold-change is expressed as log_2_. The average level of normalized expression across all six samples and normalized read counts per sample are reported as expressed as log_2_ cpm. Statistics include the p-value and false discovery rate (FDR) adjusted p-value.
(XLSX)

S5 Table Small RNA expression profiles from zebrafish during appendage regeneration. Small RNA expression analysis results for regenerating zebrafish caudal fins at 4 dpa compared to 0 dpa. Fold-change, statistics, normalized read counts and tag sequence is given for each small RNA tag. Fold-change is expressed as log_2_. The average level of normalized expression across all six samples are expressed as log_2_ counts per million (cpm). Statistics include the p-value and false discovery rate (FDR) adjusted p-value.
(XLSX)

S6 Table Small RNA expression profiles from bichir during appendage regeneration. Small RNA expression analysis results for regenerating bichir pectoral fins at 7 dpa compared to 0 dpa. Fold-change is expressed as log_2_. The average level of normalized expression across 0 dpa, 3 dpa, 6 dpa and 14 dpa samples and normalized read counts per sample are reported as expressed as log_2_ cpm. Statistics include the p-value and false discovery rate (FDR) adjusted p-value.
(XLSX)

S7 Table Small RNA expression profiles from axolotl during limb regeneration. Small RNA expression analysis results for regenerating axolotl forelimbs at 6 dpa compared to 0 dpa. Fold-change is expressed as log_2_. The average level of normalized expression across 0 dpa, 3 dpa, 6 dpa and 14 dpa samples and normalized read counts per sample are reported as expressed as log_2_ cpm. Statistics include the p-value and false discovery rate (FDR) adjusted p-value.
(XLSX)

S8 Table Small RNA sequence tags expressed in all zebrafish samples. Expression data for all 1,084 20-25-mer small RNA sequence tags expressed in all zebrafish samples together with the number of reads that the same 20-25-mer was detected in each bichir and axolotl sample. Fold-change, statistics, normalized read counts and tag sequence is given for each small RNA tag in the zebrafish. Fold-change is expressed as log_2_. The average level of normalized expression across all six samples are expressed as log_2_ counts per million (cpm). Statistics include the p-value and false discovery rate (FDR) adjusted p-value. Non-normalized read counts are reported per sample. For bichir and axolotl samples, the read counts are listed after the hyphen character. The number preceding the hyphen is a species-specific sequence tag identifier.
(XLSX)

S9 Table Summary statistics of bichir and axolotl *de novo* transcriptome assemblies. Statistics are given for bichir and axolotl assemblies as reported from the Trinity assembly software. For example, the number of transcripts, N50 length and percent of CEGMA proteins found in the assembly are reported.
(XLSX)

S10 Table mRNA gene expression profiles of regenerating zebrafish caudal fins. mRNA expression analysis results for regenerating zebrafish caudal fins at 4 dpa compared to 0 dpa. Fold-change, statistics and normalized read counts are given for each gene annotated by Ensembl (version 76). Fold-change is expressed on a linear and log_2_ scale. The average level of normalized expression across all six samples are expressed as log_2_ counts per million (cpm). Statistics include the log ratio (LR), p-value and false discovery rate (FDR) adjusted p-value. Annotation includes Ensembl gene ID, gene symbol, description and chromosome coordinates. Normalized read counts are reported per sample as log_2_ cpm.
(XLSX)

S11 Table mRNA gene expression profiles of regenerating bichir pectoral fins. mRNA expression analysis results for regenerating bichir pectoral fins at 7 dpa compared to 0 dpa. Fold-change is expressed as log_2_. The average level of normalized expression across 0 dpa, 3 dpa, 7 dpa and 14 dpa samples and normalized read counts per sample are reported as expressed as log_2_ cpm.
(XLSX)

S12 Table mRNA gene expression profiles of regenerating axolotl forelimbs. mRNA expression analysis results for regenerating axolotl forelimbs at 7 dpa compared to 0 dpa. Fold-change is expressed as log_2_. The average level of normalized expression across 0 dpa, 3 dpa, 7 dpa and 14 dpa samples and normalized read counts per sample are reported as expressed as log_2_ cpm.
(XLSX)

S13 Table Relationships between zebrafish protein-coding genes and assembled transcriptome sequences from.bichir. Identifiers of zebrafish protein-coding genes and transcriptome assembly contig identifiers of predicted orthologs. The log_2_ fold-change of the bichir transcript is also shown.
(XLSX)

S14 Table Relationships between zebrafish protein-coding genes and assembled transcriptome sequences from axolotl. Identifiers of zebrafish protein-coding genes and transcriptome assembly contig identifiers of predicted orthologs. The log_2_ fold-change of the axolotl transcript is also shown.
(XLSX)

S15 Table Common upregulated mRNAs during zebrafish and bichir appendage regeneration. Common upregulated mRNAs shown as a mapping between bichir transcripts and zebrafish genes. Fold-changes are shown for bichir along with zebrafish fold change, statistics, gene symbol and description.
(XLSX)

S16 Table Common upregulated mRNAs during zebrafish and axolotl limb/appendage regeneration. Common upregulated mRNAs shown as a mapping between axolotl transcripts and zebrafish genes. Fold-changes are shown for axolotl along with zebrafish fold change, statistics, gene symbol and description.
(XLSX)

S17 Table Common downregulated mRNAs during zebrafish and bichir appendage regeneration. **(a, b)** Common downregulated mRNAs shown as a mapping between bichir transcripts and zebrafish genes. Fold-changes are shown for bichir along with zebrafish fold change, statistics, gene symbol and description.
(XLSX)

S18 Table Common downregulated mRNAs during zebrafish and axolotl limb/appendage regeneration. Common downregulated mRNAs shown as a mapping between axolotl transcripts and zebrafish genes. Fold-changes are shown for axolotl along with zebrafish fold change, statistics, gene symbol and description.
(XLSX)

S19 Table mRNA expression profiles of bichir and axolotl genes predicted to be orthologous to zebrafish genes previously associated with blastema formation in zebrafish. Genes orthologous to blastema-associated genes in the zebrafish were identified that were commonly up- or downregulated in bichir and/or axolotl. Genes commonly upregulated in all three models are shaded red. Genes commonly downregulated in all three models are shaded green. Genes up- or downregulated in zebrafish and bichir are shaded pink. Genes up- or downregulated in zebrafish and axolotl are shaded blue. As multiple transcripts may represent the same gene in the bichir and axolotl assemblies, we selected the transcript that had the highest average level of expression (log_2_ cpm). Other transcripts that map to the gene are shaded gray.
(XLSX)

S20 Table Network of commonly up- and downregulated genes with functional interactions to eleven blastema-associated genes. Set of 1,550 common up- and downregulated genes that had interactions with at least one of 11 blastema-associated genes according to STRING. The network of 1,561 genes had 3,262 interactions with a total of 1,012 genes were commonly upregulated and 549 were commonly downregulated. Representative zebrafish gene IDs, symbols, descriptions and fold changes are listed for each interaction.
(XLSX)

S21 Table Common downregulated genes predicted to be targeted by five common upregulated miRNAs. Zebrafish Ensembl gene identifiers for 136 genes downregulated in three models with predicted miRNA binding sites for miR-21, miR-181c, miR-181b, miR-31 or miR-7 in all three models. The miRNAs predicted to bind the 3’-UTR for each target gene are shown next to the Ensembl gene ID and gene symbol.
(XLSX)

S22 Table Subset of common downregulated genes predicted to be targeted by five common upregulated miRNAs that were members of the network. Zebrafish Ensembl gene identifiers for 58 genes downregulated in three models with predicted miRNA binding sites for miR-21, miR-181c, miR-181b, miR-31 and miR-7 and members of the network of commonly up- and downregulated genes with functional interactions to 11 blastema-associated genes. The miRNAs predicted to bind the 3’-UTR for each target gene are shown next to the Ensembl gene ID and gene symbol.
(XLSX)

S23 Table Common upregulated genes predicted to be targeted by five common downregulated miRNAs. Zebrafish Ensembl gene identifiers for 205 genes upregulated in three models with predicted miRNA binding sites for miR-2184, miR-204, miR-338, miR-133a and miR-24 in all three models. The miRNAs predicted to bind the 3’-UTR for each target gene are shown next to the Ensembl gene ID and gene symbol.
(XLSX)

S24 Table Subset of common upregulated genes predicted to be targeted by five common downregulated miRNAs that were members of the network. Zebrafish Ensembl gene identifiers for 107 genes upregulated in three models with predicted miRNA binding sites for miR-2184, miR-204, miR-338, miR-133a and miR-24 and members of the network of commonly up- and downregulated genes with functional interactions to 11 blastema-associated genes. The miRNAs predicted to bind the 3’-UTR for each target gene are shown next to the Ensembl gene ID and gene symbol.
(XLSX)

S25 Table qRT-PCR primer sequences. Oligonucleotide sequences used for qRT-PCR reactions for each miRNA and mRNA assayed in zebrafish, bichir and axolotl regenerating samples.
(XLSX)
