# Supplementary figures and images for: A Conserved MicroRNA Regulatory Circuit Is Differentially Controlled during Limb/Appendage Regeneration
Source: PLoS One. 2016 Jun 29;11(6):e0157106. doi: 10.1371/journal.pone.0157106 (PMC4927183; doi:10.1371/journal.pone.0157106)

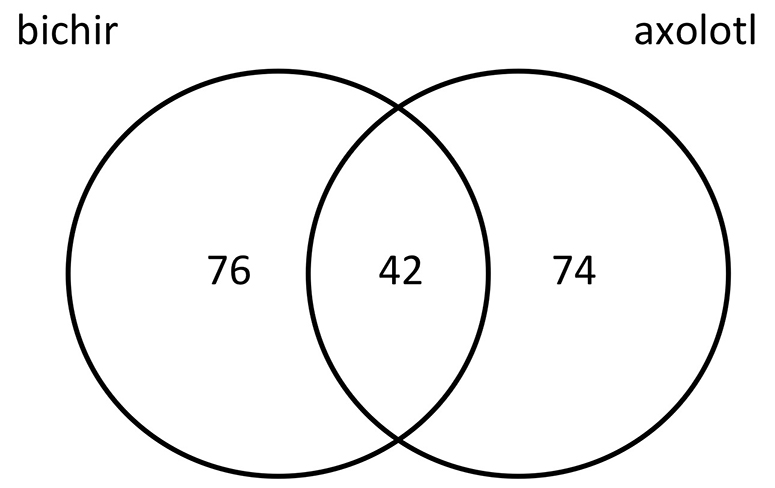

Supplement: S1 Fig — Venn diagram shows 42 miRNAs are shared between bichir and axolotl, approximately 50% of all miRNAs identified from each respective genome. (JPG) [file pone.0157106.s001.jpg]
