# Supplementary material for: A Conserved MicroRNA Regulatory Circuit Is Differentially Controlled during Limb/Appendage Regeneration
Source: PLoS One. 2016 Jun 29;11(6):e0157106. doi: 10.1371/journal.pone.0157106 (PMC4927183; doi:10.1371/journal.pone.0157106)
Supplement: S1 File — (a) Precursor and mature sequences for miRNAs expressed during zebrafish caudal fin regeneration annotated from small RNA sequence data. (b) Zebrafish mature sequences used for differential miRNA analysis. (c) Mature miRNA sequences expressed during bichir appendage regeneration annotated from small RNA sequence data. (d) Mature miRNA sequences expressed during bichir appendage regeneration annotated from small RNA sequence data. (DOC) [file pone.0157106.s002.doc]

**S1 File: Annotated miRNAs expressed in zebrafish, bichir and axolotl during limb/appendage regeneration.**

A) Precursor and mature sequences for miRNAs expressed during zebrafish caudal fin regeneration annotated from small RNA sequence data.

>dre-miR-21-1-pre 10:28880695-28880834- CCCAGTGTGTCAGATAGCTTATCAGACTGGTGTTGGCTGTTACATTCGCCCGGCGACAACAGTCTGTAGGC TGTCTGACATTTTGGG

>dre-miR-21-1-5p grp3295-4000898

TAGCTTATCAGACTGGTGTTGGC

>dre-miR-21-1-3p grp13680-1476

CGACAACAGTCTGTAGGCTGTC

>dre-miR-21-2-pre 15:16392775-16392914+ GTCCCTTTCTGTCATGTAGCTTATCAGACTGGTGTTGGCTTTGAGTTTTTGGCAACAGCAGTCTAATAGGC TGTCTGACATTTTGGGC

>dre-miR-21-2-5p grp3295-4000898

TAGCTTATCAGACTGGTGTTGGC

>dre-miR-462-pre 8:26935199-26935338+ GCAGTCTGGATGGTAACGGAACCCATAATGCAGCTGTTTGGTTGATTGATTGACAGCTGTCTGTGGATTCC GTGCCCCTCCTTTCTTGC

>dre-miR-462-5p grp6105-1106777

TAACGGAACCCATAATGCAGCTG

>dre-miR-462-3p grp12277-1060

GCTGTCTGTGGATTCCGTGCCC

>dre-miR-181a-1-pre 22:24551958-24552097- GGCCACCGTTTGCCTCAGTGAACATTCAACGCTGTCGGTGAGTTTGAGCTAAATGGAAAAAAACCATCGAC CGTTGATTGTACCCTGCGGCCGAGCC

>dre-miR-181a-1-5p grp9677-889187

AACATTCAACGCTGTCGGTGAG

>dre-miR-181a-1-3p grp2272-13850

ACCATCGACCGTTGATTGTACC

>dre-miR-181a-2-pre 8:55451653-55451792- TGGTCCCTTGGTGAACATTCAACGCTGTCGGTGAGTTTTGCGCTTCTGTAACAAACCATCGACCGTTGACT GTACCCTGAGGGTGGCCA

>dre-miR-181a-2-5p grp9677-889187

AACATTCAACGCTGTCGGTGAG

>dre-miR-181a-2-3p grp8551-5491

ACCATCGACCGTTGACTGTACC

>dre-miR-181a-3-pre 2:45255022-45255161- (NOVEL) GCTGGTCTCGCAGAACATTCAACGCTGTCGGTGAGTTTTAGACATTGAAAAACCATCGACCGTTGACTGTG CCCTGAGGCCACGC

>dre-miR-181a-3-5p grp9677-889187

AACATTCAACGCTGTCGGTGAG

>dre-miR-181a-3-3p grp10805-11348

ACCATCGACCGTTGACTGTGCCt

>dre-miR-181a-4-pre 3:34282695-34282834+ (NOVEL) TGGTGCATTCTGGGAACATTCAACGCTGTCGGTGAGTTTGGATGCATAATAAACCATCGACCGTTGGCTGT GCCCTGAGATTACCA

>dre-miR-181a-4-5p grp9677-889187

AACATTCAACGCTGTCGGTGAG

>dre-miR-181a-4-3p grp5867-157

ACCATCGAGTGTTGAGTGTACC

>dre-miR-181a-5-pre 21:7721488-7721627- (NOVEL) TGGTGCATTCTGGGAACATTCAACGCTGTCGGTGAGTTTGGATGCATAATAAACCATCGACCGTTGGCTGT GCCCTGAGATTACCA

>dre-miR-181a-5-5p grp9677-889187

AACATTCAACGCTGTCGGTGAG

>dre-miR-181a-5-3p grp6903-32

ACCATCGACCGTTGGCTGTGCCt

>dre-miR-10b-1-pre 9:1943744-1943883- TGCCGTCGTCTATATATACCCTGTAGAACCGAATTTGTGTGAAAAAATAACATTCACAGATTCGATTCTAG GGGAGTATATGGTCGATGCA

>dre-miR-10b-1-5p grp6899-373724

TACCCTGTAGAACCGAATTTGT

>dre-miR-10b-1-3p grp3576-69

ACAGATTCGATTCTAGGGGAGT

>dre-miR-10b-2-pre 23:36181499-36181638+ GTCGTCTATATGTACCCTGTAGAACCGAATTTGTGTCCAAAACATCAAAATCGCAAATACGTCTCTACAGG AATACATGGGCGAC

>dre-miR-10b-2-5p grp6899-373724

TACCCTGTAGAACCGAATTTGT

>dre-miR-10b-2-3p grp9613-26

CAAATACGTCTCTACAGGAATA

>dre-miR-205-pre 23:33503901-33504040+ CTGTGCATTCTATCCTTCATTCCACCGGAGTCTGTGTAGTTGTTCAATCAGATTTCAGTGGTGTGAAGTGT AGGAAACACGG

>dre-miR-205-5p grp13814-339871

TCCTTCATTCCACCGGAGTCTG

>dre-miR-205-3p grp7602-142

GATTTCAGTGGTGTGAAGTGTA

>dre-miR-184-1-pre 18:25989745-25989884- GTCGAACACGTCTCCTTATCACTTTTCCAGCCCAGCTATCCATTTAGTATTCGTTGGACGGAGAACTGATA AGGGCATGTGCCCGAT

>dre-miR-184-1-5p grp4790-142

CCTTATCACTTTTCCAGCCCAGC

>dre-miR-184-1-3p grp12307-328479

TGGACGGAGAACTGATAAGGGC

>dre-miR-146a-pre 13:11537627-11537766+ TTGAGCACTTTTCCCTGAGAACTGAATTCCATAGATGGTGTTCATGAAAAGTTCATCTATGGGCTCAGTTC TTCTGGCAATCTGTTTAA

>dre-miR-146a-5p grp8319-288109

TGAGAACTGAATTCCATAGATGG

>dre-miR-146a-3p grp8340-355

ATCTATGGGCTCAGTTCTTCT

>dre-let-7a-1-pre 11:28380071-28380210- GACGGTGGGATGAGGTAGTAGGTTGTATAGTTTTAGGGTCACACCCACACTGGGAGATAACTATACAACCT ACTGTCTTTCTCAAAGTC

>dre-let-7a-1-5p grp6832-257492

TGAGGTAGTAGGTTGTATAGTT

>dre-let-7a-1-3p grp4135-102

CTATACAACCTACTGTCTTTCT

>dre-let-7a-2-pre 25:2130510-2130649+ CTTTGGGGTGAGGTAGTAGGTTGTATAGTTTTAGGGTGACACCCTTCCTGTTAGATAACTATACAACTTAC TGTCTTTCCTGAAG

>dre-let-7a-2-5p grp6832-257492

TGAGGTAGTAGGTTGTATAGTT

>dre-let-7a-2-3p grp11198-267

TATACAACTTACTGTCTTTCCT

>dre-let-7a-3-pre 4:17722349-17722488- CGTTTGGGGTGAGGTAGTAGGTTGTATAGTTTGAGGGTTTAACCCTTGCTGTCAGATAACTATACAACTTA CTGTCTTTCCCGAAGTG

>dre-let-7a-3-5p grp6832-257492

TGAGGTAGTAGGTTGTATAGTT

>dre-let-7a-3-3p grp11198-267

TATACAACTTACTGTCTTTCCt

>dre-let-7a-4-pre 5:31628895-31629034+ GCGATGTCTCGGGATGAGGTAGTAGGTTGTATAGTTTAGAGTTACAACACGGGAGATAACTGTACAGCCTC CTAGCTTTCCTCGAGCAGACGC

>dre-let-7a-4-5p grp6832-257492

TGAGGTAGTAGGTTGTATAGTT

>dre-let-7a-5-pre 23:5478487-5478626- CAAGGTGAGGTAGTAGGTTGTATAGTTTGGTGGGAGGGATCAAACCCTGTTCAGCTGATAACTATACAGTC TATTGCCTTCCTTG

>dre-let-7a-5-5p grp6832-257492

TGAGGTAGTAGGTTGTATAGTT

>dre-let-7a-5-3p grp12352-1011

CTATACAGTCTATTGCCTTCCT

>dre-let-7a-6-pre 6:54461637-54461776- AGGTGAGGTAGTAGGTTGTATAGTTTGTGGGAAGGATCACATCCTATTCAGGTGATAACTATACAGTCTAT TGCCTTCCCT

>dre-let-7a-6-5p grp6832-257492

TGAGGTAGTAGGTTGTATAGTT

>dre-let-7a-6-3p grp12352-1011

CTATACAGTCTATTGCCTTCCt

>dre-let-7d-1-pre 16:27120368-27120507+ GTTGCGGTGTGAGGTAGTTGGTTGTATGGTTTTGCATAATAAACAGCCCGGAGTTAACTGTACAACCTTCT AGCTTTCCCTGCGGC

>dre-let-7d-1-5p grp8315-5107

TGAGGTAGTTGGTTGTATGGTT

>dre-let-7d-1-3p grp6734-47

CTGTACAACCTTCTAGCTTTCC

>dre-let-7d-2-pre 19:10052342-10052481+ GCGCTGCAGGCTGAGGTAGTTGGTTGTATGGTTTTGCATCATAATCAGCCTGGAGTTAACTGTACAACCTT CTAGCTTTCCCTGCGGTGT

>dre-let-7d-2-5p grp8315-5107

TGAGGTAGTTGGTTGTATGGTT

>dre-let-7d-2-3p grp6734-47

CTGTACAACCTTCTAGCTTTCC

>dre-miR-26a-1-pre 9:42806525-42806664+ GGCTTTGGCCTGGTTCAAGTAATCCAGGATAGGCTTGTGATGTCCGGAAAGCCTATTCGGGATGACTTGGT TCAGGAATGAGACC

>dre-miR-26a-1-5p grp5057-221336

TTCAAGTAATCCAGGATAGGCT

>dre-miR-26a-1-3p grp7288-92

CCTATTCGGGATGACTTGGTTC

>dre-miR-26a-2-pre 2:21171542-21171681+ GGAAGTGGTTGTTCCCTTGTTCAAGTAATCCAGGATAGGCTGTCTGTCCTGGAGGCCTATTCATGATTACT TGCACTAGGTGGCAGCCGTTGCC

>dre-miR-26a-2-5p grp5057-221336

TTCAAGTAATCCAGGATAGGCT

>dre-miR-26a-2-3p grp5718-136

GGCCTATTCATGATTACTTGCA

>dre-miR-26a-3-pre 24:21079425-21079564- GTGTGTGGCTGCAACCTGGTTCAAGTAATCCAGGATAGGCTTTGTGGACTAGGGTTGGCCTGTTCTTGGTT ACTTGCACTGGGTTGCAGCTACTAAAC

>dre-miR-26a-3-5p grp5057-221336

TTCAAGTAATCCAGGATAGGCT

>dre-miR-26a-3-3p grp7505-12

CCTGTTCTTGGTTACTTGCACT

>dre-miR-22a-pre 15:24261894-24262033- GGCTGACCTGCAGCAGTTCTTCACTGGCAAGCTTTATGTCCTTGTGTACCAGCTAAAGCTGCCAGCTGAAG AACTGTTGTGGTTGGCT

>dre-miR-22a-5p grp11015-103

AGTTCTTCACTGGCAAGCTTT

>dre-miR-22a-3p grp5792-151660

AAGCTGCCAGCTGAAGAACTGT

>dre-miR-204-2-pre 7:30673512-30673651- CAGTTTGTGACCTCCTGGGTTTCCCTTTGTCATCCTATGCCTGCAGTTCCTGATGAGGCTGGGACAGCAAA GGGAGGTTCAGATGTCGACCTG

>dre-miR-204-2-5p grp6554-150213

TTCCCTTTGTCATCCTATGCCT

>dre-miR-204-3-pre 25:35406857-35406996+ (NOVEL) TGGCGTCTGTGACCTGTGGGTTTCCCTTTGTCATCCTATGCCTAGAGTTTGTGATAAGGCAGGGACAGCAA AGGGAGGCTCAGCCGTCACCACTCA

>dre-miR-204-3-5p grp6554-150213

TTCCCTTTGTCATCCTATGCCT

>dre-miR-92a-1-pre 1:2806861-2807000+ TCCCTTTCTGCGCAGGTTGGGATTGGTAGCAATGCTGTGTGTTTTGAAGGTATTGCACTTGTCCCGGCCTG TAAAGGATTGTGGA

>dre-miR-92a-1-5p grp9976-259

AGGTTGGGATTGGTAGCAATGCT

>dre-miR-92a-1-3p grp2213-133685

TATTGCACTTGTCCCGGCCTGT

>dre-miR-92a-2-pre 9:55420173-55420312- CAGCATCCCTTTCTTTGCAGGTTGGGATCGGCCGCAATGCTCTGTGCTGGAAGTATTGCACTTGTCCCGGC CTGTGAAGAGCATGGGAAATTG

>dre-miR-92a-2-5p grp1000-180

AGGTTGGGATCGGCCGCAATGCT

>dre-miR-92a-2-3p grp2213-133685

TATTGCACTTGTCCCGGCCTGT

>dre-miR-27b-pre 8:31015432-31015571-

TCTTTTCTAGCAGGTGCAGAGCTTAGCTGATTGGTGAACAGTGATTGAACTCTTTGTTCACAGTGGCTAAG TTCTGCATCTGAGGAGAGGA

>dre-miR-27b-5p grp3205-3988

AGAGCTTAGCTGATTGGTGAAC

>dre-miR-27b-3p grp237-94815

TTCACAGTGGCTAAGTTCTGC

>dre-miR-26b-pre 23:24962544-24962683- TGCATTTGGCCTTTGCCTGGTTCAAGTAATCCAGGATAGGTTAGTTCCCACTAGTACGGCCTATTCTTGGT TACTTGTTTCAGGAGGAGGCTACGAGCA

>dre-miR-26b-5p grp422-71912

TTCAAGTAATCCAGGATAGGTT

>dre-miR-26b-3p grp830-39

CCTATTCTTGGTTACTTGTTTC

>dre-miR-100-1-pre 15:20399237-20399376+ GACCTGCCTGCTGACACAAACCCGTAGATCCGAACTTGTGGTGACTGTCCACACAAGCTTGTATCTATAGG TATCTGTCTGTGTGGCCTTC

>dre-miR-100-1-5p grp5254-66492

AACCCGTAGATCCGAACTTGTG

>dre-miR-100-1-3p grp180-296

CAAGCTTGTATCTATAGGTATC

>dre-miR-100-2-pre 5:31628586-31628725+ GCAGTGATGCCAGCTGCCACAAACCCGTAGATCCGAACTTGTGGTGTCTCTGTGCACAAGCTCGTGTCTAT AGGTATGTGTCTGCGAGGTGAACGC

>dre-miR-100-2-5p grp5254-66492

AACCCGTAGATCCGAACTTGTG

>dre-miR-100-2-3p grp5031-1255

CAAGCTCGTGTCTATAGGTATG

>dre-miR-222a-pre 9:34892622-34892761- GGGTGCTCATGAGATGCTCAGTAGTCAGTGTAGATCCTGTGTCACAATCAGCAGCTACATCTGGCTACTGG GTCTCTGATGGCATTT

>dre-miR-222a-5p grp12610-24842

TGCTCAGTAGTCAGTGTAGATCC

>dre-miR-222a-3p grp5652-59743

AGCTACATCTGGCTACTGGGTCTC

>dre-miR-181b-1-pre 2:45252623-45252762- GGTCATAATCAACATTCATTGCTGTCGGTGGGTTTAGTCTTGTAACAGCTCTCTGAACAATGAATGTAACT GTGGCC

>dre-miR-181b-1-5p grp11650-57168

AACATTCATTGCTGTCGGTGGGT

>dre-miR-181b-1-3p grp3792-18

CTCACTGAACAATGAATGCAAt

>dre-miR-181b-2-pre 8:55451504-55451643- TGACTGCAATAAACATTCATTGCTGTCGGTGGGTTTCTAATAGACACAACTCACTGATCAATGAATGCAAA CTGCGGTGCA

>dre-miR-181b-2-5p grp11650-57168

AACATTCATTGCTGTCGGTGGGT

>dre-miR-181b-3-pre 22:24551748-24551887- GGTCACAATCAACATTCATTGCTGTCGGTGGGTTGGATTGTAAAAGAAAGCTCACTGAACAATGAATGCAA CTGTGTCC

>dre-miR-181b-3-5p grp11650-57168

AACATTCATTGCTGTCGGTGGGT

>dre-miR-181b-3-3p grp3792-18

CTCACTGAACAATGAATGCAAt

>dre-miR-203b-pre 20:27435474-27435613+ TCCTCTTTGGCCGAGTGGTTCTCAACAGTTCAACAGTTCTTTTGATGATTGTGAAATGTTCAGGACCACTT GATCAGACGAAGGA

>dre-miR-203b-5p grp7034-5233

AGTGGTTCTCAACAGTTCAACA

>dre-miR-203b-3p grp5500-43712

TGAAATGTTCAGGACCACTTGA

>dre-let-7e-1-pre 23:5478698-5478837- TCTTGGGGCTGAGGTAGTAGATTGAATAGTTGTGGAGCCCTGCGCTCTCTCTCTGAGATAACTATACAATC TACTGTCTTTCCTAAGG

>dre-let-7e-1-5p grp6695-48480

TGAGGTAGTAGATTGAATAGTT

>dre-let-7e-1-3p grp3803-90

CTATACAATCTACTGTCTTTCT

>dre-let-7e-2-pre 6:54462255-54462394- CCCTAAGCTGAGGTAGTAGATTGAATAGTTGTGGAGTATAAAACCTCCCTTTGAGATAACTATACAATCTA CTGTCTTTCTTTAGGG

>dre-let-7e-2-5p grp6695-48480

TGAGGTAGTAGATTGAATAGTT

>dre-let-7e-2-3p grp3803-90

CTATACAATCTACTGTCTTTCT

>dre-miR-143-1-pre 14:40131656-40131795+ ACAGTCGTCTGGCCCGCGGTGCAGTGCTGCATCTCTGGTCAACTGGGAGTCTGAGATGAAGCACTGTAGCT CGGGAGGACAACACTGT

>dre-miR-143-1-5p grp1282-94

GGTGCAGTGCTGCATCTCTGGTCt

>dre-miR-143-1-3p grp11379-30699

TGAGATGAAGCACTGTAGCTC

>dre-miR-30d-pre 16:27580668-27580807+ AACTGGTTGTTCATGCCTGTAAACATCCCCGACTGGAAGCTGTGCTACGCGGAAAACGAGCTTTCAGTTGG ATGTTTGCTGTCATCGTCCAGTT

>dre-miR-30d-5p grp2092-43821

TGTAAACATCCCCGACTGGAAGCT

>dre-miR-30d-3p grp1278-491

CTTTCAGTTGGATGTTTGCTGTt

>dre-miR-2184-1-pre 10:36859438-36859577- GAATGATGCCCTAAGCCCTAAACAGTAAGAGTTTATGTGCTGAGGTTAAAAATTCAGCACATTGTCTCTTA CTTGTAGGGAAAAGGGTTTCTATC

>dre-miR-2184-1-5p grp10995-43606

AACAGTAAGAGTTTATGTGCTG

>dre-miR-2184-1-3p grp9686-41

AGCACATTGTCTCTTACTTGTA

>dre-miR-2184-2-pre 10:36675695-36675834- GAATGATGCCCTAAGCCCTAAACAGTAAGAGTTTATGTGCTGAGGTTGAAAATTCAGCACATGGTCTCTTA CTTGTAGGGAAAAAGGTTTCTATC

>dre-miR-2184-2-5p grp10995-43606

AACAGTAAGAGTTTATGTGCTG

>dre-miR-2184-2-3p grp5452-118

AGCACATGGTCTCTTACTTGTA

>dre-miR-27c-1-pre 3:13208955-13209094+ GGTTGTGTGGTGTCAGGACTTAACCCACTTGTGAACAATGCATCGAACTTCAATGTTCACAGTGGTTAAGT TCTGCCGCCCCTAGACC

>dre-miR-27c-1-5p grp4926-2777

CAGGACTTAACCCACTTGTGAACA

>dre-miR-27c-1-3p grp2038-40890

TTCACAGTGGTTAAGTTCTG

>dre-let-7a-2-pre 15:20399468-20399607+ GTGCCCCCAGGCTGAGGTAGTAGGTTGTATAGTTTAGAATAACATCACTGGAGATAACTGTACAACCTCCT AGCTTTCCCTGAGATACAC

>dre-let-7a-2-5p grp6832-257492

TGAGGTAGTAGGTTGTATAGTT

>dre-miR-199-1-pre 20:14790227-14790366+ TCCTGCTCCGTCATCCCAGTGTTCAGACTACCTGTTCAGGATCATACTGGTGTACAGTAGTCTGCACATTG GTTAGACTGTGCATGGA

>dre-miR-199-1-5p grp7075-30211

CCCAGTGTTCAGACTACCTGTTCt

>dre-miR-199-1-3p grp11262-38570

ACAGTAGTCTGCACATTGGTT

>dre-miR-199-2-pre 5:1695931-1696070- CCCGTCCCGCCTGCCCAGTGTTCAGACTACCTGTTCAGGAATTAGTGTTTGTACAGTAGTCTGCACATTGG TTAGGCTGGATGGG

>dre-miR-199-2-5p grp7075-30211

CCCAGTGTTCAGACTACCTGTTCt

>dre-miR-199-2-3p grp11262-38570

ACAGTAGTCTGCACATTGGTT

>dre-miR-199-3-pre 3:48383360-48383499- TGCCTCCCCCTCGCCTGCCCAGTGTTCAGACTACCTGTTCATCATGCTGCAGCTGAACAGTAGTCCGCACA TTGGTTAGGCTGGGCTGGGACACA

>dre-miR-199-3-5p grp7075-30211

CCCAGTGTTCAGACTACCTGTTCt

>dre-miR-199-3-3p grp11432-5571

ACAGTAGTCCGCACATTGGTT

>dre-miR-199-4-pre 5:66977759-66977898- (NOVEL) GTGCCCGCTCTGCTTTCCCAGTGTTCAGACTACCTGTTCAGGGCGTAGAGATTGTACAGTAGTCTGCACAT TGGTTAGGCTGAGTGAGGGCTGC

>dre-miR-199-4-5p grp7075-30211

CCCAGTGTTCAGACTACCTGTTCt

>dre-miR-199-4-3p grp11262-38570

ACAGTAGTCTGCACATTGGTT

>dre-miR-10c-pre 3:24011846-24011985+ CTGTCATCTATATATACCCTGTAGATCCGGATTTGTGTAAACAGACGCACAGTCACAAATTCGTATCTAGG GGAGTATGTAGTTGATGTATAG

>dre-miR-10c-5p grp497-30749

TACCCTGTAGATCCGGATTTGT

>dre-let-7f-pre 11:28379874-28380013- CAGTGTGAGGTAGTAGATTGTATAGTTGTAGGGTAGTGATTTTATCCTGTGTAGAAGATAACTATACAATC TATTGCCTTCCCTG

>dre-let-7f-5p grp409-28668

TGAGGTAGTAGATTGTATAGTT

>dre-let-7f-3p grp4416-38

CTATACAATCTATTGCCTTCCt

>dre-miR-30e-2-pre 13:28018967-28019106+ AGCTGGCAGTACGGGCTACTGTAAACATCCTTGACTGGAAGCTGGTGCACATGATGGAGCTTTCAGTCGGA TGTTTGCAGCAGCCAACTGCTGTT

>dre-miR-30e-2-5p grp13468-24136

TGTAAACATCCTTGACTGGAAGCT

>dre-miR-30e-2-3p grp38-3744

CTTTCAGTCGGATGTTTGCAGC

>dre-miR-25-pre 14:205314-205453- GCCGGCGCTGAGAGGCGGAGACTTGGGCAGCTGCCGTCATTCCCAGAAGGCATTGCACTTGTCTCGGTCTG ACAGTGGCGGC

>dre-miR-25-5p grp10015-117

AGGCGGAGACTTGGGCAGCTGCC

>dre-miR-25-3p grp2813-23111

CATTGCACTTGTCTCGGTCTGA

>dre-miR-146b-pre 21:40390748-40390887- TGAGCTCTTGGCTTTGAGAACTGAATTCCAAGGGTGTCTGCTTTATATTCAGCCCACGGAGTTCAGTTCTT AAGTTTGGATGCTCA

>dre-miR-146b-5p grp2365-23045

TGAGAACTGAATTCCAAGGGTGT

>dre-miR-146b-3p grp2314-91

CCACGGAGTTCAGTTCTTAAGT

>dre-miR-141-pre 6:40586820-40586959- GTCTCTAGGGTACATCTTACCTGACAGTGCTTGGCTGTTCACTGATGTTCTAACACTGTCTGGTAACGATG CACTCTGGTGAC

>dre-miR-141-5p grp4691-137

CATCTTACCTGACAGTGCTTGG

>dre-miR-141-3p grp7153-22896

TAACACTGTCTGGTAACGATG

>dre-miR-182-pre 4:14140997-14141136+ GTTCTCTGATGGTATTTGGCAATGGTAGAACTCACACTGGTGAGGTAGTCAGATCCGGTGGTTCTAGACTT GCCAACTACTACCTGAGAAC

>dre-miR-182-5p grp6070-20665

TTTGGCAATGGTAGAACTCACACT

>dre-miR-182-3p grp11097-5

TGGTTCTAGACTTGCCAACT

>dre-miR-30b-pre 16:27580959-27581098+ TTCCAGTGTAGTCGCTGTAAACATCCTACACTCAGCTGTGAGCTGCAGACGAGGCTGGGCGGAGGGTGTTT GCTGTGACTGTCTGGAG

>dre-miR-30b-5p grp8125-4284

TGTAAACATCCTACACTCAGCT

>dre-miR-30b-3p grp9654-3

CTGGGCGGAGGGTGTTTGCTGT

>dre-miR-30c-pre 13:28027904-28028043+ CCATATGAGCTTCAGGGAGTGTAAACATCCTACACTCTCAGCTGGAGCGCAGCCGAGGCCGGGAGTGGGAT GTTTGCGCTCTCTGGCTCAGG

>dre-miR-30c-5p grp4783-17761

TGTAAACATCCTACACTCTCAGCT

>dre-miR-30c-3p grp1257-176

CCGGGAGTGGGATGTTTGCGCT

>dre-let-7b-1-pre 4:17721336-17721475- CAGGGTGAGGTAGTAGGTTGTGTGGTTTCAGGGTTGTGTTTTTGCCCCATCAGGAGTTAACTATACAACCT ACTGCCTTCCCTG

>dre-let-7b-1-5p grp1603-13607

TGAGGTAGTAGGTTGTGTGGTT

>dre-let-7b-1-3p grp885-564

CTATACAACCTACTGCCTTCCt

>dre-let-7b-2-pre 25:2133224-2133363+ (NOVEL) CAGGGTGAGGTAGTAGGTTGTGTGGTTTCAGGGTAGTGATTTTGCCCCATCAGGAGTTAACTATACAACCT ACTGCCTTCCCTG

>dre-let-7b-2-5p grp1603-13607

TGAGGTAGTAGGTTGTGTGGTT

>dre-let-7b-2-3p grp885-564

CTATACAACCTACTGCCTTCCt

>dre-miR-125a-1-pre 16:27137404-27137543+ GTATGTCTCTTTGTCCCTGAGACCCTTAACCTGTGAGGTCAAACTAGGTCACAGGTGAGGTCCTCAGGAAC AGGGCTGCATGC

>dre-miR-125a-2-5p grp26-12535

TCCCTGAGACCCTTAACCTGTG

>dre-miR-125a-2-pre 19:10066275-10066414+ ATCGATGTATGTCTGTGTCCCTGAGACCCTTAACCTGTGATGTCTTCCAAGGTCACAGGTGAGGTCCTTGG GAACACGGCTGTATATGAT

>dre-miR-125a-2-5p grp26-12535

TCCCTGAGACCCTTAACCTGTG

>dre-miR-125a-2-3p grp8918-10

CAGGTGAGGTCCTTGGGAACA

>dre-miR-429a-pre 23:24360688-24360827+ GGCTTGTTGATGGACGTCTTACCAGACATGGTTAGATGTAATAACTTGTGTCTAATACTGTCTGGTAATGC CGTCCATCACATGCT

>dre-miR-429a-3p grp5596-12213

TAATACTGTCTGGTAATGCCG

>dre-miR-125b-1-pre 15:20409282-20409421+ GCAAATGTGCCTCTCACAATCCCTGAGACCCTAACTTGTGACGTTTTCCTGTTATGTGCACGGGTTAGGTT CTTGGGAGCTGAGAGGGGTGCTCTGT

>dre-miR-125b-1-5p grp10313-12150

TCCCTGAGACCCTAACTTGTGA

>dre-miR-125b-1-3p grp9401-898

ACGGGTTAGGTTCTTGGGAGCT

>dre-miR-125b-2-pre 5:31637204-31637343+ GTGCCCCTCTCCTTCCCTGAGACCCTAACTTGTGACGTTCTGCTTCGATGTCCACGGGTTGGGTTCTCGGG AGCTGTGAGAGGCAC

>dre-miR-125b-2-5p grp10313-12150

TCCCTGAGACCCTAACTTGTGA

>dre-miR-125b-2-3p grp12093-406

ACGGGTTGGGTTCTCGGGAGCT

>dre-miR-125b-3-pre 10:39415828-39415967- TGCACTCCTCCTGGTCCCTGAGACCCTAACTTGTGAGCTTTGTGTGCTAAAAATCACAGGTTAAGCTCTTG GGACCTGGGCAGAGGGCA

>dre-miR-125b-3-5p grp10313-12150

TCCCTGAGACCCTAACTTGTGA

>dre-miR-125b-3-3p grp6498-288

ACAGGTTAAGCTCTTGGGACCT

>dre-miR-125c-pre 15:29150014-29150153+ GCTGCTCTCCTCCTGTTCCCTGAGACCCTAACTCGTGAGGTCTTTTTCCAAAATCACGGGTCAGGAGCTTG GGAGACAGGTGGAGGGCTTCAGC

>dre-miR-125c-5p grp7517-4196

TCCCTGAGACCCTAACTCGTGA

>dre-miR-125c-3p grp3779-19

ACGGGTCAGGAGCTTGGGAGACt

>dre-let-7g-1-pre 23:28779205-28779344+ GGGGCTGTGGAATGAGGTAGTAGTTTGTATAGTTTGGGATCACACCAGATCTGGGAGATAACTATACAGCC TACTGTCTTTCTCACAGCTGC

>dre-let-7g-1-5p grp5039-11780

TGAGGTAGTAGTTTGTATAGTT

>dre-let-7g-1-3p grp3423-103

CTATACAGTCTACTGTCTTTCt

>dre-miR-375-1-pre 6:13710646-13710785- CTGCACTTGCTTTACGTTGAGCCACACGCACAATACATGTGGATTCAGTTTTGTTCGTTCGGCTCGCGTTA AGCAAGTGCAG

>dre-miR-375-1-3p grp1291-11742

TTTGTTCGTTCGGCTCGCGTTA

>dre-miR-375-2-pre 9:11848157-11848296- GTGTTTTCTGTACTTGTCTCACGTTGAGCCACACGCACAATGCCTGCAGATGAAAGGGTTTTGTTCGTTCG GCTCGCGTTACGCAGATGCAGACAC

>dre-miR-375-2-3p grp1291-11742

TTTGTTCGTTCGGCTCGCGTTA

>dre-miR-126a-pre 8:12065947-12066086- ACTGCTTCACAGTCCATTATTACTTTTGGTACGCGCTAGGCCAGACTCAAACTCGTACCGTGAGTAATAAT GCACTGTGGCAGT

>dre-miR-126a-5p grp456-11673

CATTATTACTTTTGGTACGCG

>dre-miR-126a-3p grp6909-3281

TCGTACCGTGAGTAATAATGCA

>dre-miR-126b-pre 11:38495914-38496053+ GTCTTACCGGCCTCACGGTTCATTATTACTTTTGGTACGCGCTATGCCACTCTCAACTCGTACCGTGAGTA ATAGTGCACTGTGACTGGTGAAC

>dre-miR-126b-5p grp456-11673

CATTATTACTTTTGGTACGCG

>dre-miR-126b-3p grp6368-143

TCGTACCGTGAGTAATAGTGCA

>dre-miR-203a-pre 17:45975677-45975816+ GTCCCTCTGGTGCAGTGGTTCTTAACAGTTCAACAGTTCTATCTCAAAATTGTGAAATGTTTAGGACCACT TGACCAGAGTGAC

>dre-miR-203a-5p grp4207-250

AGTGGTTCTTAACAGTTCAACA

>dre-miR-203a-3p grp9139-10633

GTGAAATGTTTAGGACCACTTG

>dre-miR-27a-pre 2:33620174-33620313+ GTTTCGTGAGGTGCAGGACTTAGCTCACTCTGTGAACAGATCTCGGATATCCTATGTTCACAGTGGCTAAG TTCCGCTCCTCTGAGGC

>dre-miR-27a-5p grp4524-10144

AGGACTTAGCTCACTCTGTGAACA

>dre-miR-27a-3p grp4514-4242

TTCACAGTGGCTAAGTTCCGC

>dre-miR-15b-pre 7:27196719-27196858+ AGCCCTGAGTGCCCTGTAGCAGCACATCATGGTTTGTAAGTTATAAGGGCAAATTCCGAATCATGATGTGC TGTCACTGGGAGCCTGGGAGTT

>dre-miR-15b-5p grp67-9972

TAGCAGCACATCATGGTTTGTA

>dre-miR-15b-3p grp1695-495

CGAATCATGATGTGCTGTCACT

>dre-let-7c-1-pre 15:29144878-29145017+ TGTGTGCATCCAGGCTGAGGTAGTAGGTTGTATGGTTTAGAATTTTGCCCTGGGAGTTAACTGTACAACCT TCTAGCTTTCCTTGGAGCTCACA

>dre-let-7c-1-5p grp5778-9899

TGAGGTAGTAGGTTGTATGGTT

>dre-let-7c-1-3p grp6734-47

CTGTACAACCTTCTAGCTTTCC

>dre-let-7c-2-pre 10:39436594-39436733- GTGTGCATCCAGGCTGAGGTAGTAGGTTGTATGGTTTCGAATGACACCATGGGAGTTAACTGTACAACCTT CTAGCTTTCCTTGGAGTACAC

>dre-let-7c-2-5p grp5778-9899

TGAGGTAGTAGGTTGTATGGTT

>dre-let-7c-2-3p grp6734-47

CTGTACAACCTTCTAGCTTTCC

>dre-miR-101b-pre 6:31057604-31057743+ CTAGATGTGACAGGCTGCCCTGGTTCAGTTATCACAGTGCTGATGCTGTCCATCTTAAAGGTACAGTACTG TGATAACTGAAGGATGGCTGCCATCTTG

>dre-miR-101b-5p grp12422-84

TCAGTTATCACAGTGCTGATGC

>dre-miR-101b-3p grp12015-9175

TACAGTACTGTGATAACTGAAG

>dre-let-7h-pre 23:28779514-28779653+ TGAGGTAGTAAGTTGTGTTGTTGTTGGGGATCAGTATAGTATGGCCCTTGAAGGAGATAACTATACAATTT ACTGCCTTCCA

>dre-let-7h-5p grp2268-8111

TGAGGTAGTAAGTTGTGTTGTT

>dre-let-7h-3p grp11772-223

CTATACAATTTACTGCCTTCCt

>dre-miR-214-pre 20:14792413-14792552+ GAGCGTTGTCTGTCTGCCTGTCTACACTTGCTGTGCAGAACTTCCTGCACCTGTACAGCAGGCACAGACAG GCAGACAGATGGCAGCCC

>dre-miR-214-5p grp8950-712

TGCCTGTCTACACTTGCTGTGC

>dre-miR-214-3p grp129-7915

ACAGCAGGCACAGACAGGCAGt

>dre-miR-1388-pre 23:13716169-13716308+ TGGATGAGCAGTGCTTTCCAGGACTGTCCAACCTGAGAATGCTTGAGTTTTGGTCAATCTCAGGTTCGTCA GCCCATGAAAAACTGTCTCGCTCA

>dre-miR-1388-5p grp2681-7795

AGGACTGTCCAACCTGAGAATG

>dre-miR-1388-3p grp3581-1363

ATCTCAGGTTCGTCAGCCCATG

>dre-miR-221-1-pre 9:34892095-34892234- TTGTGCTGTCGTGAACCTGGCATACAATGTAGATTTCTGTGTGGTACTATCTACAGCTACATTGTCTGCTG GGTTTCAGGCCAGCAGAA

>dre-miR-221-1-5p grp4217-333

ACCTGGCATACAATGTAGATTTC

>dre-miR-221-1-3p grp4811-7549

AGCTACATTGTCTGCTGGGTTT

>dre-miR-221-2-pre 6:59627852-59627991- (NOVEL) GGACGCGGCTCTGGCCCTGGCACACAGTGTATTGGTCTGTGTGTGTGTGTGTCTACAGCTACATTGTCTGC TGGGTTTCAGAGCCGCTTC

>dre-miR-221-2-3p grp4811-7549

AGCTACATTGTCTGCTGGGTTT

>dre-miR-16c-pre 9:30685641-30685780- GTGTGTTGTCTTGCTTTAGCAGCATGTAAATATTGGAGTTACTCCTTGGCCAATGCCTCCAATATTGCTCG TGCTGCTGAAGCAAGAAGTCACC

>dre-miR-16c-5p grp2055-3930

TAGCAGCATGTAAATATTGGAGT

>dre-miR-16c-3p grp1479-7419

TCCAATATTGCTCGTGCTGCTGt

>dre-miR-23a-1-pre 22:4997587-4997726+ GTGCGGCTGTGGCGGGGAGGGTTCCTGGCACCGTGATTTGGTGGATAAACAGAAATGAAAATCACATTGCC AGGGATTTCCACTCCTGCAC

>dre-miR-23a-1-3p grp12800-7080

ATCACATTGCCAGGGATTTCC

>dre-miR-23a-3-pre 2:33618297-33618436+ GGACCAGCTGGAGGGATTCCTGGCAGAGTGATTTGGGATTATATCATAAAATCACATTGCCAGGGATTTCC AACCAGCTGTGAAC

>dre-miR-23a-3-3p grp12800-7080

ATCACATTGCCAGGGATTTCC

>dre-miR-23a-4-pre 3:13304680-13304819- GCCGGCCAGGGGAATTCCTGGCAGAGTGATTTTTAAACCTAATGACTGAATCACATTGCCAGGGATTTCCA ATGGCTCGTGT

>dre-miR-23a-4-3p grp12800-7080

ATCACATTGCCAGGGATTTCC

>dre-miR-338-1-pre 12:38062309-38062448+ GGTTTCTCCCTGCAACAATCTCCTGATGCTGCCTGAGTGTTTTTCTTCCACTCCAGCATCAGTGATTTTGT TGCCGGAGGTCACC

>dre-miR-338-1-5p grp2554-23

AACAATCTCCTGATGCTGCCTGAGT

>dre-miR-338-1-3p grp8564-6698

TCCAGCATCAGTGATTTTGTT

>dre-miR-338-2-pre 12:18795087-18795226+ TCTGGTGCCTGCTGAGAACAATATCCTGATGCTGAATGAGTGTGTTGAAGGAAACTCCAGCATCAGTGATT TTGTTGCCAGAGGAGCACTTTGG

>dre-miR-338-2-5p grp1242-31

AACAATATCCTGATGCTGAATGAGT

>dre-miR-338-2-3p grp8564-6698

TCCAGCATCAGTGATTTTGTT

>dre-miR-338-3-pre 3:52527361-52527500- (NOVEL) GGCTCTGGTGTCTCCTGGCAACAATATCCTGGTGCTGCCTGAGTACATCTCACAGACTCCAGCATCAGTGA TTTTGTTGCCGGGGGAAAACCCC

>dre-miR-338-3-5p grp7006-24

AACAATATCCTGGTGCTGCCTGAGT

>dre-miR-338-3-3p grp8564-6698

TCCAGCATCAGTGATTTTGTT

>dre-miR-142a-pre 5:3156766-3156905+ GGACGTACAGTGCAGTCATCCATAAAGTAGAAAGCACTACTAAACCCCTCGCCACAGTGTAGTGTTTCCTA CTTTATGGATGAGTGTACTGTTGGCT

>dre-miR-142a-5p grp669-6556

CATAAAGTAGAAAGCACTACT

>dre-miR-142a-3p grp8453-486

AGTGTTTCCTACTTTATGGATG

>dre-miR-92b-pre 16:45736475-45736614+ TGTAATCCTACGGGCAGGGAGGTGTGGGATGTTGTGCAGTGTTGTTCAATCTCCCGCCAATATTGCACTCG TCCCGGCCTCCCTGACCACGAGGAACA

>dre-miR-92b-3p grp878-6416

TATTGCACTCGTCCCGGCCTCC

>dre-miR-183-pre 4:14139911-14140050+ CTCCTGTTCTGTGTATGGCACTGGTAGAATTCACTGTGAAAGCACACTATCAGTGAATTACCAAAGGGCCA TAAACAGAGCAGAG

>dre-miR-183-5p grp10435-6387

TATGGCACTGGTAGAATTCACT

>dre-miR-200a-pre 23:24359034-24359173+ GGCACTTAGCAGCCATCTTACCGGACAGTGCTGGACTGTATAACTGTTTTCTAACACTGTCTGGTAACGAT GTTTGTTGGGTGACC

>dre-miR-200a-5p grp879-1576

CATCTTACCGGACAGTGCTGGA

>dre-miR-200a-3p grp7153-22896

TAACACTGTCTGGTAACGATG

>dre-miR_200b-pre 23:24358899-24359038+ GGTAGTCGTCTCCATCTTACGAGGCAGCATTGGATTTCATTACTTTTTCTAATACTGCCTGGTAATGATGA TGATTGCTGCC

>dre-miR-200b-5p grp487-33

CATCTTACGAGGCAGCATTGGA

>dre-miR-200b-3p grp4559-6313

TAATACTGCCTGGTAATGATGc

>dre-miR-200c-pre 6:40587023-40587162- CAATGGATGCCTGGCTCCATCTTACAAGGCAGTTTTGGATGTTATATCTTCTCTAATACTGCCTGGTAATG ATGCAGATGGTCATCTAGAG

>dre-miR-200c-5p grp9034-360

CATCTTACAAGGCAGTTTTGGA

>dre-miR-200c-3p grp4559-6313

TAATACTGCCTGGTAATGATGC

>dre-miR-99-1-pre 15:29144629-29144768+ GCCACTTGTCATTAACCCGTAGATCCGATCTTGTGATAAGTTTGATGGCACAAGCTCGATTCTATGGGTCT CTGTCTCTGTGGT

>dre-miR-99-1-5p grp13110-6097

AACCCGTAGATCCGATCTTGTG

>dre-miR-99-1-3p grp2102-13

CAAGCTCGATTCTATGGGTCTCT

>dre-miR-99-2-pre 10:39438820-39438959- GCTTTGACCACTTGTCACAAACCCGTAGATCCGATCTTGTGGCGTAATCGGCAACCCAAGCTCGATTCTGT GGGTCTCTGTCACTGTGGTGAACTC

>dre-miR-99-2-5p grp13110-6097

AACCCGTAGATCCGATCTTGTG

>dre-miR-99-2-3p grp13599-166

CAAGCTCGATTCTGTGGGTCT

>dre-miR-152-pre 3:24387190-24387329+ CTGTTCACCTGGCTCAAGTTCTGTGATACACTCAGACTTTGAATCAGTGGTAGTCAGTGCATGACAGAACT TTGGCCCGGACGG

>dre-miR-152-5p grp9519-3591

AAGTTCTGTGATACACTCAGACT

>dre-miR-152-3p grp12231-5676

TCAGTGCATGACAGAACTTTGt

>dre-miR-16b-pre 1:47219508-47219647- GACTGGCTGCCTGGCTGTAGCAGCACGTAAATATTGGAGTCAAAGCACTTGCGAATCCTCCAGTATTGACC GTGCTGCTGGAGTTAGGCGGGCCGTT

>dre-miR-16b-5p grp4945-5356

TAGCAGCACGTAAATATTGGAGT

>dre-miR-16b-3p grp7776-8

CAGTATTGACCGTGCTGCTGGA

>dre-miR-148-pre 9:1633981-1634120- AGCTCTCTGGCTTTCCAAGTAAAGTTCTGTGATACACTCCGACTCTGAATGTTTGCAGTCAGTGCATTACA GAACTTTGTTTTGGGAGTTTAAAGCT

>dre-miR-148-5p grp10040-3711

AAGTTCTGTGATACACTCCGACT

>dre-miR-148-3p grp6766-5131

TCAGTGCATTACAGAACTTTGT

>dre-miR-454b-pre 5:14498655-14498794+ TCTTGCAGGCGAGACCCTATCAATATTGCCTCTGCTTTTCTCACTGTTTATGGAGTAGTGCAATATTGCTT ATAGGGTCTTGACTTTAAGG

>dre-miR-454b-5p grp1417-10

CCCTATCAATATTGCCTCTGCT

>dre-miR-454b-3p grp11024-4503

TAGTGCAATATTGCTTATAGGGTC

>dre-miR-196a-1-pre 23:36139594-36139733+ GCGCGGCTGGTGCGTGGTTTAGGTAGTTTCATGTTGTTGGGATTGGCTTCCTGGCTCGACAACAAGAAACT GCCTTGATTACGTCAGTTCGT

>dre-miR-196a-1-5p grp7025-3693

TAGGTAGTTTCATGTTGTTGGG

>dre-miR_196a-1-3p grp2688-950

CGACAACAAGAAACTGCCTTGA

>dre-miR-196a-2-pre 19:19213313-19213452+ GAACCAGACTGTCGAGTGGTTTAGGTAGTTTCATGTTGTTGGGATTACATTCAAACTCTGCAACGTGAAAC TGTCTTAATTGCCCCAGTTTCATTC

>dre-miR-196a-2-5p grp7025-3693

TAGGTAGTTTCATGTTGTTGGG

>dre-miR-196a-2-3p grp13452-147

CTGCAACGTGAAACTGTCTTAA

>dre-miR-128-3-pre Zv9_NA973:5578-5717- (NOVEL) TGTGTGTCGGAGCGGCTGAATGCGGGGCCGTGGCGCTGTCTGAGACGCTCTACTATTCTCACAGTGAACCG GTCTCTTTTCCAGCCGCTCACACTCG

>dre-miR-128-3-3p grp2325-3564

TCACAGTGAACCGGTCTCTTT

>dre-miR-128-1-pre 22:12504145-12504284+ TGGAGGAGGAGTGCTGGGAGACGGGGCCGTGGCACTGTATGAGATTCATGTAGGCTTTCTCACAGTGAACC GGTCTCTTTTTCCAGCCCTCACTGACA

>dre-miR-128-1-5p grp10647-129

CGGGGCCGTGGCACTGTATGAGA

>dre-miR-128-1-3p grp2325-3564

TCACAGTGAACCGGTCTCTTT

>dre-miR-128-2-pre 19:44518222-44518361+ GGGTCTGTCAGTAGTAGGACAGGGGGCCGTTTCTACTGTCAGAGATGCTGCCACTCGTCTCACAGTGAACC GGTCTCTTTTCCTGCTATTCACTTCT

>dre-miR-128-2-3p grp2325-3564

TCACAGTGAACCGGTCTCTTT

>dre-miR-31-pre 7:76268782-76268921+ TGGAAGAGAAGAGATGGCAAGATGTTGGCATAGCTGTTAATGTTTATGGGCCTGCTATGCCTCCATATTGC CATTTCTGCACTTCCA

>dre-miR-31-5p grp10983-3372

TGGCAAGATGTTGGCATAGCTG

>dre-miR-31-3p grp10834-279

TGCTATGCCTCCATATTGCCATT

>dre-miR-27e-pre 22:5004334-5004473+ TCGCTCACGGCGCAGAGCTTAGCTAATCGGTGAGCATTGATCCCTTAAGAAAACTGTTCACAGTGGCTAAG TTCAGTGTCTGGAGTGA

>dre-miR-27e-5p grp8442-91

AGAGCTTAGCTAATCGGTGAGC

>dre-miR-27e-3p grp3960-3368

TTCACAGTGGCTAAGTTCAGT

>dre-miR-140-pre 25:35855072-35855211+ TGTCTCCTGTGTCCCGTCAGTGGTTTTACCCTATGGTAGGTTACGTCATGCTGTTCTACCACAGGGTAGAA CCACGGACGGGATGTCTGGAGGTG

>dre-miR-140-5p grp391-100

CAGTGGTTTTACCCTATGGTAGt

>dre-miR-140-3p grp9767-3235

ACCACAGGGTAGAACCACGGACt

>dre-miR-181c-pre 3:34282938-34283077+ GCTCTGGGTCCTGATTCACATTCATTGCTGTCGGTGGGTTTTATCTCTTCGACTCGCCGGACAATGAATGA GAACTACGGCTCCTCCTGC

>dre-miR-181c-5p grp2236-3214

CACATTCATTGCTGTCGGTGGGTT

>dre-miR-181c-3p grp10632-23

CTCGCCGGACAATGAATGAGAA

>dre-miR-148-2-pre 6:10815386-10815525+ (NOVEL) GGCTCCTCGGGGAAAGTTCTGTGGTCCACTCTGGCTGTGTGAGTGTGTGGTCAGTGCATTACAGAACTTTG CTTTAGAAGCT

>dre-miR-148-2-5p grp4045-1771

AAGTTCTGTGGTCCACTCTGGCT

>dre-miR-148-2-3p grp6766-513

TCAGTGCATTACAGAACTTTGt

>dre-miR-15c-pre 15:1538844-1538983+ CCTTAGACCGCTAAAGCAGCGCGTCATGGTTTTCAACATTAGAGAAGGTGCAAGCCATCATTTGCTGCTCT AGAGTTTTAAGG

>dre-miR-15c-5p grp4853-302

AAGCAGCGCGTCATGGTTTTCA

>dre-miR-15c-3p grp7648-8

CAAGCCATCATTTGCTGCTCTA

>dre-miR-19b-pre 1:2806743-2806882+ TGCTGGACCCCCGGTCAGTTTTGCTGGTTTGCATTCAGCTTTTAAGACTGTGCGCTGTGCAAATCCATGCA AAACTGATTGTGGCAGCA

>dre-miR-19b-3p grp9643-2971

TGTGCAAATCCATGCAAAACTG

>dre-miR-19c-pre 14:32336867-32337006- GCCCTGTTATCTGGGGTGAGTTTTGCAGGATTGCATCCGGCTTTATTACAACATGCTGTGCAAATCCATGC AAAACTCGCTGCGCCAGGGAC

>dre-miR-19c-3p grp3312-18

TGTGCAAATCCATGCAAAACTCG

>dre-miR-27d-pre 10:15983297-15983436+ TTCTGAGCGGGTGCAGAGCTTGGCTGATTGGTGAACGTGCATGGCTTGTGTTTTTGTTCACAGTGGCTAAG TTCTTCACCCGAAAAGAA

>dre-miR-27d-5p grp7408-342

CAGAGCTTGGCTGATTGGTGAAC

>dre-miR-27d-3p grp3639-67077

TTCACAGTGGCTAAGTTCTg

>dre-miR-460-pre 2:2103416-2103555- TGCTGTGGACTCCTCGGCTCCTGCATTGTACACACTGTGCGGAAAACATGGACATGCACAGCGCATACAAT GTGGATGCTGTGGAGCCCAGACA

>dre-miR-460-5p grp8587-421

CCTGCATTGTACACACTGTGC

>dre-miR-460-3p grp4879-2744

CACAGCGCATACAATGTGGATG

>dre-miR-23b-pre 10:15983140-15983279+ TGTTGTGGCTGTGTGGGTTCTTGGCATGCTGATTTGTGACTGTAGTAAAAAAAAATCACATTGCCAGGGAT TACCACACTACCACGGCA

>dre-miR-23b-3p grp5274-2648

ATCACATTGCCAGGGATTACCACt

>dre-miR-23b-2-pre 8:31015621-31015760- (NOVEL) CTGCTGGCTGTGTGGGTTCCTGGCGTGCTGATTTGTGACTTAAGATAAAAATCACATTGCCAGGGATTACC ACACAACCGCCACCCGG

>dre-miR-23b-2-3p grp5274-2648

ATCACATTGCCAGGGATTACCACt

>dre-miR-458-pre 14:26237136-26237275- GTGTGGTGCAGATAGCAGCGCCATTTACAGAGCTATAAGCATCATAGTTGTCATAGCTCTTTGAATGGTAC TGCCATATGCACTGAAC

>dre-miR-458-3p grp6805-2455

ATAGCTCTTTGAATGGTACTGC

>dre-miR-22b-pre 5:13813137-13813276+

GGCTGGCGTTACTTCACAGTCGTTCTTCACTGGCTAGCTTTATGTGGCAGCACCTTAAAGCTGCCAGTTGA AGAGCTGTTGTGGGTAACCTTAAC

>dre-miR-22b-3p grp1011-2442

AAGCTGCCAGTTGAAGAGCTGT

>dre-miR-139-pre 15:47153773-47153912+ TAGTTTGGTCTGGCTGTATTCTACAGTGCATGTGTCTCCAGTGTTTCTATGGCGACTGGGGAGGCAGCGCT GTTGGAATAACAACCAGAACCGCTG

>dre-miR-139-5p grp12872-2363

TCTACAGTGCATGTGTCTCCAGT

>dre-miR-142b-pre 15:14402905-14403044- GAGGTACAGTGCAGTCACTCATAAAGTAGACAGCACTACTAAACTTCTCTACACAGTGTAGTGTTTCCTAC TTTATGGATGAGTGTACTGTTGGCGGC

>dre-miR-142b-5p grp10620-2309

CATAAAGTAGACAGCACTACT

>dre-miR-142b-3p grp8453-486

AGTGTTTCCTACTTTATGGATG

>dre-miR-24-6-pre 2:33623629-33623768+ (NOVEL) GGGTTCAACCTCCTGTGCCTACTGAGCTGATAACAGTTGATGTATTTAGCACTGGCTCAGTTCAGCAGGAA CCGGAGTTAAGCCC

>dre-miR-24-6-5p grp3370-769

TGCCTACTGAGCTGATAACAGT

>dre-miR-24-6-3p grp12832-2176

TGGCTCAGTTCAGCAGGAAC

>dre-miR-24-3-pre 8:31010517-31010656- TGGAGCTGGCCTCCCGTGCCTACTGAGCTGATTACAGTACTGTGCAAACACTGGCTCAGTTCAGCAGGAAC AGGAGTCTGGTCCA

>dre-miR-24-3-5p grp1919-184

TGCCTACTGAGCTGATTACAGT

>dre-miR-24-3-3p grp12832-2176

TGGCTCAGTTCAGCAGGAA

>dre-miR-24-5-pre 3:13298247-13298386- GTGGGCTGGTCTCCTGTGCCTGCTGTGCTGATAATCAGTGGACGGCTGTGACTGGCTCAGTTCAGCAGGAA CAGGGGCCTGGTCTTC

>dre-miR-24-5-5p grp4705-30

TGCCTGCTGTGCTGATAATCA

>dre-miR-24-5-3p grp12832-2176

TGGCTCAGTTCAGCAGGAAC

>dre-miR-24-4-pre 22:5011860-5011999+ GACTGAGGGCTGTTCCCACTTGTGCCTGCTAAACTGGTATCAGTATGTTGATTTAGTGCTGGCTCAGTTCA GCAGGAACAGGTGTGAAGTCCTCTACTC

>dre-miR-24-4-3p grp12832-2176

TGGCTCAGTTCAGCAGGAAC

>dre-miR-193b-pre 3:42895093-42895232- GTGATTTCAGTGACGGGACTTTGGGGGCGAGATGAGTATTGATCTCTATCCAACTGGCCCGCAAAGTCCCG CTTCTGGGACTCAC

>dre-miR-193b-5p grp10390-758

CGGGACTTTGGGGGCGAGATG

>dre-miR-193b-3p grp12804-2174

AACTGGCCCGCAAAGTCCCGCT

>dre-miR-455-1-pre 5:67759742-67759881-

GATCCCTGGTGTGAGGGTATGTGCCCTTGGACTACATCGTGGAAGCCAGCACCATGCAGTCCATGGGCATA TACACTTGCCTCAAGGCCTAGATC

>dre-miR-455-1-5p grp567-2171

TATGTGCCCTTGGACTACATCG

>dre-miR-455-1-3p grp13453-250

GCAGTCCATGGGCATATACACT

>dre-miR-103-2-pre 14:47049004-47049143+ (NOVEL) CTCTTGGCTTTGAGCCTCTTTACAATGCTGCCTTGTAGACCCAGAATCAAGCAGCATTGTACAGGGCTATG AAAGCACAGAG

>dre-miR-103-2-3p grp11902-2033

AGCAGCATTGTACAGGGCTATc

>dre-miR-103-pre 13:15177262-15177401+ GCCCTGGTCTGTCAGCCTCTTTACGGTGCTGCCTTGTGGAATCTGGATCAAGCAGCATTGTACAGGGCTAT GAGAGACCCGGGC

>dre-miR-103-3p grp11902-2033

AGCAGCATTGTACAGGGCTATc

>dre-miR-107a-pre 12:18027634-18027773+ TCTGTGTGCTCTGAGCTTCTTTACAGTGTTGTCTTGTGGCATGGAGATCAAGCAGCATTGTACAGGGCTAT CACAGCACACTGA

>dre-miR-107a-5p grp1157-206

AGCTTCTTTACAGTGTTGTCTTG

>dre-miR-107a-3p grp11902-2033

AGCAGCATTGTACAGGGCTATC

>dre-miR-130b-pre 5:14501628-14501767+ GTCTGTTGCCTGACACTCTTTCCCTGTTGCACTACTGTGGGAGCTGCAGCAAAGCAGTGCAATAATGAAAG GGCATCAGTCCACTGGAC

>dre-miR-130b-5p grp13715-1912

ACTCTTTCCCTGTTGCACTACT

>dre-miR-130b-3p grp141-1722

CAGTGCAATAATGAAAGGGCATt

>dre-miR-10a-pre 12:28705856-28705995+ TGATGTCTGTCATCTATATATACCCTGTAGATCCGAATTTGTGTGAATATACAGTCGCAAATTCGTGTCTT GGGGAATATGTAGTTGACATAAACACA

>dre-miR-10a-5p grp1421-1753

TACCCTGTAGATCCGAATTTGT

>dre-miR-301a-pre 10:34405132-34405271- CAAGTGCTGTTAACAGGTGCTCTGACTTCATTGCACTACTGTATTGGACAGCTAGCAGTGCAATAGTATTG TCAAAGCGTCTGAGAGCAGCTTTG

>dre-miR-301a-3p grp9137-1746

CAGTGCAATAGTATTGTCAAAGC

>dre-miR-130c-2-pre 5:14491163-14491302+ CTGTTGTTGTTGACCAGGGCCCTTTTTCTGTTGTACTACTGTGCAGTCAGATGAGCAGTGCAATATTAAAA GGGCATTGGCTGACAAAAAAAG

>dre-miR-130c-2-5p grp8217-180

GCCCTTTTTCTGTTGTACTAC

>dre-miR-130c-2-3p grp2237-1702

CAGTGCAATATTAAAAGGGCA

>dre-miR-130c-1-pre 10:17483602-17483741+ ACCATGTTTTGTCCATTGCCCTTTTTCTGTTGTACTACTGGCCAATCAGAAGAGCAGTGCAATATTAAAAG GGCATTGGCTGATAGAACAGAGT

>dre-miR-130c-1-5p grp8217-180

GCCCTTTTTCTGTTGTACTAC

>dre-miR-130c-1-3p grp2237-1702

CAGTGCAATATTAAAAGGGCA

>dre-miR-222b-pre 21:44433859-44433998+ TGACGTGATCTGCAGACTCAGTACTCGGTGTAGAGTCTGTGTGATTCAGAAGAGCAGCTACATCTGAATAC TGGGTCAGTGGAGACGTCA

>dre-miR-222b-5p grp7788-88

ACTCAGTACTCGGTGTAGAGTC

>dre-miR-222b-3p grp3723-1575

AGCTACATCTGAATACTGGGTC

>dre-miR-192-pre 10:27698801-27698940+ TAGGACACAGGGTGATGACCTATGAATTGACAGCCAGTGTTTGCAGTCCAGCTGCCTGTCAGTTCTGTAGG CCACTGCCCTGTTTATCCTA

>dre-miR-192-5p grp4190-1480

ATGACCTATGAATTGACAGCCA

>dre-miR-456-pre 17:22657070-22657209+ GCTGGTTGTGTGTGCAGGCATCTTTCCAGTCTACATGTGGATCCAGGAGTCTGCAGGCTGGTTAGATGGTT GTCACGTACCCAGC

>dre-miR-456-3p grp9652-134

CAGGCTGGTTAGATGGTTGTCt

>dre-miR-29a-pre 4:10671248-10671387- TCCCCACCAAACGATGACTGATTTCCTTTGGTGCTTAGAGTCCCATCTGTCATCTAGCACCATTTGAAATC GGTTATAATGACTGGGGA

>dre-miR-29a-5p grp8851-104

TGACTGATTTCCTTTGGTGCTT

>dre-miR-29a-3p grp9484-1303

TAGCACCATTTGAAATCGGTTA

>dre-miR-24-1-pre 10:15989395-15989534+ GGACCTGAGCTCCGGTGCCTTCTGAGCTGATATCAGTTGTAGTAAATCACTGGCTCAGTTCAGCAGGAACA GGAGTGTGGCC

>dre-miR-24-1-3p grp12832-2176

TGGCTCAGTTCAGCAGGAAC

>dre-miR-365-1-pre 3:42892962-42893101- GCAGGGAGAATGAGGGGCTTTTGGGGGCACTTGTGTTTCAGTTTCACCATCATAATGCCCCTAAAAATCCT TATTGCTCTTGC

>dre-miR-365-1-3p grp4946-1276

TAATGCCCCTAAAAATCCTTAT

>dre-miR-365-2-pre 3:44211648-44211787+ GCAAGAAAAATGAGGGACTTTTAGGGGCAGCTGTGTTTTATTAACCCAGTCATAATGCCCCTAAAAATCCT TATTGCTCTTGC

>dre-miR-365-2-5p grp13188-13

AGGGACTTTTAGGGGCAGCTGTG

>dre-miR-365-2-3p grp4946-1276

TAATGCCCCTAAAAATCCTTAT

>dre-miR-365-3-pre 6:19393980-19394119+ GCAAGAAAAGTGAGGGACTTTTAGGGGCAGCTGTGTTATACTGACCCAGTCATAATGCCCCTAAAAATCCT TATTGCTCTTGC

>dre-miR-365-3-5p grp13188-13

AGGGACTTTTAGGGGCAGCTGTG

>dre-miR-365-3-3p grp4946-1276

TAATGCCCCTAAAAATCCTTAT

>dre-miR-196b-pre 3:23964566-23964705+ AGCTGGTCTGTGATTTAGGTAGTTTCAAGTTGTTGGGCTGGACGTTTAATTTCACAGGAACCTGAAACTGC CTGAATTGCTCCAGTT

>dre-miR-196b-5p grp12773-1252

TAGGTAGTTTCAAGTTGTTGGGC

>dre-miR-196b-3p grp6995-51

ACAGGAACCTGAAACTGCCT

>dre-miR-155-pre 1:451089-451228+ CTCCTGGTGCAGGTTTAATGCTAATCGTGATAGGGGTTTAGTGCTGATGAACACCTATGCTGTTAGCATTA ATCTTGCGCTAGTGAG

>dre-miR-155-5p grp11819-1231

TTAATGCTAATCGTGATAGGGGT

>dre-miR-457a-pre 7:27197243-27197382+ GTGTGCCTGACAGAAGCAGCACATCAATATTGGCAGCTGCCCTCTCTCTGGGTTGCCAGTATGGTTTGTGC TGCTCCCGTCAGACAGAC

>dre-miR-457a-5p grp9332-1143

AAGCAGCACATCAATATTGGCA

>dre-miR-457a-3p grp13829-53

CCAGTATGGTTTGTGCTGCTCCC

>dre-miR-724-pre 11:3156990-3157129+ CAGCAGACTGGATTTAAAGGGAATTTGCGACTGTTAGTCAGATTTGTAGAACAGCCACACCTTCCTTTTAA GATCTTGCCTGCT

>dre-miR-724-5p grp4562-1087

TTAAAGGGAATTTGCGACTGTT

>dre-miR-724-1-pre 8:24917105-24917244+ (NOVEL) CAGCAGACTGGATTTAAAGGGAATTTGCGACTGTTTAGTCACATTATTAGAACAGCCACACCTTCCTTTTA AGATCTTGCCTGCTG

>dre-miR-724-1-5p grp4562-1087

TTAAAGGGAATTTGCGACTGTT

>dre-miR-196d-pre 16:22997788-22997927+ ACTGCTAAGTGATTTAGGTAGTTTTATGTTGTTGGGCTCTATTTTATATCCCCGCAACACGAAACTGTCTT AATTGCCTCGCAGT

>dre-miR-196d-5p grp11987-964

TAGGTAGTTTTATGTTGTTGGGt

>dre-miR-196c-pre 11:2162524-2162663- GTCCAGCTGATGCGTGGTTTAGGTAGTTTGATGTTGTTGGGGTTGACTTCCTGGCTCGACAACAAGAAACT GCCTTGATTACGTCAGTTTGCC

>dre-miR-196c-5p grp13526-93

TAGGTAGTTTGATGTTGTTGGGt

>dre-miR-196c-3p grp2688-950

CGACAACAAGAAACTGCCTTGA

>dre-miR-16a-pre 15:1539014-1539153+ CCTTCCTCGCTTTAGCAGCACGTAAATATTGGTGTGTTATAGTCAAGGCCAACCCCAATATTATGTGTGCT GCTTCAGTAAGGCAGG

>dre-miR-16a-5p grp6244-922

TAGCAGCACGTAAATATTGGTG

>dre-miR-20a-2-pre 9:55420459-55420598- (NOVEL)

GCTCTGATTCCAGCAGTATTAAAGTGCTTATAGTGCAGGTAGTTTGTGAAAATCCTGATCTACTGCAATGT GAGCACTTCAAGTACTTCTAGATGC

>dre-miR-20a-2-5p grp8336-909

TAAAGTGCTTATAGTGCAGGTAG

>dre-miR-20a-pre 1:2806553-2806692+ GTGTTAGAGTTTCAGCAGTGCTAAAGTGCTTATAGTGCAGGTAGTATTTCTGTCATCTACTGCAGTGTGAG CACTTGAAGTACTTCTAGCTTAATGC

>dre-miR-20a-5p grp8336-909

TAAAGTGCTTATAGTGCAGGTAG

>dre-miR-20a-3p grp396-62

ACTGCAGTGTGAGCACTTGAAGT

>dre-miR-132-pre 10:36666485-36666624+ CTGTCTCCATGCTGACCGTGGCTTTAGATTGTTACTGTAGCGTTGGCACGTGGTAACAGTCTACAGCCATG GTCGCCAAGGGGCAG

>dre-miR-132-5p grp6716-858

ACCGTGGCTTTAGATTGTTACT

>dre-miR-132-3p grp1607-159

TAACAGTCTACAGCCATGGTCG

>dre-miR-17a-1-pre 1:2806049-2806188+ GTGTCAATGTATTGTCAAAGTGCTTACAGTGCAGGTAGTATTATGGAATATCTACTGCAGTGGAGGCACTT CTAGCAATACACTTGACC

>dre-miR-17a-1-5p grp1621-842

CAAAGTGCTTACAGTGCAGGTAG

>dre-miR-17a-2-pre 9:55420866-55421005- GTCACTGTAGTGTCAAAGTGCTTACAGTGCAGGTAGTTCAATATAATCTACTGCAGTGGAGGCACTTCAAG CTTTACCGTGAC

>dre-miR-17a-2-5p grp1621-842

CAAAGTGCTTACAGTGCAGGTAG

>dre-miR-145-pre 14:40133337-40133476+ TCATTTCCTCATCCCCGGGGTCCAGTTTTCCCAGGAATCCCTTGGGCAATCGAAAGGGGGATTCCTGGAAA TACTGTTCTTGGGGTTGGGGGTGGA

>dre-miR-145-5p grp7945-803

GTCCAGTTTTCCCAGGAATCCCT

>dre-miR-145-3p grp11405-296

ATTCCTGGAAATACTGTTCTT

>dre-miR-731-pre 8:26935431-26935570+ TGATGCTGATCTGGAATGACACGTTTTCTCCCGGATCGCCAGAAATATGTTTCGCCACCGGGAACTTCGTG TCAGCCAAGATTGGCATCA

>dre-miR-731-5p grp9254-109911

AATGACACGTTTTCTCCCGGATCG

>dre-miR-731-3p grp8923-688

CACCGGGAACTTCGTGTCAGCC

>dre-miR-454a-pre 10:34404961-34405100- CCTAATTCTTGGGACCCTATCAGTATTGCCTCTGCTGTCCACTGTGTTCAGAGTAGTGCAATATTGCTAAT AGGGTTTTAGGTTTTAGG

>dre-miR-454a-5p grp4175-15

ACCCTATCAGTATTGCCTCTGCT

>dre-miR-454a-3p grp1916-709

TAGTGCAATATTGCTAATAGGGT

>dre-miR-218a-1-pre 14:25562458-25562597-

TGGTGCAGCTGTCTCTTGTGCTTGATCTAACCATGTGCCGCCGCCTACACAAGCCTCACATGGTTCCGTCA AGCACCAGGGACCGCTGGGCACA

>dre-miR-218a-1-5p grp9287-619

TTGTGCTTGATCTAACCATGTG

>dre-miR-218a-1-3p grp11413-143

TCACATGGTTCCGTCAAGCACC

>dre-miR-218a-2-pre 1:22640924-22641063+ GCGGGGTTTTCCTTTGTGCTTGATCTAACCATGTGGTTGCAGACTCAGACTAATACATGGTTCTGTCAAGC ACCATGGAAGGTCTTGC

>dre-miR-218a-2-5p grp9287-619

TTGTGCTTGATCTAACCATGTG

>dre-miR-218a-2-3p grp9003-46

ATACATGGTTCTGTCAAGCACC

>dre-miR-93-pre 14:205620-205759- GTGGCTGTGTGTGTTAAAAGTGCTGTTTGTGCAGGTAGTGTGTTTCCTCTACTGTAGGAGCAGCACTTCAC AACACACACACTGCTGC

>dre-miR-93-5p grp394-595

AAAAGTGCTGTTTGTGCAGGTAG

>dre-miR-150-pre 3:32708234-32708373+ GTCCATCTCCGTCTCTCCCAATCCTTGTACCAGTGTCTGATTTACAGATGACGCTGGACGGGGTTTGGGGG GGGCTGAGGGAGGAC

>dre-miR-150-5p grp12186-532

TCTCCCAATCCTTGTACCAGTG

>dre-let-7j 6:41385599-41385738+ GGGTTGAGGTAGTTGTTTGTACAGTTTTTAGGGTCTGTTATTCTGCCCTGTTAAGGAGCTAACTGTACAGA CTACTGCCTTGCCC

>dre-let-7j-5p grp12629-8982

TGAGGTAGTTGTTTGTACAGTT

>dre-let-7j-3p grp7022-1136

CTGTACAGACTACTGCCTTGCt

>dre-miR-19d-pre 14:205419-205558- GTTGTGTGGCGGTCAGCTTTGCGGGGTGGGCAGTCAGCCTCCGTGTGGCCGCTGTGCAAACCCATGCAAAA CTGAGCGCTGCGCTAC

>dre-miR-19d-3p grp493-492

TGTGCAAACCCATGCAAAACTG

>dre-miR-455-pre 16:55426098-55426237+ TGTTCCCTGAAGTGAGGGTATGTGCCCTTGGACTACATTGTGGAGGCCAGCACCATGCAGTCCATGGGCAT ATACACTTACTTCATGGCCTACA

>dre-miR-455-5p grp5404-536

TATGTGCCCTTGGACTACATc

>dre-miR-455-3p grp5898-433

GCAGTCCATGGGCATATACACc

>dre-miR-126a-pre 8:12065910-12066049- GCCATTTTAACTGCTTCACAGTCCATTATTACTTTTGGTACGCGCTAGGCCAGACTCAAACTCGTACCGTG AGTAATAATGCACTGTGGCAGTGGGT

>dre-miR-126a-5p grp456-11673

CATTATTACTTTTGGTACGCG

>dre-miR-126a-3p grp6909-3281

TCGTACCGTGAGTAATAATGCA

>dre-miR-10d-pre 6:10777937-10778076+

TTTGTTCCGTCGTCTATATATACCCTGTAGAACCGAATGTGTGTTTACACAGCAAATTCACAGATTCGGTT TTAGGGGAGTATATGGACGATGCAAA

>dre-miR-10d-5p grp1989-365

TACCCTGTAGAACCGAATGTGT

>dre-miR-212-pre 15:25232205-25232344+ GAAAAGTCAGTGCATCAATACCTTGGCTCTAGACTGCTTACTGCTAAATGTGTCTGAAAGTACAGTAACAG TCTACAGTCATGGCTACTGACGTC

>dre-miR-212-5p grp2549-357

ACCTTGGCTCTAGACTGCTTACT

>dre-miR-212-3p grp3061-25

TAACAGTCTACAGTCATGGCT

>dre-miR-212-2-pre 10:36666160-36666299+ (NOVEL) CAGAACTTCATGACCTTGGCTCTAGACTGCTTACTGCTTATGGAGTCCGACAGTACAGTAACAGTCTACAG TCATGGCTACTGAAGTCTG

>dre-miR-212-2-5p grp2549-357

ACCTTGGCTCTAGACTGCTTACT

>dre-miR-212-2-3p grp3061-25

TAACAGTCTACAGTCATGGCT

>dre-miR-130a-pre 10:34405257-34405396- TGTCTGTCCAGTGCCCCTTTTATATTGTACTACTGATAACCCAGTTATTAAAGCAGTGCAATGTTAAAAGG GCATTGGCCAGGGA

>dre-miR-130a-3p grp7910-362

CAGTGCAATGTTAAAAGGGCc

>dre-miR-210-pre 25:26371909-26372048- GTGATTTCTGAAAGCAGGTAAGCCACTGACTAACGCACATTGCGCCTATTCTCCACTCCACTGTGCGTGTG ACAGCGGCTAACCAGCTTTTGGATCTGC

>dre-miR-210-5p grp5058-356

AGCCACTGACTAACGCACATTG

>dre-miR-210-3p grp1437-192

CTGTGCGTGTGACAGCGGCT

>dre-miR-15a-1-pre 1:47219720-47219859- GCGGGGCCTGTCGGTACTGTAGCAGCACAGAATGGTTTGTGAGTTATAACGGGGGTGCAGGCCGTACTGTG CTGCGGCAACAACGACAGGACAGACGC

>dre-miR-15a-1-5p grp1829-337

TAGCAGCACAGAATGGTTTGTG

>dre-miR-19a-2-pre 9:55420576-55420715- (NOVEL) GCAGTTCTCTGCTAGTTTTGCATAGTTGCACTACAAGAAATATTGAGTTGTGCAAATCTATGCAAAACTGA TGGTGGCCTGC

>dre-miR-19a-2-3p grp9320-333

TGTGCAAATCTATGCAAAACTG

>dre-miR-19a-pre 1:2806375-2806514+ GGTGCAGTTCTCTGCTAGTTTTGCATAGTTGCACTACAAGAAAACGGGAGTTGTGCAAATCTATGCAAAAC TGATGGTGGCCTGCTGCT

>dre-miR-19a-3p grp9320-333

TGTGCAAATCTATGCAAAACTG

>dre-miR-96-pre 4:14140068-14140207+ TCTTCTTTGCCTGTTTTGGCACTAGCACATTTTTGCTTTTTTATATATACCTTGAGCAATTATGTGTAGTG CCAATATGGGACAAGACAGA

>dre-miR-96-5p grp2708-317

TTTGGCACTAGCACATTTTTGCT

>dre-miR-7132-pre Zv9_scaffold3487:171731-171870+ (NOVEL) GTTTGGGTCCCTTGACTTGGTCTAAGCTCCTCAGTGTGATGATTAAACCTGAGGAGTTTAGAGCAAGTAAA GGGTCTTCAAC

>dre-miR-7132-5p grp10506-281

GACTTGGTCTAAGCTCCTCAGT

>dre-miR-7132-3p grp6864-284

TGAGGAGTTTAGAGCAAGTAAA

>dre-miR-107b-pre 17:23402945-23403084+ TCTGGCCACTCTGGGCTTCTCTACAGTGTTGCCTTGTAGCCTGGTGATCAAGCAGCATTGTACAGGGCTTT CAGCGTGTACAGA

>dre-miR-107b-3p grp4751-276

AGCAGCATTGTACAGGGCTTTCt

>dre-miR-737-pre 18:44379244-44379383+ CCACAGCTGCTGTGCTGTTGTTTTTTTAGGTTTTGATTTTTGTGAAATGTCGATGAGAAAATCAAAACCTA AAGAAAATACTGCGCAGATAGATGG

>dre-miR-737-5p grp3945-264

GTTTTTTTAGGTTTTGATTTT

>dre-miR-735-pre 24:39680962-39681101- AGAGATTTTGTGCTCTTGTCACGACTAGCCATGTGCGGGTGTAAGATTCTAACGGTACGGCTGGTCCGAAG GCGGTGGGTTAGTCTTCTCT

>dre-miR-735-3p grp2469-243

GGCTGGTCCGAAGGCGGTGGGTTAG

>dre-miR-457b-pre 14:32391760-32391899- CTGAAGGGAATGTACTAAAGCAGCACATAAATACTGGAGGTGATTGTGGTGTTATCCAGTATTGCTGTTCT GCTGTAGTAAGACCTGTCAG

>dre-miR-457b-5p grp11385-80

AAGCAGCACATAAATACTGGAGt

>dre-miR-457b-3p grp1767-212

TCCAGTATTGCTGTTCTGCTGT

>dre-miR-7b-pre 5:56985003-56985142- GCTGGCTTGCTTCTGTGTGGAAGACTTGTGATTTTGTTGTTGTTAGTTAGATGAAGTGACAACAAATCACG GTCTGCCCTACAGCACAGGCCCAGC

>dre-miR-7b-5p grp8085-202

TGGAAGACTTGTGATTTTGTTGTT

>dre-miR-7b-3p grp13439-14

CAACAAATCACGGTCTGCCCT

>dre-miR-34a-pre 23:22885157-22885296+ GTGAGTGGTTCTCTGGCAGTGTCTTAGCTGGTTGTTGTGTGGAGTGAGAACGAAGCAATCAGCAAGTATAC TGCCGCAGAAACTCGTCAC

>dre-miR-34a-5p grp10238-195

TGGCAGTGTCTTAGCTGGTTGT

>dre-miR-223-pre 5:24017684-24017823- CTCTTCTCTTAGAGTATTTGACAGACTGTGGTTGACACTCGATCTAAAGGGGTGTCAGTTTGTCAAATACC CCAAGAGAGGGG

>dre-miR-223-5p grp11766-54

AGAGTATTTGACAGACTGTGGTT

>dre-miR-223-3p grp2755-195

TGTCAGTTTGTCAAATACCCCA

>dre-miR-489-pre 19:41741757-41741896-

TGAGTGGTGGCCTGGTGGTCGTATGTATGACGTCATTTACTTCAAAGTTTGGAGTGACATCATATGTACGG CTGCTAAACTGCTACATGGCTCA

>dre-miR-489-3p grp1733-191

TGACATCATATGTACGGCTGCT

>dre-miR-9-5-pre 5:49952881-49953020- GAATTGGAAGCGAGTTGTTATCTTTGGTTATCTAGCTGTATGAGTATTTTGCACTTCATAAAGCTAGATAA CCGAAAGTAAAAACTGCCTCCTGGCTC

>dre-miR-9-5-5p grp3562-188

TCTTTGGTTATCTAGCTGTATGA

>dre-miR-9-1-pre 16:31444414-31444553+ GGGGTTGGCTGTTATCTTTGGTTATCTAGCTGTATGAGTGTTATTCATTCTTCATAAAGCTAGATAACCGA AAGTAACAAGAATCCC

>dre-miR-9-1-5p grp3562-188

TCTTTGGTTATCTAGCTGTATGA

>dre-miR-9-6-pre 7:15256907-15257046- AGGAGGTAGTTGCTATCTTTGGTTATCTAGCTGTATGAGTGTTTATCTGCCTTCATAAAGCTAGATAACCG AAAGTAGAAATGTCCTCCT

>dre-miR-9-6-5p grp3562-188

TCTTTGGTTATCTAGCTGTATGA

>dre-miR-9-2-pre 10:45056147-45056286- TGGAGGCGTGTTGTTATCTTTGGTTATCTAGCTGTATGAGTGTGCTGGCCGTCATAAAGCTAGATAACCGA AAGTAAGAGCCGCTTCTA

>dre-miR-9-2-5p grp3562-188

TCTTTGGTTATCTAGCTGTATGA

>dre-miR-9-7-pre 2:59942568-59942707- GGGTTAGTTTTTCTCTTTGGTTATCTAGCTGTATGAGTTATGAAATATCATAAAGCTAGAGAACCGAAAGT AGAAACTATACCT

>dre-miR-9-7-5p grp3562-188

TCTTTGGTTATCTAGCTGTATGA

>dre-miR-9-3-pre 25:8759270-8759409- GGGGGTTGGTTTCTCTCTTTGGTTATCTAGCTGTATGAGTTATAACACTGTCATAAAGCTAGATAACCGAA AGTAGAAATAATTCCC

>dre-miR-9-3-5p grp3562-188

TCTTTGGTTATCTAGCTGTATGA

>dre-miR-9-4-pre 22:18478808-18478947+ TGGGTTAGTTTTTCTCTTTGGTTATCTAGCTGTATGAGTTTATGTGATATCATAAAGCTAGAGAACCGAAT GTATAAACTAATTCCA

>dre-miR-9-4-5p grp3562-188

TCTTTGGTTATCTAGCTGTATGA

>dre-miR-132-1-pre 15:25232514-25232653+ CTGTCTCCATGGCGACCGTGGCATTAGATTGTTACTGTAGGAACAGAATTTTTGGTAACAGTCTACAGCCA TGGTCGCTAGTGGGCAG

>dre-miR-132-1-3p grp1607-159

TAACAGTCTACAGCCATGGTCG

>dre-miR-101b-pre 5:1946482-1946621

GTCATGAATTGTCCATTTTCAGTTATCATGGTACCGGTGCTGTGTGCCTGTCAAGTACAGTACTATGATAA

CTGAAGATTGACGGTGCCAAAC

>dre-miR-101b-5p grp3746-152

CAGTTATCATGGTACCGGTGCTGT

>dre-miR-101b-3p grp10354-169

TACAGTACTATGATAACTGAAt

>dre-miR-29a-2-pre 23:20637130-20637269- (NOVEL) GCTTTTACCTTCTGTAGCGTGACTGATTTCCTCTGGTGTTCAGAGTGGACTGGGTTTTTCTAGCACCATTT GAAATCGGTCGTGCTGAAAGGTATGGC

>dre-miR-29a-2-3p grp13456-395

TAGCACCATTTGAAATCGGTt

>dre-miR-429b-pre 6:40583957-40584096- TAAAATCCTCTGATTTGTGTCTTACCAGGCAAGTTTAGATGTTGATACTGTGTCTAATACTGCCTGGTAAT GCCATCAATCACTGGGAATTTG

>dre-miR-429b-3p grp10951-134

TAATACTGCCTGGTAATGCCA

>dre-miR-738-pre 4:49788894-49789033- GGGGTGGCGGTGACTGTGGACGCACGCAGGGCCGTTCTCGTGGATCCCTCTGGCTACGGCCCGCGTCGGGA CCTCTCGTCTAGCGACTTC

>dre-miR-738-3p grp7355-1819 aGCTACGGCCCGCGTCGGGACCT

>dre-miR-29b-1-pre 6:47408906-47409045+ CAATGCTGCTCCTGGAAGCTGAATTCAGATGGTGCCATAGAGTATTTTATGGCATCTAGCACCATTTGAAA TCAGTGTTCCTGGGCCGGTCCTG

>dre-miR-29b-1-5p grp4842-63

GCTGAATTCAGATGGTGCCATAGA

>dre-miR-29b-1-3p grp11990-118

TAGCACCATTTGAAATCAGTGT

>dre-miR-29b-2-pre 4:10672674-10672813- GGACTCTTCCTCCAGATGCTGGTTTCACATGGTGGTTTAGATGTGTTCTACCAAAGTCTAGCACCATTTGA AATCAGTGTTCTTGGGGAGGGATTT

>dre-miR-29b-2-5p grp9067-56

CTGGTTTCACATGGTGGTTTAGA

>dre-miR-29b-2-3p grp11990-118

TAGCACCATTTGAAATCAGTGT

>dre-miR-551-pre 15:34583520-34583659+ (NOVEL) CTGTGTTGCCTTAGAAACCAAGCATGGGTGTGGCCTGAATCCTCCAATGGCGACCCATCCTTGGTTTCTGA GGCTGTACAG

>dre-miR-551-3p grp3070-117

GCGACCCATCCTTGGTTTCTG

>dre-miR-725-pre 22:23643651-23643790+ CTGCAGTGCACATTGCTAGGAATGGTGGCTGAGATGAAGAGCAGGATTTCAGTCATTGTTTCTAGTAGTGC GCGCTGCAG

>dre-miR-725-5p grp10869-105

TGCTAGGAATGGTGGCTGAGAT

>dre-miR-725-3p grp12894-55478

TTCAGTCATTGTTTCTAGTAGT

>dre-miR-133a-1-pre Zv9_scaffold3540:268598-268737+ CATCAAACCACAATGCTTTGCTAAAGCTGGTAAAATGGAACCAAATCACCTCTTCAATGGATTTGGTCCCC TTCAACCAGCTGTAGCTATGCTTTGATG

>dre-miR-133a-1-3p grp7726-94

TTTGGTCCCCTTCAACCAGCTGT

>dre-miR-133a-2-pre 23:7175202-7175341+

AATGCTTTGCTAAAGCTGGTAAAATGGAACCAAATCAACTGTTTTATGGATTTGGTCCCCTTCAACCAGCT GTAGCTGTGCATT

>dre-miR-133a-2-3p grp7726-94

TTTGGTCCCCTTCAACCAGCTGT

>dre-miR-7146-2-pre 13:53977113-53977252+ (NOVEL) TTGAAGAATGCTGTAAACCTGTCACCATTGACTTCTGTAGTACGTTTTTCCTGCTGTGGAAGTCGATGGTT ACCGGTTTCCAACAAAATATCTTCAG

>dre-miR-7146-2-5p grp864-88

TCACCATTGACTTCTGTAGTAa

>dre-miR-1788-pre 9:11827799-11827938- TGTCTTGTATCCGAGGCTTGTTTTAAGTTGCCTGCGATCTCTTAATGACTCAGGCAGCTAAAGCAAGTCTG GGAGGCCAGAGACA

>dre-miR-1788-3p grp2622-81

CAGGCAGCTAAAGCAAGTCTGt

>dre-miR-7133-pre 8:10565084-10565223+ (NOVEL) GATATCAGTGGAGACCTGCTCTGGTGTTGTGTGTTAAACTGTATTCCTAAACAACATCTATAGTTTGATTC ACAGCACAAGAGTTTGCTCTCTGTGTT

>dre-miR-7133-3p grp3660-72

TAGTTTGATTCACAGCACAAG

>dre-miR-730-pre 7:24529205-24529344+ GGGTTCTCCGGTCTCCTCATTGTGCATGCTGTGTGTCTTCAGTCTGGTCCTCACAGCGCCTGCAATGTGGA GGCTAGGGGACTC

>dre-miR-730-5p grp1686-68

TCCTCATTGTGCATGCTGTGTG

>dre-miR-194a-pre 10:27698620-27698759+ AGACTTGGTGCTGACTGCTTGTAACAGCAACTCCATGTGGAAGGTTTGTGTCTTCCAGTGGAGCTGCTGTT GCGTGCAGATAGTCACCATTATCT

>dre-miR-194a-5p grp10468-55

TGTAACAGCAACTCCATGTGGA

>dre-miR-722-1-pre 1:56910609-56910748- GGAACGGAGTGGAATTTGAAACGTTTTGGCCAAAAATGTAGCCATGGCAAAGGGGTTTTTTGCAGAAACGT TTCAGATTTCGCTCCGTTCT

>dre-miR-722-2-3p grp4640-50

TTTTGCAGAAACGTTTCAGATT

>dre-miR-722-2-pre 3:51094766-51094905- (NOVEL) GGAACAGAATGGAATTTGAAACGTTTTAGCCAAAAATGTTTCCATGGTAAAGGTGTTTTTTGCAGAAACGT TTCAGATTTCGTTCTGTTCT

>dre-miR-722-2-3p grp4640-50

TTTTGCAGAAACGTTTCAGATT

>dre-miR-7148-pre 2:56905279-56905418- GCTAATACTAGTAATGGAAATACTCGCTGATACTGCAAGAATTGTGGCATCGGTATAAGCCAGTATTTCCG ATACTAGTATTGGT

>dre-miR-7148-5p grp6573-4

AATGGAAATACTCGCTGATACT

>dre-miR-193a-1-pre 3:44210534-44210673+ ATGTGTTAGAGGTTGGGTCTTTGCGGGCAAGGTGAGTAGTTAAATTTACTCTCAACTGGCCTACAAAGTCC CAGTTTCTGGCTCAT

>dre-miR-193a-1-5p grp491-38

TGGGTCTTTGCGGGCAAGGTGA

>dre-miR-193a-1-3p grp10825-32

AACTGGCCTACAAAGTCCCAGT

>dre-miR-193a-2-pre 6:19392281-19392420+ GTGTGTCAGAGGCTGGGTCTTTGCGGGCAAGGTGAGTTTTCCTTTCATTCAACTGGCCTACAAAGTCCCAG TTTTCGGCCCAT

>dre-miR-193a-2-5p grp491-38

TGGGTCTTTGCGGGCAAGGTGA

>dre-miR-193a-2-3p grp10825-32

AACTGGCCTACAAAGTCCCAGT

>dre-miR-153b-pre 6:13891565-13891704+ CACAGCTGTCTGTGTCATTTTTGTGGTTTGCAGCTAGTAGTCTGGTTCCAGTTGCATAGTCACAAAAATGA GCACAGACAGATGTG

>dre-miR-153b-3p grp12055-33

TTGCATAGTCACAAAAATGAGC

>dre-miR-187-1-pre 19:35551006-35551145+ GACCTGTGGCTGGGCCAGGGGCTGCAACACAGGACATGGGAGCTGTCTCTCACTCCCGCTCGTGTCTTGTG TTGCAGCCAGTGGAACGGCTACACTGTC

>dre-miR-187-1-3p grp8484-203

TCGTGTCTTGTGTTGCAGCCAGT

>dre-miR-363-pre 14:32336520-32336659- CTTGCTGTTTTCGGGTGGATGACTCTGCAATTTTATTAGTGATGGAAAAACTTCAATAAAAATTGCACGGT ATCCATCTGTAATCCGCTGG

>dre-miR-363-3p grp6841-26

AATTGCACGGTATCCATCTGTAt

>dre-miR-218b-pre 10:17725436-17725575+ TGCCCAGGACACCATTGTGCTTGATCTAACCATGCAGTTCCCTTTCTGTCCATGGTTGTGCCAAGCACTTT GGAGACTTGTGTGCA

>dre-miR-218b-5p grp2229-58

TTGTGCTTGATCTAACCATGt

>dre-miR-740-2-pre 22:33895270-33895409+ (NOVEL) CCAAAAAAAGTGGTATGGTACGGTTCGGTACGTCTTTTGACAGTGGAAACGGCCATTAAAGCGTACCAAAC TGAACCGTATCGTACCACGCAGTGG

>dre-miR-740-2-3p grp1790-132

AAAGCGTACCAAACTGAACCG

>dre-miR-20b-pre 14:32337041-32337180- GAGTTTGTCCTGGCAGTTCCAAAGTGCTCACAGTGCAGGTAGTGCCAGTGGATCTACTGCAATGTCTGCAC TTCAAGTATTGCCGGACGCCTTC

>dre-miR-20b-5p grp139-8

CAAAGTGCTCACAGTGCAGGTAG

B) Sequences of most highly expressed mature miRNAs during zebrafish caudal fin regeneration used for differential expression analysis.

>dre-miR-21-1-5p grp3295-4000898

TAGCTTATCAGACTGGTGTTGGC

>dre-miR-462-5p grp6105-1106777

TAACGGAACCCATAATGCAGCTG

>dre-miR-181a-1-5p grp9677-889187

AACATTCAACGCTGTCGGTGAG

>dre-miR-10b-1-5p grp6899-373724

TACCCTGTAGAACCGAATTTGT

>dre-miR-205-5p grp13814-339871

TCCTTCATTCCACCGGAGTCTG

>dre-miR-184-1-3p grp12307-328479

TGGACGGAGAACTGATAAGGGC

>dre-miR-146a-5p grp8319-288109

TGAGAACTGAATTCCATAGATGG

>dre-let-7a-1-5p grp6832-257492

TGAGGTAGTAGGTTGTATAGTT

>dre-let-7d-1-5p grp8315-5107

TGAGGTAGTTGGTTGTATGGTT

>dre-miR-26a-1-5p grp5057-221336

TTCAAGTAATCCAGGATAGGCT

>dre-miR-22a-3p grp5792-151660

AAGCTGCCAGCTGAAGAACTGT

>dre-miR-204-2-5p grp6554-150213

TTCCCTTTGTCATCCTATGCCT

>dre-miR-92a-1-3p grp2213-133685

TATTGCACTTGTCCCGGCCTGT

>dre-miR-27b-3p grp237-94815

TTCACAGTGGCTAAGTTCTGC

>dre-miR-26b-5p grp422-71912

TTCAAGTAATCCAGGATAGGTT

>dre-miR-100-1-5p grp5254-66492

AACCCGTAGATCCGAACTTGTG

>dre-miR-222a-3p grp5652-59743

AGCTACATCTGGCTACTGGGTCTC

>dre-miR-181b-1-5p grp11650-57168

AACATTCATTGCTGTCGGTGGGT

>dre-miR-203b-3p grp5500-43712

TGAAATGTTCAGGACCACTTGA

>dre-let-7e-1-5p grp6695-48480

TGAGGTAGTAGATTGAATAGTT

>dre-miR-143-1-3p grp11379-30699

TGAGATGAAGCACTGTAGCTC

>dre-miR-30d-5p grp2092-43821

TGTAAACATCCCCGACTGGAAGCT

>dre-miR-2184-1-5p grp10995-43606

AACAGTAAGAGTTTATGTGCTG

>dre-miR-27c-1-3p grp2038-40890

TTCACAGTGGTTAAGTTCTG

>dre-miR-199-1-3p grp11262-38570

ACAGTAGTCTGCACATTGGTT

>dre-miR-10c-5p grp497-30749

TACCCTGTAGATCCGGATTTGT

>dre-let-7f-5p grp409-28668

TGAGGTAGTAGATTGTATAGTT

>dre-miR-30e-2-5p grp13468-24136

TGTAAACATCCTTGACTGGAAGCT

>dre-miR-25-3p grp2813-23111

CATTGCACTTGTCTCGGTCTGA

>dre-miR-146b-5p grp2365-23045

TGAGAACTGAATTCCAAGGGTGT

>dre-miR-141-3p grp7153-22896

TAACACTGTCTGGTAACGATG

>dre-miR-182-5p grp6070-20665

TTTGGCAATGGTAGAACTCACACT

>dre-miR-30b-5p grp8125-4284

TGTAAACATCCTACACTCAGCT

>dre-miR-30c-5p grp4783-17761

TGTAAACATCCTACACTCTCAGCT

>dre-let-7b-1-5p grp1603-13607

TGAGGTAGTAGGTTGTGTGGTT

>dre-miR-125a-2-5p grp26-12535

TCCCTGAGACCCTTAACCTGTG

>dre-miR-429a-3p grp5596-12213

TAATACTGTCTGGTAATGCCG

>dre-miR-125b-1-5p grp10313-12150

TCCCTGAGACCCTAACTTGTGA

>dre-miR-125c-5p grp7517-4196

TCCCTGAGACCCTAACTCGTGA

>dre-let-7g-1-5p grp5039-11780

TGAGGTAGTAGTTTGTATAGTT

>dre-miR-375-1-3p grp1291-11742

TTTGTTCGTTCGGCTCGCGTTA

>dre-miR-126a-5p grp456-11673

CATTATTACTTTTGGTACGCG

>dre-miR-203a-3p grp9139-10633

GTGAAATGTTTAGGACCACTTG

>dre-miR-27a-5p grp4524-10144

AGGACTTAGCTCACTCTGTGAACA

>dre-miR-15b-5p grp67-9972

TAGCAGCACATCATGGTTTGTA

>dre-let-7c-1-5p grp5778-9899

TGAGGTAGTAGGTTGTATGGTT

>dre-miR-101b-3p grp12015-9175

TACAGTACTGTGATAACTGAAG

>dre-let-7h-5p grp2268-8111

TGAGGTAGTAAGTTGTGTTGTT

>dre-miR-214-3p grp129-7915

ACAGCAGGCACAGACAGGCAGt

>dre-miR-1388-5p grp2681-7795

AGGACTGTCCAACCTGAGAATG

>dre-miR-221-1-3p grp4811-7549

AGCTACATTGTCTGCTGGGTTT

>dre-miR-16c-3p grp1479-7419

TCCAATATTGCTCGTGCTGCTGt

>dre-miR-23a-1-3p grp12800-7080

ATCACATTGCCAGGGATTTCC

>dre-miR-338-1-3p grp8564-6698

TCCAGCATCAGTGATTTTGTT

>dre-miR-142a-5p grp669-6556

CATAAAGTAGAAAGCACTACT

>dre-miR-92b-3p grp878-6416

TATTGCACTCGTCCCGGCCTCC

>dre-miR-183-5p grp10435-6387

TATGGCACTGGTAGAATTCACT

>dre-miR-200a-3p grp7153-22896

TAACACTGTCTGGTAACGATG

>dre-miR-200b-3p grp4559-6313

TAATACTGCCTGGTAATGATGc

>dre-miR-200c-3p grp4559-6313

TAATACTGCCTGGTAATGATGC

>dre-miR-99-1-5p grp13110-6097

AACCCGTAGATCCGATCTTGTG

>dre-miR-152-3p grp12231-5676

TCAGTGCATGACAGAACTTTGt

>dre-miR-16b-5p grp4945-5356

TAGCAGCACGTAAATATTGGAGT

>dre-miR-148-3p grp6766-5131

TCAGTGCATTACAGAACTTTGT

>dre-miR-454b-3p grp11024-4503

TAGTGCAATATTGCTTATAGGGTC

>dre-miR-196a-1-5p grp7025-3693

TAGGTAGTTTCATGTTGTTGGG

>dre-miR-128-1-3p grp2325-3564

TCACAGTGAACCGGTCTCTTT

>dre-miR-31-5p grp10983-3372

TGGCAAGATGTTGGCATAGCTG

>dre-miR-27e-3p grp3960-3368

TTCACAGTGGCTAAGTTCAGT

>dre-miR-140-3p grp9767-3235

ACCACAGGGTAGAACCACGGACt

>dre-miR-181c-5p grp2236-3214

CACATTCATTGCTGTCGGTGGGTT

>dre-miR-15c-5p grp4853-302

AAGCAGCGCGTCATGGTTTTCA

>dre-miR-19b-3p grp9643-2971

TGTGCAAATCCATGCAAAACTG

>dre-miR-19c-3p grp3312-18

TGTGCAAATCCATGCAAAACTCG

>dre-miR-27d-3p grp3639-67077

TTCACAGTGGCTAAGTTCTg

>dre-miR-460-3p grp4879-2744

CACAGCGCATACAATGTGGATG

>dre-miR-23b-3p grp5274-2648

ATCACATTGCCAGGGATTACCACt

>dre-miR-458-3p grp6805-2455

ATAGCTCTTTGAATGGTACTGC

>dre-miR-22b-3p grp1011-2442

AAGCTGCCAGTTGAAGAGCTGT

>dre-miR-139-5p grp12872-2363

TCTACAGTGCATGTGTCTCCAGT

>dre-miR-142b-5p grp10620-2309

CATAAAGTAGACAGCACTACT

>dre-miR-193b-3p grp12804-2174

AACTGGCCCGCAAAGTCCCGCT

>dre-miR-455-1-5p grp567-2171

TATGTGCCCTTGGACTACATCG

>dre-miR-107a-3p grp11902-2033

AGCAGCATTGTACAGGGCTATC

>dre-miR-130b-5p grp13715-1912

ACTCTTTCCCTGTTGCACTACT

>dre-miR-10a-5p grp1421-1753

TACCCTGTAGATCCGAATTTGT

>dre-miR-301a-3p grp9137-1746

CAGTGCAATAGTATTGTCAAAGC

>dre-miR-130c-1-3p grp2237-1702

CAGTGCAATATTAAAAGGGCA

>dre-miR-222b-3p grp3723-1575

AGCTACATCTGAATACTGGGTC

>dre-miR-192-5p grp4190-1480

ATGACCTATGAATTGACAGCCA

>dre-miR-456-3p grp9652-134

CAGGCTGGTTAGATGGTTGTCt

>dre-miR-29a-3p grp9484-1303

TAGCACCATTTGAAATCGGTTA

>dre-miR-24-1-3p grp12832-2176

TGGCTCAGTTCAGCAGGAAC

>dre-miR-365-1-3p grp4946-1276

TAATGCCCCTAAAAATCCTTAT

>dre-miR-196b-5p grp12773-1252

TAGGTAGTTTCAAGTTGTTGGGC

>dre-miR-155-5p grp11819-1231

TTAATGCTAATCGTGATAGGGGT

>dre-miR-457a-5p grp9332-1143

AAGCAGCACATCAATATTGGCA

>dre-miR-724-5p grp4562-1087

TTAAAGGGAATTTGCGACTGTT

>dre-miR-196d-5p grp11987-964

TAGGTAGTTTTATGTTGTTGGGt

>dre-miR-196c-3p grp2688-950

CGACAACAAGAAACTGCCTTGA

>dre-miR-16a-5p grp6244-922

TAGCAGCACGTAAATATTGGTG

>dre-miR-20a-2-5p grp8336-909

TAAAGTGCTTATAGTGCAGGTAG

>dre-miR-132-1-3p grp6716-858

ACCGTGGCTTTAGATTGTTACT

>dre-miR-17a-1-5p grp1621-842

CAAAGTGCTTACAGTGCAGGTAG

>dre-miR-145-5p grp7945-803

GTCCAGTTTTCCCAGGAATCCCT

>dre-miR-731-5p grp9254-109911

AATGACACGTTTTCTCCCGGATCG

>dre-miR-454a-3p grp1916-709

TAGTGCAATATTGCTAATAGGGT

>dre-miR-218a-1-5p grp9287-619

TTGTGCTTGATCTAACCATGTG

>dre-miR-93-5p grp394-595

AAAAGTGCTGTTTGTGCAGGTAG

>dre-miR-150-5p grp12186-532

TCTCCCAATCCTTGTACCAGTG

>dre-let-7j-5p grp12629-8982

TGAGGTAGTTGTTTGTACAGTT

>dre-miR-19d-3p grp493-492

TGTGCAAACCCATGCAAAACTG

>dre-miR-10d-5p grp1989-365

TACCCTGTAGAACCGAATGTGT

>dre-miR-212-5p grp2549-357

ACCTTGGCTCTAGACTGCTTACT

>dre-miR-130a-3p grp7910-362

CAGTGCAATGTTAAAAGGGCc

>dre-miR-210-5p grp5058-356

AGCCACTGACTAACGCACATTG

>dre-miR-15a-1-5p grp1829-337

TAGCAGCACAGAATGGTTTGTG

>dre-miR-19a-2-3p grp9320-333

TGTGCAAATCTATGCAAAACTG

>dre-miR-96-5p grp2708-317

TTTGGCACTAGCACATTTTTGCT

>dre-miR-7132-3p grp6864-284

TGAGGAGTTTAGAGCAAGTAAA

>dre-miR-737-5p grp3945-264

GTTTTTTTAGGTTTTGATTTT

>dre-miR-735-3p grp2469-243

GGCTGGTCCGAAGGCGGTGGGTTAG

>dre-miR-457b-3p grp1767-212

TCCAGTATTGCTGTTCTGCTGT

>dre-miR-7b-5p grp8085-202

TGGAAGACTTGTGATTTTGTTGTT

>dre-miR-34a-5p grp10238-195

TGGCAGTGTCTTAGCTGGTTGT

>dre-miR-223-3p grp2755-195

TGTCAGTTTGTCAAATACCCCA

>dre-miR-489-3p grp1733-191

TGACATCATATGTACGGCTGCT

>dre-miR-9-1-5p grp3562-188

TCTTTGGTTATCTAGCTGTATGA

>dre-miR-429b-3p grp10951-134

TAATACTGCCTGGTAATGCCA

>dre-miR-738-3p grp7355-1819

aGCTACGGCCCGCGTCGGGACCT

>dre-miR-29b-1-3p grp11990-118

TAGCACCATTTGAAATCAGTGT

>dre-miR-551-3p grp3070-117

GCGACCCATCCTTGGTTTCTG

>dre-miR-725-3p grp12894-55478

TTCAGTCATTGTTTCTAGTAGT

>dre-miR-133a-1-3p grp7726-94

TTTGGTCCCCTTCAACCAGCTGT

>dre-miR-7146-2-5p grp864-88

TCACCATTGACTTCTGTAGTAa

>dre-miR-1788-3p grp2622-81

CAGGCAGCTAAAGCAAGTCTGt

>dre-miR-7133-3p grp3660-72

TAGTTTGATTCACAGCACAAG

>dre-miR-730-5p grp1686-68

TCCTCATTGTGCATGCTGTGTG

>dre-miR-194a-5p grp10468-55

TGTAACAGCAACTCCATGTGGA

>dre-miR-722-2-3p grp4640-50

TTTTGCAGAAACGTTTCAGATT

>dre-miR-7148-5p grp6573-4

AATGGAAATACTCGCTGATACT

>dre-miR-193a-1-5p grp491-38

TGGGTCTTTGCGGGCAAGGTGA

>dre-miR-153b-3p grp12055-33

TTGCATAGTCACAAAAATGAGC

>dre-miR-187-1-3p grp8484-203

TCGTGTCTTGTGTTGCAGCCAGT

>dre-miR-363-3p grp6841-26

AATTGCACGGTATCCATCTGTAt

>dre-miR-218b-5p grp2229-58

TTGTGCTTGATCTAACCATGt

>dre-miR-740-2-3p grp1790-132

AAAGCGTACCAAACTGAACCG

>dre-miR-20b-5p grp139-8

CAAAGTGCTCACAGTGCAGGTAG

C) Mature miRNAs expressed in bichir pectoral fin regeneration.

>pse-miR-21-1-5p grp27078-848326

TAGCTTATCAGACTGGTGTTGGC

>pse-miR-184-1-3p grp3176-94574

TGGACGGAGAACTGATAAGGGT

>pse-miR-30e-2-5p grp20855-43838

TGTAAACATCCTCGACTGGAAGC

>pse-miR-146a-5p grp344-42179

TGAGAACTGTTTTCCATAGATGG

>pse-miR-205-5p grp13674-38161

TCCTTCATTCCACCGGAGTCTG

>pse-miR-203a-3p grp2362-37792

GTGAAATGTTTAGGACCACTTG

>pse-miR-101b-3p grp21471-33136

TACAGTACTGTGATAACTGAAG

>pse-miR-148-3p grp8048-26408

TCAGTGCATTACAGAACTTTGT

>pse-miR-143-1-3p grp27582-26017

TGAGATGAAGCACTGTAGCT

>pse-miR-725-3p grp5673-25584

TTCAGTCATTGTTTCTGGTAGA

>pse-let-7e-1-5p grp7205-23916

TGAGGTAGTAGATTGAATAGTT

>pse-miR-30d-5p grp26228-23659

TGTAAACATCCCCGACTGGAAGC

>pse-let-7a-1-5p grp4680-19987

TGAGGTAGTAGGTTGTATAGTT

>pse-miR-140-3p grp22770-19325

TACCACAGGGTAGAACCACGGA

>pse-miR-10b-1-5p grp6353-18277

TACCCTGTAGAACCGAATTTGT

>pse-let-7f-5p grp16378-16145

TGAGGTAGTAGATTGTATAGTT

>pse-miR-200a-3p grp3463-15996

TAACACTGTCTGGTAACGATGTT

>pse-miR-181a-1-5p grp20393-14701

AACATTCAACGCTGTCGGTGAGT

>pse-let-7d-1-5p grp3225-14473

TGAGGTAGTTGGTTGTATAGTT

>pse-miR-26b-5p grp8342-12887

TTCAAGTAATCCAGGATAGG

>pse-miR-27d-3p grp20129-11199

TTCACAGTGGCTAAGTTCCGC

>pse-miR-200c-3p grp25772-10503

TAATACTGCCTGGTAATGATGA

>pse-miR-142a-5p grp22529-10406 (miR-142-3p) TGTAGTGTTTCCTACTTTATGG

>pse-miR-130c-1-3p grp11589-10261

CAGTGCAATAATGAAAGGGCGT

>pse-miR-25-3p grp10433-10224

CATTGCACTTGTCTCGGTCTGA

>pse-miR-26a-1-5p grp7099-10020

TTCAAGTAATCCAGGATAGGCT

>pse-miR-16b-5p grp2614-9162

AAGCAGCACGTATATACTGGAG

>pse-miR-375-1-3p grp3522-9012

TTTGTTCGTTCGGCTCGCGTTA

>pse-miR-24-1-3p grp9625-8875

TGGCTCAGTTCAGCAGGAACAGT

>pse-miR-30c-5p grp7080-8850

TGTAAACATCCTACACTCTCAGCT

>pse-miR-462-5p grp2170-7730

TAACGGAACCCATAATGCAGCTG

>pse-miR-133a-1-3p grp13373-5941

TTGGTCCCCTTCAACCAGCTGT

>pse-miR-107a-3p grp1655-5893

AGCAGCATTGTACAGGGCTATGA

>pse-let-7h-5p grp13469-5610

TGAGGTAGTAAGTTGTATTGTT

>pse-miR-23a-1-3p grp22181-5549

ATCACATTGCCAGGGATTTCCA

>pse-miR-92a-1-3p grp26140-5488

TATTGCACTTGTCCCGGCCTGT

>pse-miR-199-1-3p grp26073-5046 (miR-199c-5p)

CCCAGTGTTCAGACTACCTGTTC

>pse-miR-182-5p grp19569-4660

TTTGGCAATGGTAGAACTCACA

>pse-miR-150-5p grp5958-4277

TCTCCCAACCCTTGTACCAGTGT

>pse-miR-126a-5p grp25475-3954 (miR-126-3p)

TCGTACCGTGAGTAATAATGC

>pse-miR-30b-5p grp8127-3656

TGTAAACATCCTACACTCAGCT

>pse-miR-429a-3p grp17976-3504

TAATACTGTCTGGTAATGCCGT

>pse-miR-125b-1-5p grp19587-3183

TCCCTGAGACCCTAACTTGTGA

>pse-miR-93-5p grp4029-3140

CAAAGTGCTGTTTGTGCAGGTAG

>pse-miR-223-3p grp21765-3006

TGTCAGTTTGTCAAATACCCCA

>pse-miR-19d-3p grp14355-2983

TGTGCAAACCCATGCAAAACTGA

>pse-miR-20a-2-5p grp22125-2885

TAAAGTGCTTATAGTGCAGGTAG

>pse-miR-181c-5p grp21835-2551

AACATTCATTGCTGTCGGTGGGTT

>pse-miR-19b-3p grp18406-2335

TGTGCAAATCCATGCAAAACTGA

>pse-miR-1388-5p grp24270-2326

AGGACTGTCTAACCTGAGAATG

>pse-miR-365-1-3p grp2938-2093

TAATGCCCCTAAAAATCCTTAT

>pse-miR-183-5p grp8286-1997

TATGGCACTGGTAGAATTCACT

>pse-let-7j-5p grp22396-1958

TGAGGTAGTTGTTTGTACAGTT

>pse-miR-2184-1-5p grp15092-1913

AACAGTAAGAGTTTATGTGTTG

>pse-let-7c-1-5p grp20203-1908

TGAGGTAGTAGGTTGTATGGTT

>pse-miR-17a-1-5p grp26100-1854

CAAAGTGCTTACAGTGCAGGTAG

>pse-miR-130b-5p grp21997-1838

ACTCTTTCACTGTTGCGCTACT

>pse-miR-457a-5p grp7609-1755

TAGCAGCACTTCAATATTGGGA

>pse-miR-22b-3p grp9521-1716

AAGCTGCCAGTTGAAGAACTGT

>pse-miR-181b-1-5p grp27615-1644

AACATTCATTGCTGTCGGTGGGT

>pse-miR-27a-5p grp20573-1574

AGGGCTTAGTTCACTGGTGAACA

>pse-miR-100-1-5p grp3653-1562

AACCCGTAGATCCGAACTTGT

>pse-miR-193b-3p grp10188-1549

AACTGGCCCGCAAAGTCCCGCT

>pse-miR-145-5p grp8748-1484

GTCCAGTTTTCCCAGGAATCCC

>pse-miR-222a-3p grp5018-1420

AGCTACATCTGGCTACTGGGTCT

>pse-let-7g-1-5p grp4238-1293

TGAGGTAGTAGTTTGTGCTGTT

>pse-miR-15b-5p grp13766-1236

CAGCAGCACATCATGATTTGGA

>pse-miR-363-3p grp961-1232

AATTGCACGGTATCCATCTGTA

>pse-miR-221-1-3p grp22058-1603 (miR-221-5p)

ACCTGGCATACAATGTAGATTTCT

>pse-miR-125a-2-5p grp17602-1204

TCCCTGAGACCCTTAACCTGTGA

>pse-let-7b-1-5p grp7732-1185

TGAGGTAGTAGGTTGTGTGGTT

>pse-miR-99-1-5p grp18571-1063

CACCCGTAGATCCGATCTTGTG

>pse-miR-20b-5p grp3256-1062

AAAAGTGCTCACAGTGCAGGTAG

>pse-miR-27b-3p grp25131-1021

TTCACAGTGGCTAAGTTCTGC

>pse-miR-152-3p grp8967-848

TCAGTGCATGACAGAACTTTTG

>pse-miR-29a-3p grp9970-832

TAGCACCATTTGAAATCGGTTA

>pse-miR-31-5p grp24882-954 (miR-31-3p)

TGCTATGCCTACATACTGCCATC

>pse-miR-29b-1-3p grp22787-767

TAGCACCATTTGAAATCAGTTA

>pse-miR-737-5p grp8766-674

GTTTTTTTAGGTTTTGATTTT

>pse-miR-192-5p grp4728-653

ATGACCTATGAATTGACAGCC

>pse-miR-10a-5p grp25211-595

TACCCTGTAGATCCGAATTTGT

>pse-miR-19a-2-3p grp24868-591

TGTGCAAATCTATGCAAAACTGA

>pse-miR-196a-1-5p grp6357-591

TAGGTAGTTTCATGTTGTTGGG

>pse-miR-16a-5p grp18477-583

TAGCAGCACGTAAATATTGGTG

>pse-miR-203b-3p grp17819-582 (miR-203b-5p)

AGTGGTTCTAGACAGTTCAACA

>pse-miR-34a-5p grp27520-557

TGGCAGTGTCTTAGCTGGTTGT

>pse-miR-204-2-5p grp1308-555

TTCCCTTTGTCATCCTATGCCT

>pse-miR-7b-5p grp15169-548

TGGAAGACTAGTGATTTTGTTGTT

>pse-miR-92b-3p grp6841-515

TATTGCACTCGTCCCGGCCTCC

>pse-miR-128-1-3p grp7780-471

TCACAGTGAACCGGTCTCTTT

>pse-miR-458-3p grp24666-454

ATAGCTCTTTGAATGGTACTGC

>pse-miR-455-1-5p grp18997-448

TATGTGCCCTTGGACTACATCG

>pse-miR-139-5p grp5061-413

TCTACAGTGCATGTGTCTCCAGT

>pse-miR-731-5p grp2188-394

AATGACACGATTTCTCCCGGTTT

>pse-miR-193a-1-5p grp23633-391

TGGGTCTTTGCGGGCGAGGCGA

>pse-miR-338-1-3p grp24338-321

TCCAGCATCAGTGATTTTGTTG

>pse-miR-19c-3p grp11525-309

TGTGCAAATCCATGCAAAACTCA

>pse-miR-210-5p grp9704-323 (miR-210-3p)

CTGTGCGTGTGACAGCGGCTAA

>pse-miR-222b-3p grp3964-1311 (miR-222b-5p)

TGCTCAGTAGTCAGTGTAGATT

>pse-miR-301a-3p grp12360-282 (miR-301a-5p)

TCTGACAATGTTGCACTACT

>pse-miR-130a-3p grp26928-262

CAGTGCAATAATGAAAGGGCGTATC

>pse-miR-155-5p grp8545-243

TTAATGCTAATCGTGATAGGGGTT

>pse-miR-454b-3p grp18556-238

TAGTGCAATATTGCTTATAGGGT

>pse-miR-96-5p grp25285-212

TTTGGCACTAGCACATTTTTGCT

>pse-miR-15a-1-5p grp8327-196

TAGCAGCACATAATGGTTTGTG

>pse-miR-9-1-5p grp23598-152

TCTTTGGTTATCTAGCTGTATGA

>pse-miR-214-3p grp3460-137

ACAGCAGGCACAGACAGGCAGT

>pse-miR-456-3p grp12674-129

CAGGCTGGTTAGATGGTTGTCC

>pse-miR-27e-3p grp25010-107

TTCACAGTGGCTAAGTTCCGCAT

>pse-miR-460-3p grp15158-101 (miR-460a-5p) CCTGCATTGTACACACTGTGTG

>pse-miR-730-5p grp9848-91

TCCTCATTGTACATGCTGTGTG

>pse-miR-218a-1-5p grp27463-80

TTGTGCTTGATCTAACCATGTG

>pse-miR-551-3p grp318-73

GCGACCCATTCTTGGTTTCAGT

>pse-miR-454a-3p grp8589-44

TAGTGCAATATTGCTTATAGG

>pse-miR-218b-5p grp11221-35

TTGTGCTTGATCTAACCATGT

>pse-miR-722-2-3p ggrp3111-24

TTTTTTGCAGAAACGTTTCAGA

>pse-miR-7133-3p grp20630-22

TAGTTTGATTTACAGCACGAGA

>pse-miR-1788-3p grp18242-18

CAGGCAGCTAAAGCAAGTCTGT

>pse-miR-187-1-3p grp22136-16

TCGTGTCTTGTGTTGCAGCCAGT

>pse-miR-153b-3p grp23764-8

TTGCATAGTCACAAAAGTGATC

>pse-miR-16c-3p grp16126-5

CCCAATATTACTTGTGCTGCTTCA

>pse-miR-1b grp13671-85835

TGGAATGTAAAGAAGTATGTCT

>pse-miR-206 grp23914-31839

TGGAATGTAAGGAAGTGTGTGG

>pse-miR-30a-3p grp16359-12781

CTTTCAGTCGGATGTTTGCAGC

>pse-miR-451-5p grp10286-2597

AAACCGTTACCATTACTGAG

>pse-miR-425-3p grp308-1475

CATCGGGACTGTCGTGTCAACC

>pse-miR-22a-3p grp8479-1470 (miR-22a-5p)

AGTTCTTCACTGGCAAGCTTTA

>pse-miR-144-5p grp9598-1248

GGATATCATCATATACTGTAAG

>pse-miR-499-5p grp26182-870

TTAAGACTTGCAGTGATGTTT

>pse-miR-34c-5p grp14668-621

AGGCAGTGTAGTTAGCTGATTGC

>pse-miR-2188-5p grp6703-385

GAGGTCCGGCCTCACGTGTCCT

>pse-miR-208-3p grp21990-302

ATAAGACGAGCAAAAAGTGTGT

>pse-miR-200b grp11423-267

TAATACTGCCTGGTAATGATGAATC

>pse-miR-24b-5p grp4515-232

TGCCTACTGAGCTGATAACAGT

>pse-miR-135a-5p grp11377-226

TATGGCTTTTTATTCCTATCTGA

>pse-miR-18a-5p grp23360-193

TAAGGTGCATCTAGTGCAGATAG

>pse-miR-217-5p grp7768-144

TACTGCATCAGGAACTGATTGGA

>pse-miR-33a-5p grp20086-77

GTGCATTGTGGTTGCATTGC

>pse-miR-106a-5p grp14535-61

TAAAGTGCTTACAGTGCAGG

>pse-miR-132-1-3p grp21544-58

ACCATGGCTGTAGACTGTTACC

>pse-miR-190a-5p grp5936-48

TGATATGTTTGATATATTAGG

>pse-miR-1329-5p grp21922-43

TACAGTGATCAGGTTACGATGG

>pse-miR-138-5p grp20044-35

AGCTGGTGTTGTGAATCAGGC

>pse-miR-736-3p grp27690-34

ATAAGACGAACAAAAAGTTTGT

>pse-miR-147-5p grp14335-26

ACGGAATCATTTCTGCACAAACTC

>pse-miR-430c-3p grp1935-20

TAAGTGCTTGTTTGTTGGGTCTT

>pse-miR-727-3p grp22177-17

GTTGAGGCGAGCTGAAGACT

>pse-miR-875-5p grp16205-13

TATACCTCAGTCTTTTCAGGTGT

>pse-miR-137 grp23373-13

TTATTGCTTAAGAATACGCGTAG

>pse-miR-211 grp12180-11

TTCCCTTTGTCTTCCTATGCCTATC

>pse-miR-302 grp16049-11

TAAGTGCTTTTCTGTTTGAGCGA

>pse-miR-2187-5p grp25653-10

TTTAATTAGTATAGCCTGTATT

>pse-miR-315-5p grp11876-9

TTTTGATTGTTGCTCAGAGAGTC

>pse-miR-1306-3p grp21459-7

TGGACGTTGGCTCCGGTGGTGA

D) Mature miRNAs expressed in axolotl forelimb regeneration.

>ame-miR-21-1-5p grp6404-240235

TAGCTTATCAGACTGATGTTGAC

>ame-miR-140-3p grp17696-99696

TACCACAGGGTAGAACCACGGA

>ame-miR-148-3p grp4510-81014

TCAGTGCACTACAGAACTTTGT

>ame-miR-203a-3p grp777-43618

GTGAAATGTTTAGGACCACTTG

>ame-miR-30e-2-5p grp17057-31035

TGTAAACATCCTCGACTGGAAGC

>ame-miR-93-5p grp17375-25639

CAAAGTGCTGTTCGTGCAGGTAG

>ame-miR-143-1-3p grp9720-23867

TGAGATGAAGCACTGTAGCT

>ame-miR-130c-1-3p grp13825-20962

CAGTGCAATAATGAAAGGGCGT

>ame-let-7a-1-5p grp1657-20140

TGAGGTAGTAGGTTGTATAGTT

>ame-miR-27a-5p grp5402-20061

AGGACTTAGCTCACTTTGTGAAC

>ame-miR-10b-1-5p grp12022-14931

TACCCTGTAGAACCGAATTTGT

>ame-miR-30d-5p grp9231-13953

TGTAAACATCCCCGACTGGAAGC

>ame-miR-101b-3p grp17258-13736

TACAGTACTGTGATAACTGAAG

>ame-miR-181a-1-5p grp16897-13051

AACATTCAACGCTGTCGGTGAGT

>ame-let-7f-5p grp5773-13017

TGAGGTAGTAGATTGTATAGTT

>ame-miR-25-3p grp13394-11043

CATTGCACTTGTCTCGGTCTGA

>ame-miR-133a-1-3p grp14422-10074

TTGGTCCCCTTCAACCAGCTGT

>ame-miR-200c-3p grp18729-9245

TAATACTGCCTGGTAATGATGA

>ame-miR-27b-3p grp18472-5738

TTCACAGTGGCTAAGTTCTGC

>ame-miR-375-1-3p grp1233-5329

TTTGTTCGTTCGGCTCGCGTTA

>ame-miR-182-5p grp16618-4656

TTTGGCAATGGTAGAACTCACA

>ame-miR-107a-3p grp553-4454

AGCAGCATTGTACAGGGCTATGA

>ame-miR-30c-5p grp12251-4292

TGTAAACATCCTACACTCTCAGCT

>ame-miR-26a-1-5p grp2533-4227

TTCAAGTAATCCAGGATAGGCT

>ame-miR-205-5p grp14528-4191

TCCTTCATTCCACCGGAGTCTG

>ame-miR-200a-3p grp18634-4138

TAACACTGTCTGGTAACGATGT

>ame-let-7g-1-5p grp8901-4008

TGAGGTAGTAGTTTGTGTAGTT

>ame-miR-184-1-3p grp10884-3853

TGGACGGAGAACTGATAAGGGT

>ame-miR-100-1-5p grp14569-3833

AACCCGTAGATCCGAACTTGC

>ame-miR-146a-5p grp4399-3532

TGAGAACTGAATTCCATGGACT

>ame-miR-183-5p grp2958-3508

TATGGCACTGGTAGAATTCACT

>ame-miR-26b-5p grp12709-3424

TTCAAGTAATCCAGGATAGG

>ame-miR-92a-1-3p grp9200-3199

TATTGCACTTGTCCCGGCCTGT

>ame-miR-725-3p grp5841-3183

TTCAGTCATTTTTTCTGGTGGA

>ame-miR-199-1-3p grp9181-3985 (miR-199c-5p)

CCCAGTGTTCAGACTACCTGTTC

>ame-let-7j-5p grp11539-3078

TGAGGTAGTAGTTTGTACAGTT

>ame-miR-24-1-3p grp12482-2925

TGGCTCAGTTCAGCAGGAACAG

>ame-miR-20b-5p grp13948-2754

CAAAGTGCTCATAGTGCAGGTAG

>ame-miR-27d-3p grp18885-2610

TTCACAGTGGCTAAGTTCTG

>ame-miR-23a-1-3p grp17506-2494

ATCACATTGCCAGGGATTTCCA

>ame-miR-125b-1-5p grp6927-2393

TCCCTGAGACCCTAACTTGTGA

>ame-miR-429a-3p grp6347-2372

TAATACTGTCTGGTAATGCCGT

>ame-miR-19d-3p grp14759-2261

TGTGCAAACCCATGCAAAACTGA

>ame-miR-196d-5p grp2524-2206

TAGGTAGTTTTATGTTGTTGGG

>ame-miR-16b-5p grp5361-2149

TAGCAGCACGTAAATATTGGAG

>ame-let-7c-1-5p grp7133-2029

TGAGGTAGTAGGTTGTATGGTT

>ame-miR-10a-5p grp8878-1955

TACCCTGTAGATCCGAATTTGT

>ame-miR-126a-5p grp796-1944 (miR-126)

TCGTACCGTGAGTAATAATGCG

>ame-let-7b-1-5p grp4071-1541

TGAGGTAGTAGGTTGTGTGG

>ame-miR-193b-3p grp13318-1485

AACTGGCCCGCAAAGTCCCGCT

>ame-miR-19b-3p grp16196-1451

TGTGCAAATCCATGCAAAACTGA

>ame-let-7e-1-5p grp16464-1406

TGAGGTAGTGGATTGAATAGTT

>ame-miR-181b-1-5p grp19391-1327

AACATTCATTGCTGTCGGTGG

>ame-miR-30b-5p grp2896-1081

TGTAAACATCCTACACTCAGCT

>ame-miR-20a-2-5p grp16246-996

TAAAGTGCTTATAGTGCATGTAG

>ame-miR-17a-1-5p grp18845-993

CAAAGTGCTTACAGTGCAGGTAG

>ame-miR-223-3p grp17369-892

TGTCAGTTTGTCAAATACCCCA

>ame-miR-301a-3p grp14667-879

CAGTGCAATGATATTGTCAAAGC

>ame-miR-142a-5p grp7959-852 (miR-142-3p)

TGTAGTGTTTCCTACTTTATGG

>ame-miR-365-1-3p grp10807-811

TAATGCCCCTAAAAATCCTTAT

>ame-miR-221-1-3p grp14032-694

AGCTACATTGTCTGCTGGGTTT

>ame-miR-181c-5p grp7734-669

AACATTCATTGCTGTCGGTGGGTT

>ame-miR-16a-5p grp6515-651

TAGCAGCACGTAAATATTGGTG

>ame-miR-363-3p grp305-500

AATTGCACGGTATCCATCTGTA

>ame-miR-15b-5p grp1818-467

TAGCAGCACATCATGGTTTGC

>ame-miR-130a-3p grp2631-453

CAGTGCAATGTTAAAAGGGCAT

>ame-miR-22b-3p grp3381-418

AAGCTGCCAGTTGAAGAACTGT

>ame-miR-155-5p grp658-267

TTAATGCTAATCGTGATAGG

>ame-miR-455-1-5p grp6703-303

TATGTGCCCTTGGACTACATCG

>ame-miR-214-3p grp11876-298

TACAGCAGGCACAGACAGGCAG

>ame-miR-96-5p grp14710-291

TTTGGCACTAGCACATTTTTGC

>ame-miR-125a-2-5p grp15904-287

TCCCTGAGACCCTTAACCTGTGA

>ame-miR-130b-5p grp12400-286

ACTCTTTCTCTGTTGCACTACT

>ame-miR-204-2-5p grp434-265

TTCCCTTTGTCATCCTATGCCT

>ame-miR-99-1-5p grp6341-243

AACCCGTAGATCCGATCTTGT

>ame-miR-1388-5p grp2398-235

AGGACTGTTTGACCTGAGAATGG

>ame-miR-145-5p grp15956-235

GTCCAGTTTTCCCAGGAATCCCT

>ame-miR-34a-5p grp19350-222

TGGCAGTGTCTTAGCTGGTTGT

>ame-miR-454b-3p grp6540-221

TAGTGCAATATTGCTTATAGGGT

>ame-miR-192-5p grp13121-211

ATGACCTATGAATTGACAGC

>ame-miR-15a-1-5p grp3753-193

TAGCAGCACGTAATGGTTTGTG

>ame-miR-460-3p grp5343-190 (miR-460a-5p)

CCTGCATTGTACACACTGTGTG

>ame-miR-29a-3p grp13241-184

TAGCACCATTTGAAATCGGTTA

>ame-miR-454a-3p grp12779-184

TAGTGCAATATTGCTTATAGG

>ame-miR-152-3p grp10051-165

TCAGTGCATGACAGAACTTTGT

>ame-miR-222a-3p grp11555-157

AGCTACATCTGGCTACTGGGTCT

>ame-miR-139-5p grp1800-153

TCTACAGTGCATGTGTCTCCAGT

>ame-miR-456-3p grp10900-138

CAGGCTGGTTAGATGGTTGTCT

>ame-miR-19a-2-3p grp18387-127

TGTGCAAATCTATGCAAAACTGA

>ame-miR-196a-1-5p grp6570-126

TAGGTAGTTTTATGTTGTTG

>ame-miR-7b-5p grp5349-125

TGGAAGACTAGTGATTTTGTTGTT

>ame-miR-128-1-3p grp2775-124

TCACAGTGAACCGGTCTCTTT

>ame-miR-218a-1-5p grp19329-102

TTGTGCTTGATCTAACCATGTG

>ame-miR-31-5p grp5637-99

AGGCAAGATGTTGGCATAGCTGA

>ame-miR-9-1-5p grp5344-96

TCTTTGGTTATCTAGCTGTATG

>ame-miR-222b-3p grp4362-96

AGCTACATCTGGCTACTGGG

>ame-miR-218b-5p grp3990-88

TTGTGCTTGATCTAACCATGT

>ame-miR-338-1-3p grp9403-72

TCCAGCATCAGTGATTTTGTTGA

>ame-miR-203b-3p grp17169-55

GAAATGTTTAGGACCACTTG

>ame-miR-150-5p grp16910-41

TCTCCCAACCCTTGTACCAGTG

>ame-miR-19c-3p grp4569-40

TGTGCAAATCCATGCAAAACT

>ame-let-7h-5p grp979-30

TGAGGTAGTAGTTTGTGTTGTT

>ame-miR-29b-1-3p grp7079-27

TAGCACCATATGAAATCAGTGT

>ame-miR-132-1-3p grp10119-23

TAACAGTCTACAGCCATGGTCG

>ame-miR-458-3p grp2393-21

ATAGCTCTTTAAATGGTACTGC

>ame-miR-1788-3p grp18126-20

CAGGCAGCTAAAGCAAGTCTG

>ame-miR-2184-1-5p grp1566-17

AACAGTAAGAGTTAATGTGCTG

>ame-miR-146b-5p grp14103-12

TGAGAACTGAATTCCATGGGCTGT

>ame-miR-194a-5p grp16249-10

TGTAACAGCAACTCCATGTGGAC

>ame-miR-206 grp18075-45402

TGGAATGTAAGGAAGTGTGTGG

>ame-miR-1b grp13975-16327

TGGAATGTAAAGAAGTATGTAT

>ame-miR-1a-3p grp18792-9222

TGGAATGTAAAGAAGTATGTAC

>ame-miR-30a-3p grp5766-2431

CTTTCAGTCGGATGTTTGCAGC

>ame-miR-1d-3p grp6097-1227

TGGAATGTTAAGAAGTATGTAT

>ame-miR-191-5p grp3176-530

CAACGGAATCCCAAAAGCAGCTG

>ame-miR-499-5p grp4921-398

TTAAGACTTGCAGTGATGTTTA

>ame-miR-210-5p grp13159-206 (miR-210-3p)

CTGTGCGTGTGACAGCGGCTAA

>ame-miR-200b grp14567-137

CATCTTACTGGGCAGCATTGG

>ame-miR-138-5p grp16784-107

AGCTGGTGTTGTGAATCAGGC

>ame-miR-2970-5p grp13071-104

GACAGTCAGTAGTTGGTCTGG

>ame-miR-144-5p grp172-80

GGATATCATCATATACTGTAAGT

>ame-miR-1329-5p grp7763-78

TACAGTGATCAGGTTACGATGG

>ame-miR-18b-5p grp12601-74

TAAGGTGCATCTAGTGCAGTTAG

>ame-miR-29d-5p grp3238-71

TCTGGTTTCACGTGGTGGCTTAGA

>ame-miR-425-5p grp15710-55

AATGACACGATCACTCCCGTTGAGC

>ame-miR-451-5p grp13870-50

TAACCGTTACCATTACTGAG

>ame-miR-34c-5p grp2498-40

AGGCAGTGTAGTTAGCTGATTG

>ame-miR-217-5p grp2770-27

TACTGCATCAGGAACTGATTGGA

>ame-miR-205a-3p grp10586-26

ATTCCCAGTGGGATGAAGCTC

>ame-miR-106b-5p grp6832-25

AAAAGTGCTTACAGTGCAGG

>ame-miR-106a-5p grp15250-20

TAAAGTGCTTACAGTGCAAG

>ame-miR-1662 grp1803-19

TTGACATCATCATACTTGGGAT

>ame-miR-2188-5p grp4545-17

AAGGTCCAACCTCATATATCCT

>ame-miR-208b-3p grp5032-17

ATAAGACGAACAAAAGGTTTGT

>ame-miR-328-5p grp5989-16

GGGGGGCAGGTGGCTCTCCG

>ame-miR-137 grp17887-15

TTATTGCTTAAGAATACGCGTAG

>ame-miR-281 grp6426-13

TGTCATGGAGTTGCTCTCGTGA

>ame-let-7i-3p grp12406-13

CTGCGCAAGCTACTGCCTTG

>ame-miR-33a-5p grp12834-12

GTGCATTGTAGTTGCATTGC

>ame-miR-135-5p grp13181-10

TATGGCTTTTTATTCCTATGTGA

>ame-miR-203c grp671-10

GTGAACTGTTTAGGACCACTTG

>ame-miR-2954-3p grp659-8

CATCCCCATTCCACTCCTAGCAG

>ame-miR-124-4-3p grp15960-7

TAAGGCACGCGGTGAATGCTAA
