# Supplementary material for: A Conserved MicroRNA Regulatory Circuit Is Differentially Controlled during Limb/Appendage Regeneration
Source: PLoS One. 2016 Jun 29;11(6):e0157106. doi: 10.1371/journal.pone.0157106 (PMC4927183; doi:10.1371/journal.pone.0157106)
Supplement: S2 File — Predicted miR-21 binding sites predicted by miRanda [75] within 3’-UTRs for zebrafish, bichir and axolotl target genes. The 3’-UTRs were taken from Ensembl (version 76) for zebrafish while the 3’-UTRs for bichir and axolotl were derived from open reading frame predictions from annotated de novo transcriptome assemblies. (DOC) [file pone.0157106.s003.doc]

S2 File: Predicted  miR-21 Binding Sites for Genes in Figure 6A.

pdcd4b (Zebrafish)

  
Read Sequence:ENSDARG00000041022_ENSDART00000009223_pdcd4b_programmed cell death 4b [Source:ZFIN;Acc:ZDB-GENE-030131-9847](624 nt)
=-=-=-=-=-=-=-=-=-=-=-=-=-=-=-=-=-=-=-=-=-=-=-=-=-=-=-=-=-=-=-= Performing Scan: dre-miR-21-1-5p vs ENSDARG00000041022_ENSDART00000009223_pdcd4b_programmed
=-=-=-=-=-=-=-=-=-=-=-=-=-=-=-=-=-=-=-=-=-=-=-=-=-=-=-=-=-=-=-=

Forward: 	Score: 146.000000 	Q:2 to 15 	R:190 to 212 Align Len
(13) (76.92%) (76.92%)

Query: 	3' cgguuguggUCAGACUAUUCGAu 5'
| || 	||||||| Ref:	5' ctccttctgACTCGTATAAGCTa 3'

Energy: 	-13.390000 kCal/Mol

pdcd4 (Bichir)

  
Read Sequence:lcl|comp44967_c0_seq3:1496-2131 comp44967_c0_seq3 len=2131 path=[52782472:0-1294 52800961:1295-1675 52840714:1676-1688
52806021:1689-2130](636 nt)
=-=-=-=-=-=-=-=-=-=-=-=-=-=-=-=-=-=-=-=-=-=-=-=-=-=-=-=-=-=-=-= Performing Scan: pse-miR-21-1-5p vs lcl|comp44967_c0_seq3:1496-2131
=-=-=-=-=-=-=-=-=-=-=-=-=-=-=-=-=-=-=-=-=-=-=-=-=-=-=-=-=-=-=-=

Forward: 	Score: 157.000000 	Q:2 to 20 	R:404 to 428 Align Len
(20) (75.00%) (80.00%)

Query: 	3' cgguUGU-GGUCA-GACUAUUCGAu 5'
|:| ||| | || |||||||
Ref: 		5' gttcATATCCAATGCTTATAAGCTa 3' Energy: 	-16.930000 kCal/Mol
pdcd4 (Axolotl)

  
Read Sequence:lcl|comp24370_c0_seq1:1566-2176 comp24370_c0_seq1 len=2176 path=[31274315:0-2175](611 nt)
=-=-=-=-=-=-=-=-=-=-=-=-=-=-=-=-=-=-=-=-=-=-=-=-=-=-=-=-=-=-=-= Performing Scan: ame-miR-21 vs lcl|comp24370_c0_seq1:1566-2176
=-=-=-=-=-=-=-=-=-=-=-=-=-=-=-=-=-=-=-=-=-=-=-=-=-=-=-=-=-=-=-=

Forward: 	Score: 143.000000 	Q:2 to 16 	R:194 to 216 Align Len
(14) (64.29%) (78.57%)

Query: 	3' caguuguaGUCAGACUAUUCGAu 5'
:| |: 	||||||| Ref:	5' cagtggtgTATTTAAATAAGCTa 3'

Energy: 	-10.780000 kCal/Mol

tgfbr2 (Zebrafish)

Read Sequence:ENSDARG00000038097_ENSDART00000055539_pigq_phosphatidylinosito l glycan, class Q [Source:ZFIN;Acc:ZDB-GENE-030131-9793](782 nt)
=-=-=-=-=-=-=-=-=-=-=-=-=-=-=-=-=-=-=-=-=-=-=-=-=-=-=-=-=-=-=-=
Performing Scan: dre-miR-21-1-5p vs
ENSDARG00000038097_ENSDART00000055539_pigq_phosphatidylinositol
=-=-=-=-=-=-=-=-=-=-=-=-=-=-=-=-=-=-=-=-=-=-=-=-=-=-=-=-=-=-=-=

Forward: 	Score: 140.000000 	Q:2 to 18 	R:450 to 473 Align Len
(17) (70.59%) (82.35%)

Query: 	3' cgguugUGGUCA-GACUAUUCGAu 5'
:|| 	| ||||||:||| Ref:	5' tttttaGCCTCTCCTGATAGGCTa 3'

Energy: 	-20.690001 kCal/Mol

tgfbr2 (Bichir)

Read Sequence:lcl|comp59856_c0_seq1:315-2441 comp59856_c0_seq1 len=2441 path=[133368766:0-2440](2127 nt)
=-=-=-=-=-=-=-=-=-=-=-=-=-=-=-=-=-=-=-=-=-=-=-=-=-=-=-=-=-=-=-= Performing Scan: pse-miR-21-1-5p vs lcl|comp59856_c0_seq1:315-2441
=-=-=-=-=-=-=-=-=-=-=-=-=-=-=-=-=-=-=-=-=-=-=-=-=-=-=-=-=-=-=-=

Forward: 	Score: 140.000000 	Q:2 to 22 	R:86 to 110 Align Len
(22) (54.55%) (81.82%)

Query: 	3' cgGUUGUGGUC--AGACUAUUCGAu 5'
:||::|::| 	| |||:|||| Ref:	5' ttTAATGCTGGAAGCAGATGAGCTc 3'

Energy: 	-15.670000 kCal/Mol

tgfbr2 (Axolotl)

Read Sequence:lcl|comp40567_c0_seq1:2376-5397 comp40567_c0_seq1 len=5397 path=[105615921:0-639 105625612:640-1056 105789335:1057-1069
105633029:1070-1489 105640017:1490-3608 105673391:3609-3615
105673486:3616-5060 105696286:5061-5063 105809337:5064-5396](3022 nt)
=-=-=-=-=-=-=-=-=-=-=-=-=-=-=-=-=-=-=-=-=-=-=-=-=-=-=-=-=-=-=-= Performing Scan: ame-miR-21 vs lcl|comp40567_c0_seq1:2376-5397
=-=-=-=-=-=-=-=-=-=-=-=-=-=-=-=-=-=-=-=-=-=-=-=-=-=-=-=-=-=-=-=

Forward: 	Score: 158.000000 	Q:2 to 19 	R:1687 to 1709 Align Len
(17) (64.71%) (88.24%)

Query: 	3' caguuGUAGUCAGACUAUUCGAu 5'
::|:: || |||||||| Ref:	5' ttttcTGTTGTTCGGATAAGCTa 3'

Energy: 	-13.780000 kCal/Mol

blc2l13 (Zebrafish)


Read Sequence:ENSDARG00000062370_ENSDART00000090156_bcl2l13_BCL2-like
13 (apoptosis facilitator) [Source:ZFIN;Acc:ZDB-GENE-050419-215](1106 nt)
=-=-=-=-=-=-=-=-=-=-=-=-=-=-=-=-=-=-=-=-=-=-=-=-=-=-=-=-=-=-=-= Performing Scan: dre-miR-21-1-5p vs ENSDARG00000062370_ENSDART00000090156_bcl2l13_BCL2-like
=-=-=-=-=-=-=-=-=-=-=-=-=-=-=-=-=-=-=-=-=-=-=-=-=-=-=-=-=-=-=-=

Forward: 	Score: 147.000000 	Q:2 to 12 	R:835 to 857 Align Len
(10) (90.00%) (90.00%)

Query: 	3' cgguuguggucaGACUAUUCGAu 5'
|| ||||||| Ref:	5' catttgtaaaaaCTAATAAGCTa 3'

Energy: 	-11.020000 kCal/Mol

bcl2l13 (Bichir) T-bulge

Read Sequence:lcl|comp43682_c0_seq1:1110-3464 comp43682_c0_seq1 len=3464 path=[49858089:0-195 49860574:196-1827 49882800:1828-
3463](2355 nt)
=-=-=-=-=-=-=-=-=-=-=-=-=-=-=-=-=-=-=-=-=-=-=-=-=-=-=-=-=-=-=-= Performing Scan: pse-miR-21-1-5p vs lcl|comp43682_c0_seq1:1110-3464
=-=-=-=-=-=-=-=-=-=-=-=-=-=-=-=-=-=-=-=-=-=-=-=-=-=-=-=-=-=-=-=

Forward: 	Score: 165.000000 	Q:2 to 20 	R:1150 to 1172 Align Len
(18) (77.78%) (88.89%)

Query: 	3' cgguuGUGGUCAGACUAUUUCGAu 5'
:|||||| | :||||||| Ref:	5' tatttTACCAGT-TAGTAAAGCTc 3'

Energy: 	-18.430000 kCal/Mol


bcl2l13 (Axolotl) T-bulge

Read Sequence:lcl|comp34749_c0_seq1:1614-4871 comp34749_c0_seq1 len=4871 path=[63840031:0-4870](3258 nt)
=-=-=-=-=-=-=-=-=-=-=-=-=-=-=-=-=-=-=-=-=-=-=-=-=-=-=-=-=-=-=-= Performing Scan: ame-miR-21 vs lcl|comp34749_c0_seq1:1614-4871
=-=-=-=-=-=-=-=-=-=-=-=-=-=-=-=-=-=-=-=-=-=-=-=-=-=-=-=-=-=-=-=

Forward: 	Score: 140.000000 	Q:2 to 22 	R:242 to 266 Align Len
(21) (66.67%) (71.43%)

Query: 	3' cagUUGUAGUCAGACU-AUUUCGAu 5'
|| | || | 	|| |||||:| Ref:	5' aaaAAGAGCAATGGGATTAAAGTTa 3'

Energy: 	-10.120000 kCal/Mol

rgs5a (Zebrafish) T-bulge


Read Sequence:ENSDARG00000002644_ENSDART00000016586_rgs5a_regulator of
G-protein signaling 5a [Source:ZFIN;Acc:ZDB-GENE-030131-7570](2135 nt)
=-=-=-=-=-=-=-=-=-=-=-=-=-=-=-=-=-=-=-=-=-=-=-=-=-=-=-=-=-=-=-= Performing Scan: dre-miR-21-1-5p vs ENSDARG00000002644_ENSDART00000016586_rgs5a_regulator
=-=-=-=-=-=-=-=-=-=-=-=-=-=-=-=-=-=-=-=-=-=-=-=-=-=-=-=-=-=-=-=

Forward: 	Score: 153.000000 	Q:2 to 23 	R:1302 to 1331 Align Len
(27) (55.56%) (74.07%)

Query: 	3' cgGUUGUGGUCAGACU------AUUUCGAu 5'
:|| :|::||:||| 	||||||| Ref:	5' ttTAAGGCTGGTTTGAGGTCATTAAAGCTa 3'

Energy: 	-21.320000 kCal/Mol

rgs5 (Bichir)

Read Sequence:lcl|comp57032_c0_seq16:c1896-1 comp57032_c0_seq16 len=2515 path=[104957835:0-1009 104850602:1010-1033 104918025:1034-
2514](1896 nt)
=-=-=-=-=-=-=-=-=-=-=-=-=-=-=-=-=-=-=-=-=-=-=-=-=-=-=-=-=-=-=-= Performing Scan: dre-miR-21-1-5p vs lcl|comp57032_c0_seq16:c1896-1
=-=-=-=-=-=-=-=-=-=-=-=-=-=-=-=-=-=-=-=-=-=-=-=-=-=-=-=-=-=-=-=

Forward: 	Score: 144.000000 	Q:2 to 18 	R:1365 to 1388 Align Len
(17) (76.47%) (82.35%)

Query: 	3' cgguugUGGUCA-GACUAUUCGAu 5'
| |||| | |||:|||| Ref:	5' agctgaAACAGTCCAGATGAGCTg 3'

Energy: 	-23.530001 kCal/Mol

rgs5 (Axolotl) T-bulge

Read Sequence:lcl|comp39582_c0_seq1:638-1930 comp39582_c0_seq1 len=1930 path=[95147841:0-676 95268965:677-687 95165720:688-695 95165900:696-839
95169057:840-919 95171188:920-956 95269056:957-1009 95269091:1010-1414
95269226:1415-1421 95185479:1422-1422 95185526:1423-1425 95185587:1426-
1929](1293 nt)
=-=-=-=-=-=-=-=-=-=-=-=-=-=-=-=-=-=-=-=-=-=-=-=-=-=-=-=-=-=-=-= Performing Scan: ame-miR-21 vs lcl|comp39582_c0_seq1:638-1930
=-=-=-=-=-=-=-=-=-=-=-=-=-=-=-=-=-=-=-=-=-=-=-=-=-=-=-=-=-=-=-=

Forward: 	Score: 143.000000 	Q:3 to 22 	R:695 to 717 Align Len
(19) (68.42%) (84.21%)

Query: 	3' cagUUGUAGUCAGACUAUUUCGau 5'
|::|||| |:| 	||||||
Ref: 		5' gaaAGTATCA-TTTTTTAAAGCaa 3' Energy: 	-11.480000 kCal/Mol
chka (Zebrafish)


Read Sequence:ENSDARG00000041078_ENSDART00000060015_chka_choline  kinase alpha [Source:ZFIN;Acc:ZDB-GENE-050208-149](1934 nt)
=-=-=-=-=-=-=-=-=-=-=-=-=-=-=-=-=-=-=-=-=-=-=-=-=-=-=-=-=-=-=-= Performing Scan: dre-miR-21-1-5p vs ENSDARG00000041078_ENSDART00000060015_chka_choline
=-=-=-=-=-=-=-=-=-=-=-=-=-=-=-=-=-=-=-=-=-=-=-=-=-=-=-=-=-=-=-=

Forward: 	Score: 143.000000 	Q:3 to 22 	R:877 to 898 Align Len
(19) (68.42%) (84.21%)

Query: 	3' cgGUUGUGGUCAGACUAUUCGau 5'
:||:|: | || |||||||
Ref: 		5' ttTAATATGAATC-GATAAGCgt 3' Energy: 	-14.260000 kCal/Mol


chka (Bichir)

Read Sequence:lcl|comp50032_c0_seq1:1498-2757 comp50032_c0_seq1 len=2757 path=[67561945:0-582 67572583:583-583 67572596:584-2756](1260 nt)
=-=-=-=-=-=-=-=-=-=-=-=-=-=-=-=-=-=-=-=-=-=-=-=-=-=-=-=-=-=-=-= Performing Scan: dre-miR-21-1-5p vs lcl|comp50032_c0_seq1:1498-2757
=-=-=-=-=-=-=-=-=-=-=-=-=-=-=-=-=-=-=-=-=-=-=-=-=-=-=-=-=-=-=-=

Forward: 	Score: 147.000000 	Q:2 to 18 	R:426 to 447 Align Len
(16) (68.75%) (81.25%)

Query: 	3' cgguugUGGUCAGACUAUUCGAu 5'
::|| | 	||||||||
Ref: 		5' tttatgGTCACT-GGATAAGCTg 3' Energy: 	-14.830000 kCal/Mol


chka (Axolotl) T-bulge

Read Sequence:lcl|comp36746_c0_seq2:6107-8879 comp36746_c0_seq2 len=8879 path=[75098730:0-775 75112222:776-4920 75187741:4921-5191
74762921:5192-5192 74762932:5193-5397 75230530:5398-8177 74815907:8178-
8878](2773 nt)
=-=-=-=-=-=-=-=-=-=-=-=-=-=-=-=-=-=-=-=-=-=-=-=-=-=-=-=-=-=-=-= Performing Scan: ame-miR-21 vs lcl|comp36746_c0_seq2:6107-8879
=-=-=-=-=-=-=-=-=-=-=-=-=-=-=-=-=-=-=-=-=-=-=-=-=-=-=-=-=-=-=-=

Forward: 	Score: 146.000000 	Q:2 to 23 	R:1350 to 1373 Align Len
(21) (66.67%) (71.43%)

Query: 	3' caGUUGUAGUCAGACUAUUUCGAu 5'
||||| | 	| | |||||:|
Ref: 		5' taCAACAACCCACCGTTAAAGTTg 3' Energy: 	-11.890000 kCal/Mol

eif4a1b (Zebrafish) T-bulge

  
Read Sequence:ENSDARG00000003032_ENSDART00000011878_eif4a1b_eukaryotic translation initiation factor 4A, isoform 1B [Source:ZFIN;Acc:ZDB-GENE-
040120-6](987 nt)
=-=-=-=-=-=-=-=-=-=-=-=-=-=-=-=-=-=-=-=-=-=-=-=-=-=-=-=-=-=-=-= Performing Scan: dre-miR-21-1-5p vs ENSDARG00000003032_ENSDART00000011878_eif4a1b_eukaryotic
=-=-=-=-=-=-=-=-=-=-=-=-=-=-=-=-=-=-=-=-=-=-=-=-=-=-=-=-=-=-=-=

Forward: 	Score: 147.000000 	Q:2 to 16 	R:751 to 774 Align Len
(14) (71.43%) (78.57%)

Query: 	3' cgguuguggUCAGACUAUUUCGAu 5'
| |: 	|||||||| Ref:	5' gagggggaaATTTCAATAAAGCTa 3'

Energy: 	-10.590000 kCal/Mol

eif4a1 (Bichir)

Read Sequence:lcl|comp59465_c0_seq1:1241-1829 comp59465_c0_seq1 len=1829 path=[132264148:0-1828](589 nt)
=-=-=-=-=-=-=-=-=-=-=-=-=-=-=-=-=-=-=-=-=-=-=-=-=-=-=-=-=-=-=-=
Performing Scan: dre-miR-21-1-5p vs lcl|comp59465_c0_seq1:1241-1829
=-=-=-=-=-=-=-=-=-=-=-=-=-=-=-=-=-=-=-=-=-=-=-=-=-=-=-=-=-=-=-=

Forward: 	Score: 146.000000 	Q:2 to 19 	R:507 to 529 Align Len
(17) (64.71%) (88.24%)

Query: 	3' cgguuGUGGUCAGACUAUUCGAu 5'
::| || :|||||:||| Ref:	5' actctTGCGAGCTTGATAGGCTg 3'

Energy: 	-19.280001 kCal/Mol


eif4a1 (Axolotl) T-bulge

Read Sequence:lcl|comp15628_c0_seq1:1280-1816 comp15628_c0_seq1 len=1816 path=[18219494:0-1815](537 nt)
=-=-=-=-=-=-=-=-=-=-=-=-=-=-=-=-=-=-=-=-=-=-=-=-=-=-=-=-=-=-=-= Performing Scan: ame-miR-21 vs lcl|comp15628_c0_seq1:1280-1816
=-=-=-=-=-=-=-=-=-=-=-=-=-=-=-=-=-=-=-=-=-=-=-=-=-=-=-=-=-=-=-=

Forward: 	Score: 141.000000 	Q:2 to 22 	R:496 to 518 Align Len
(21) (71.43%) (76.19%)

Query: 	3' cagUUGUAGUCAGAC-UAUUUCGAu 5'
|| || | 	||| ||||||:| Ref:	5' ttgAAGATAA--CTGAATAAAGTTt 3'

Energy: 	-11.010000 kCal/Mol

mapk14b (Zebrafish)


Read Sequence:ENSDARG00000028721_ENSDART00000030921_mapk14b_mitogen- activated protein kinase 14b [Source:ZFIN;Acc:ZDB-GENE-021007-1](2872 nt)
=-=-=-=-=-=-=-=-=-=-=-=-=-=-=-=-=-=-=-=-=-=-=-=-=-=-=-=-=-=-=-= Performing Scan: dre-miR-21-1-5p vs ENSDARG00000028721_ENSDART00000030921_mapk14b_mitogen-activated
=-=-=-=-=-=-=-=-=-=-=-=-=-=-=-=-=-=-=-=-=-=-=-=-=-=-=-=-=-=-=-=

Forward: 	Score: 154.000000 	Q:2 to 21 	R:292 to 318 Align Len
(23) (65.22%) (78.26%)

Query: 	3' cggUUGU-GGUCAGAC---UAUUCGAu 5'
| || |::||:|| 	||||||| Ref:	5' cagATCAGCTGGTTTGTCTATAAGCTg 3'

Energy: 	-19.459999 kCal/Mol


mapk14 (Bichir) T-bulge

Read Sequence:lcl|comp54814_c0_seq2:c1700-1 comp54814_c0_seq2 len=2987 path=[89385695:0-1629 89408210:1630-2019 89471049:2020-2095
89414875:2096-2986](1700 nt)
=-=-=-=-=-=-=-=-=-=-=-=-=-=-=-=-=-=-=-=-=-=-=-=-=-=-=-=-=-=-=-= Performing Scan: dre-miR-21-1-5p vs lcl|comp54814_c0_seq2:c1700-1
=-=-=-=-=-=-=-=-=-=-=-=-=-=-=-=-=-=-=-=-=-=-=-=-=-=-=-=-=-=-=-=

Forward: 	Score: 141.000000 	Q:3 to 23 	R:1367 to 1391 Align Len
(21) (61.90%) (80.95%)

Query: 	3' cgGUUGUGGUC-AGACUAUUUCGau 5'
:||:||: | |: 	|||||||
Ref: 		5' ttTAATACTTGATTCCATAAAGCaa 3' Energy: 	-10.960000 kCal/Mol
mapk14 (Axolotl)

Read Sequence:lcl|comp36834_c0_seq2:c2496-1 comp36834_c0_seq2 len=3730 path=[75287889:0-143 75290385:144-369 75294268:370-2055 75324977:2056-
2463 75332705:2464-2816 75339134:2817-2895 75340519:2896-3729](2496 nt)
=-=-=-=-=-=-=-=-=-=-=-=-=-=-=-=-=-=-=-=-=-=-=-=-=-=-=-=-=-=-=-= Performing Scan: ame-miR-21 vs lcl|comp36834_c0_seq2:c2496-1
=-=-=-=-=-=-=-=-=-=-=-=-=-=-=-=-=-=-=-=-=-=-=-=-=-=-=-=-=-=-=-=

Forward: 	Score: 142.000000 	Q:2 to 21 	R:2051 to 2072 Align Len
(19) (52.63%) (78.95%)

Query: 	3' cagUUGUAGUCAGACUAUUCGAu 5'
|:::|::| 	|||||||
Ref: 		5' aagAGTGTTGG-AACATAAGCTa 3' Energy: 	-11.620000 kCal/Mol
mbpb (Zebrafish) T-bulge


Read Sequence:ENSDARG00000089413_ENSDART00000130081_mbpb_myelin  basic protein b [Source:ZFIN;Acc:ZDB-GENE-030429-21](1390 nt)
=-=-=-=-=-=-=-=-=-=-=-=-=-=-=-=-=-=-=-=-=-=-=-=-=-=-=-=-=-=-=-= Performing Scan: dre-miR-21-1-5p vs ENSDARG00000089413_ENSDART00000130081_mbpb_myelin
=-=-=-=-=-=-=-=-=-=-=-=-=-=-=-=-=-=-=-=-=-=-=-=-=-=-=-=-=-=-=-=

Forward:	Score: 142.000000	Q:2 to 11	R:1011 to 1034 Align Len	
(9) (88.89%)	(88.89%)			

Query: 	3' cgguuguggucagaCUAUUUCGAu 5'
| ||||||| Ref:	5' tatgatggttaaaaGCTAAAGCTg 3'

Energy: 	-10.250000 kCal/Mol


mbp (Bichir) T-bulge

Read Sequence:lcl|comp40143_c0_seq1:c1045-1 comp40143_c0_seq1 len=2239 path=[43248988:0-1207 43267221:1208-1240 43267549:1241-1394
43269785:1395-2238](1045 nt)
=-=-=-=-=-=-=-=-=-=-=-=-=-=-=-=-=-=-=-=-=-=-=-=-=-=-=-=-=-=-=-= Performing Scan: dre-miR-21-1-5p vs lcl|comp40143_c0_seq1:c1045-1
=-=-=-=-=-=-=-=-=-=-=-=-=-=-=-=-=-=-=-=-=-=-=-=-=-=-=-=-=-=-=-=

Forward: 	Score: 146.000000 	Q:2 to 23 	R:972 to 995 Align Len
(21) (57.14%) (66.67%)

Query: 	3' cgGUUGUGGUCAGACUAUUUCGAu 5'
|||:: 	|| 	|||||||
Ref: 		5' gtCAATGAATGTAAATTAAAGCTt 3' Energy: 	-13.400000 kCal/Mol


mbp (Axolotl)

Read Sequence:lcl|comp41181_c0_seq1:c5271-1 comp41181_c0_seq1 len=7124 path=[114863279:0-754 114883031:755-891 115149945:892-1651
115336134:1652-1670 115171154:1671-2200 115185486:2201-2231
115186270:2232-2380 115364361:2381-2381 115332818:2382-2420
115190855:2421-2566 115194499:2567-2646 115196472:2647-2778
115199753:2779-2781 115199975:2782-3104 115208056:3105-3220
115210605:3221-3239 115210981:3240-5287 115266988:5288-5304
115267230:5305-5361 115268939:5362-5362 115268982:5363-5889
115432730:5890-5994 115286167:5995-6001 115286197:6002-6406
115296958:6407-6430 115297574:6431-6442 115297872:6443-6754
115306159:6755-6757 115306186:6758-7034 115312932:7035-7042
115362480:7043-7059 115313648:7060-7123](5271 nt)
=-=-=-=-=-=-=-=-=-=-=-=-=-=-=-=-=-=-=-=-=-=-=-=-=-=-=-=-=-=-=-= Performing Scan: ame-miR-21 vs lcl|comp41181_c0_seq1:c5271-1
=-=-=-=-=-=-=-=-=-=-=-=-=-=-=-=-=-=-=-=-=-=-=-=-=-=-=-=-=-=-=-=

Forward: 	Score: 146.000000 	Q:2 to 21 	R:2276 to 2297 Align Len
(19) (68.42%) (84.21%)

Query: 	3' cagUUGUAGUCAGACUAUUCGAu 5'
||:: |||| 	|||:||||
Ref: 		5' acaAATGACAGT-AGATGAGCTt 3' Energy: 	-17.969999 kCal/Mol


mfn2 (Zebrafish)

Read Sequence:ENSDARG00000079504_ENSDART00000108662_mfn2_mitofusin 2 [Source:ZFIN;Acc:ZDB-GENE-081105-44](3053 nt)
=-=-=-=-=-=-=-=-=-=-=-=-=-=-=-=-=-=-=-=-=-=-=-=-=-=-=-=-=-=-=-= Performing Scan: dre-miR-21-1-5p vs ENSDARG00000079504_ENSDART00000108662_mfn2_mitofusin
=-=-=-=-=-=-=-=-=-=-=-=-=-=-=-=-=-=-=-=-=-=-=-=-=-=-=-=-=-=-=-=

Forward: 	Score: 145.000000 	Q:2 to 20 	R:2788 to 2809 Align Len
(18) (66.67%) (88.89%)

Query: 	3' cgguUGUGGUCAGACUAUUCGAu 5'
|:|:: || ||:|||||| Ref:	5' tggtATATT-GTATGGTAAGCTg 3'

Energy: 	-16.850000 kCal/Mol


mfn2 (Bichir) T-bulge

Read Sequence:lcl|comp27831_c1_seq1:c2473-1 comp27831_c1_seq1 len=4856 path=[29886083:0-3683 29944117:3684-4698 29960123:4699-4855](2473 nt)
=-=-=-=-=-=-=-=-=-=-=-=-=-=-=-=-=-=-=-=-=-=-=-=-=-=-=-=-=-=-=-= Performing Scan: dre-miR-21-1-5p vs lcl|comp27831_c1_seq1:c2473-1
=-=-=-=-=-=-=-=-=-=-=-=-=-=-=-=-=-=-=-=-=-=-=-=-=-=-=-=-=-=-=-=

Forward: 	Score: 149.000000 	Q:2 to 20 	R:961 to 983 Align Len
(18) (72.22%) (72.22%)

Query: 	3' cgguuGUGGUCAGACUAUUUCGAu 5'
||| | | | 	|||||||
Ref: 		5' aacttCACAAAT-TACTAAAGCTc 3' Energy: 	-9.630000 kCal/Mol


mfn2 (Axolotl) T-bulge

Read Sequence:lcl|comp28960_c0_seq1:2569-3983 comp28960_c0_seq1 len=3983 path=[42450272:0-233 42530206:234-340 42455135:341-468
42457607:469-3982](1415 nt)
=-=-=-=-=-=-=-=-=-=-=-=-=-=-=-=-=-=-=-=-=-=-=-=-=-=-=-=-=-=-=-= Performing Scan: ame-miR-21 vs lcl|comp28960_c0_seq1:2569-3983
=-=-=-=-=-=-=-=-=-=-=-=-=-=-=-=-=-=-=-=-=-=-=-=-=-=-=-=-=-=-=-=


Forward: 	Score: 143.000000 	Q:3 to 22 	R:1332 to 1357 Align Len
(21) (71.43%) (80.95%)

Query: 	3' cagUUGUAGUC-AGA-CUAUUUCGau 5'
:|| |||| 	|| |:||||||
Ref: 		5' tcaGACTTCAGAACTGGGTAAAGCgt 3' Energy: 	-17.959999 kCal/Mol
rps6ka1 (Zebrafish) T-bulge

Read Sequence:ENSDARG00000033437_ENSDART00000044378_rps6ka1_ribosomal protein S6 kinase a, polypeptide 1 [Source:ZFIN;Acc:ZDB-GENE-060929-
516](1004 nt)
=-=-=-=-=-=-=-=-=-=-=-=-=-=-=-=-=-=-=-=-=-=-=-=-=-=-=-=-=-=-=-= Performing Scan: dre-miR-21-1-5p vs ENSDARG00000033437_ENSDART00000044378_rps6ka1_ribosomal
=-=-=-=-=-=-=-=-=-=-=-=-=-=-=-=-=-=-=-=-=-=-=-=-=-=-=-=-=-=-=-=

Forward: 	Score: 142.000000 	Q:2 to 22 	R:806 to 832 Align Len
(24) (58.33%) (70.83%)

Query: 	3' cggUUGUGGUCAGACU----AUUUCGAu 5'
||::: || 	||| 	||||||| Ref:	5' ctgAATGTGAG-ATGACATCTAAAGCTt 3'

Energy: 	-10.990000 kCal/Mol


rps6ka1 (Bichir) T-bulge

Read Sequence:lcl|comp48717_c0_seq3:2363-5682 comp48717_c0_seq3 len=5682 path=[63295906:0-260 63210071:261-4497 63271604:4498-
5681](3320 nt)
=-=-=-=-=-=-=-=-=-=-=-=-=-=-=-=-=-=-=-=-=-=-=-=-=-=-=-=-=-=-=-= Performing Scan: dre-miR-21-1-5p vs lcl|comp48717_c0_seq3:2363-5682
=-=-=-=-=-=-=-=-=-=-=-=-=-=-=-=-=-=-=-=-=-=-=-=-=-=-=-=-=-=-=-=

Forward: 	Score: 169.000000 	Q:2 to 23 	R:642 to 666 Align Len
(22) (72.73%) (81.82%)

Query: 	3' cgGUUGU-GGUCAGACUAUUUCGAu 5'
||||| :|| || 	:||||||| Ref:	5' ttCAACATTCATTCCTGTAAAGCTa 3'

Energy: 	-20.900000 kCal/Mol


rps6ka1 (Axolotl) T-bulge

Read Sequence:lcl|comp23386_c0_seq1:c738-1 comp23386_c0_seq1 len=3687 path=[29417206:0-2892 29499979:2893-3686](738 nt)
=-=-=-=-=-=-=-=-=-=-=-=-=-=-=-=-=-=-=-=-=-=-=-=-=-=-=-=-=-=-=-= Performing Scan: ame-miR-21 vs lcl|comp23386_c0_seq1:c738-1

=-=-=-=-=-=-=-=-=-=-=-=-=-=-=-=-=-=-=-=-=-=-=-=-=-=-=-=-=-=-=-=

Forward: 	Score: 142.000000 	Q:2 to 20 	R:183 to 209 Align Len
(21) (66.67%) (76.19%)

Query: 	3' caguuGUAGUCAGACU---AUUUCGAu 5'
||||| 	|||: 	|:||||| Ref:	5' actgcCATCACACTGGATTTGAAGCTg 3'

Energy: 	-17.780001 kCal/Mol

shc1 (Zebrafish)

Read Sequence:ENSDARG00000075437_ENSDART00000147609_shc1_SHC  (Src homology 2 domain containing) transforming protein 1 [Source:ZFIN;Acc:ZDB-GENE-030131-2538](2394 nt)
=-=-=-=-=-=-=-=-=-=-=-=-=-=-=-=-=-=-=-=-=-=-=-=-=-=-=-=-=-=-=-= Performing Scan: dre-miR-21-1-5p vs ENSDARG00000075437_ENSDART00000147609_shc1_SHC
=-=-=-=-=-=-=-=-=-=-=-=-=-=-=-=-=-=-=-=-=-=-=-=-=-=-=-=-=-=-=-=

Forward: 	Score: 145.000000 	Q:2 to 22 	R:878 to 900 Align Len
(20) (60.00%) (80.00%)

Query: 	3' cgGUUGUGGUCAGACUAUUCGAu 5'
|| 	: ::| |||:||||||
Ref: 		5' tgCATAGATGGGCTGGTAAGCTt 3' Energy: 	-18.620001 kCal/Mol


shc1 (Bichir) T-bulge

Read Sequence:lcl|comp48169_c0_seq5:c5404-1 comp48169_c0_seq5 len=7066 path=[61360692:0-6012 61444857:6013-6198 61447509:6199-6851
61457029:6852-7065](5404 nt)
=-=-=-=-=-=-=-=-=-=-=-=-=-=-=-=-=-=-=-=-=-=-=-=-=-=-=-=-=-=-=-= Performing Scan: dre-miR-21-1-5p vs lcl|comp48169_c0_seq5:c5404-1
=-=-=-=-=-=-=-=-=-=-=-=-=-=-=-=-=-=-=-=-=-=-=-=-=-=-=-=-=-=-=-=

Forward: 	Score: 142.000000 	Q:2 to 23 	R:1152 to 1175 Align Len
(21) (61.90%) (85.71%)

Query: 	3' cgGUUGUGGUCAGACUAUUUCGAu 5'
:|| :: ||||| ||:|||:|
Ref: 		5' ccTAAAGTGAGTCTCATGAAGTTg 3' Energy: 	-18.870001 kCal/Mol
shc1 (Axolotl) T-bulge

Read Sequence:lcl|comp41047_c0_seq1:c5409-1 comp41047_c0_seq1 len=7049 path=[112698591:0-744 112710372:745-815 112711360:816-1668
112724022:1669-1683 112724240:1684-1864 112727309:1865-1879
112727600:1880-2191 112732620:2192-2203 112732900:2204-2470
112737178:2471-2480 112737306:2481-2508 112737638:2509-2527

112738046:2528-2787 112742260:2788-2803 112742388:2804-3075
112746799:3076-3094 112747066:3095-3142 112747835:3143-3158
112748121:3159-4545 112770929:4546-4556 112771073:4557-4618
112771946:4619-4767 112774172:4768-6847 112808372:6848-6887
112808990:6888-7048](5409 nt)
=-=-=-=-=-=-=-=-=-=-=-=-=-=-=-=-=-=-=-=-=-=-=-=-=-=-=-=-=-=-=-= Performing Scan: ame-miR-21 vs lcl|comp41047_c0_seq1:c5409-1
=-=-=-=-=-=-=-=-=-=-=-=-=-=-=-=-=-=-=-=-=-=-=-=-=-=-=-=-=-=-=-=

Forward: 	Score: 142.000000 	Q:2 to 23 	R:1393 to 1416 Align Len
(21) (61.90%) (85.71%)

Query: 	3' caGUUGUAGUCAGACUAUUUCGAu 5'
||| :|:: ||| |||:||:|
Ref: 		5' tcCAAGGTTGATCTAATAGAGTTt 3' Energy: 	-15.340000 kCal/Mol
spry2 (Zebrafish) T-bulge

Read Sequence:ENSDARG00000078308_ENSDART00000052423_spry2_sprouty homolog 2 [Source:ZFIN;Acc:ZDB-GENE-030131-7038](863 nt)
=-=-=-=-=-=-=-=-=-=-=-=-=-=-=-=-=-=-=-=-=-=-=-=-=-=-=-=-=-=-=-= Performing Scan: dre-miR-21-1-5p vs ENSDARG00000078308_ENSDART00000052423_spry2_sprouty
=-=-=-=-=-=-=-=-=-=-=-=-=-=-=-=-=-=-=-=-=-=-=-=-=-=-=-=-=-=-=-=

Forward: 	Score: 141.000000 	Q:2 to 23 	R:300 to 324 Align Len
(22) (68.18%) (81.82%)

Query: 	3' cgGUUGUGGU-CAGACUAUUUCGAu 5'
||::| :| | ||||||||| | Ref:	5' ttCAGTAATATGACTGATAAAGGTg 3'

Energy: 	-17.400000 kCal/Mol


spry2 (Bichir) T-bulge

Read Sequence:lcl|comp23257_c0_seq1:c785-1 comp23257_c0_seq1 len=2183 path=[21275900:0-1784 21317840:1785-2182](785 nt)
=-=-=-=-=-=-=-=-=-=-=-=-=-=-=-=-=-=-=-=-=-=-=-=-=-=-=-=-=-=-=-= Performing Scan: dre-miR-21-1-5p vs lcl|comp23257_c0_seq1:c785-1
=-=-=-=-=-=-=-=-=-=-=-=-=-=-=-=-=-=-=-=-=-=-=-=-=-=-=-=-=-=-=-=

Forward: 	Score: 144.000000 	Q:2 to 22 	R:216 to 240 Align Len
(21) (57.14%) (71.43%)

Query: 	3' cggUUGUGGUCAGACU-AUUUCGAu 5'
|::| | | : 	| ||||||| Ref:	5' tcaAGTAACTGATAAACTAAAGCTg 3'

Energy: 	-12.570000 kCal/Mol


spry2 (Axolotl)


Read Sequence:lcl|comp36045_c0_seq1:c799-1 comp36045_c0_seq1 len=2547 path=[70674239:0-1331 70699821:1332-1457 70702160:1458-2127
70714646:2128-2546](799 nt)
=-=-=-=-=-=-=-=-=-=-=-=-=-=-=-=-=-=-=-=-=-=-=-=-=-=-=-=-=-=-=-= Performing Scan: ame-miR-21 vs lcl|comp36045_c0_seq1:c799-1
=-=-=-=-=-=-=-=-=-=-=-=-=-=-=-=-=-=-=-=-=-=-=-=-=-=-=-=-=-=-=-=

Forward: 	Score: 143.000000 	Q:2 to 16 	R:221 to 243 Align Len
(14) (71.43%) (71.43%)

Query: 	3' caguuguaGUCAGACUAUUCGAu 5'
|| 	| 	||||||| Ref:	5' actctagcCAACCACATAAGCTa 3'

Energy: 	-10.110000 kCal/Mol

tegt (Zebrafish) T-bulge

Read Sequence:ENSDARG00000077934_ENSDART00000108703_tegt_testis enhanced gene transcript (BAX inhibitor 1) [Source:ZFIN;Acc:ZDB-GENE-
030826-10](504 nt)
=-=-=-=-=-=-=-=-=-=-=-=-=-=-=-=-=-=-=-=-=-=-=-=-=-=-=-=-=-=-=-= Performing Scan: dre-miR-21-1-5p vs ENSDARG00000077934_ENSDART00000108703_tegt_testis
=-=-=-=-=-=-=-=-=-=-=-=-=-=-=-=-=-=-=-=-=-=-=-=-=-=-=-=-=-=-=-=

Forward: 	Score: 141.000000 	Q:2 to 23 	R:386 to 410 Align Len
(22) (63.64%) (86.36%)

Query: 	3' cgGUUGUGGUCAGA-CUAUUUCGAu 5'
:|||:|||||:| 	||:||:| Ref:	5' gtTAACGCCAGTTTATTTAGAGTTc 3'

Energy: 	-24.809999 kCal/Mol


tegt (Bichir) T-bulge

Read Sequence:lcl|comp16220_c0_seq1:879-2448 comp16220_c0_seq1 len=2448 path=[14877221:0-1285 14895438:1286-2447](1570 nt)
=-=-=-=-=-=-=-=-=-=-=-=-=-=-=-=-=-=-=-=-=-=-=-=-=-=-=-=-=-=-=-= Performing Scan: dre-miR-21-1-5p vs lcl|comp16220_c0_seq1:879-2448
=-=-=-=-=-=-=-=-=-=-=-=-=-=-=-=-=-=-=-=-=-=-=-=-=-=-=-=-=-=-=-=

Forward: 	Score: 146.000000 	Q:2 to 22 	R:1337 to 1358 Align Len
(20) (60.00%) (75.00%)

Query: 	3' cggUUGUGGUCAGACUAUUUCGAu 5'
||:|: : 	|| 	||||||| Ref:	5' ttaAATATGGCACT--TAAAGCTg 3'

Energy: 	-10.910000 kCal/Mol

tegt (Axolotl)

Read Sequence:lcl|comp26367_c0_seq1:c1736-1 comp26367_c0_seq1 len=2693 path=[35746354:0-231 35751127:232-1899 35786283:1900-1906
35786461:1907-2692](1736 nt)
=-=-=-=-=-=-=-=-=-=-=-=-=-=-=-=-=-=-=-=-=-=-=-=-=-=-=-=-=-=-=-= Performing Scan: ame-miR-21 vs lcl|comp26367_c0_seq1:c1736-1
=-=-=-=-=-=-=-=-=-=-=-=-=-=-=-=-=-=-=-=-=-=-=-=-=-=-=-=-=-=-=-=

Forward: 	Score: 150.000000 	Q:2 to 11 	R:998 to 1020 Align Len
(9) (100.00%) (100.00%)

Query: 	3' caguuguagucagACUAUUCGAu 5'
||||||||| Ref:	5' tctgctttctcaaTGATAAGCTg 3'

Energy: 	-15.060000 kCal/Mol


dnajc18 (Zebrafish) T-bulge

Read Sequence:ENSDARG00000056005_ENSDART00000101054_dnajc18_DnaJ (Hsp40) homolog, subfamily C, member 18 [Source:ZFIN;Acc:ZDB-GENE-
030131-8019](1078 nt)
=-=-=-=-=-=-=-=-=-=-=-=-=-=-=-=-=-=-=-=-=-=-=-=-=-=-=-=-=-=-=-= Performing Scan: dre-miR-21-1-5p vs ENSDARG00000056005_ENSDART00000101054_dnajc18_DnaJ
=-=-=-=-=-=-=-=-=-=-=-=-=-=-=-=-=-=-=-=-=-=-=-=-=-=-=-=-=-=-=-=

Forward: 	Score: 146.000000 	Q:2 to 17 	R:316 to 341 Align Len
(17) (76.47%) (76.47%)

Query: 	3' cgguugugGUC-AGACU-AUUUCGAu 5'
||| | 	|| ||||||| Ref:	5' caggctggCAGATGGGATTAAAGCTa 3'

Energy: 	-16.110001 kCal/Mol


dnajc18 (Bichir)

Read Sequence:lcl|comp43410_c0_seq1:c612-1 comp43410_c0_seq1 len=2021 path=[49365481:0-2020](612 nt)
=-=-=-=-=-=-=-=-=-=-=-=-=-=-=-=-=-=-=-=-=-=-=-=-=-=-=-=-=-=-=-=
Performing Scan: dre-miR-21-1-5p vs lcl|comp43410_c0_seq1:c612-1
=-=-=-=-=-=-=-=-=-=-=-=-=-=-=-=-=-=-=-=-=-=-=-=-=-=-=-=-=-=-=-=

Forward: 	Score: 142.000000 	Q:2 to 20 	R:304 to 328 Align Len
(20) (80.00%) (80.00%)

Query: 	3' cgguUGUGG--UCAGACUAUUCGAu 5'
||||| 	| ||||||||| | Ref:	5' atttACACCTTAATCTGATAAGGTg 3'

Energy: 	-21.389999 kCal/Mol


dnajc18 (Axolotl)

Read Sequence:lcl|comp39278_c0_seq1:c1977-1 comp39278_c0_seq1 len=2309 path=[92539126:0-20 92539769:21-334 92547913:335-445 92550275:446-474
92550931:475-497 92551717:498-606 92554074:607-1262 92571067:1263-1608
92579615:1609-2308](1977 nt)
=-=-=-=-=-=-=-=-=-=-=-=-=-=-=-=-=-=-=-=-=-=-=-=-=-=-=-=-=-=-=-= Performing Scan: ame-miR-21 vs lcl|comp39278_c0_seq1:c1977-1
=-=-=-=-=-=-=-=-=-=-=-=-=-=-=-=-=-=-=-=-=-=-=-=-=-=-=-=-=-=-=-=

Forward: 	Score: 143.000000 	Q:2 to 22 	R:1127 to 1148 Align Len
(20) (70.00%) (90.00%)

Query: 	3' caGUUGUAGUCAGACUAUUCGAu 5'
||:|||: | |||:|:||||
Ref: 		5' gcCAGCATTTG-CTGGTGAGCTg 3' Energy: 	-24.950001 kCal/Mol
efna5a (Zebrafish)

Read Sequence:ENSDARG00000089790_ENSDART00000126005_efna5a_ephrin  A5a
[Source:ZFIN;Acc:ZDB-GENE-001128-1](3969 nt)
=-=-=-=-=-=-=-=-=-=-=-=-=-=-=-=-=-=-=-=-=-=-=-=-=-=-=-=-=-=-=-= Performing Scan: dre-miR-21-1-5p vs ENSDARG00000089790_ENSDART00000126005_efna5a_ephrin
=-=-=-=-=-=-=-=-=-=-=-=-=-=-=-=-=-=-=-=-=-=-=-=-=-=-=-=-=-=-=-=

Forward: 	Score: 147.000000 	Q:2 to 22 	R:2912 to 2933 Align Len
(20) (65.00%) (85.00%)

Query: 	3' cgGUUGUGGUCAGACUAUUCGAu 5'
:||||: | |:| |||:|||
Ref: 		5' ccTAACATGA-TTTAATAGGCTc 3' Energy: 	-15.080000 kCal/Mol
efna5 (Bichir) T-bulge

Read Sequence:lcl|comp56815_c0_seq3:c1476-1 comp56815_c0_seq3 len=2268 path=[102957084:0-245 102961493:246-357 102963746:358-919
102973453:920-1016 102975677:1017-1990 102992743:1991-2267](1476 nt)
=-=-=-=-=-=-=-=-=-=-=-=-=-=-=-=-=-=-=-=-=-=-=-=-=-=-=-=-=-=-=-= Performing Scan: dre-miR-21-1-5p vs lcl|comp56815_c0_seq3:c1476-1
=-=-=-=-=-=-=-=-=-=-=-=-=-=-=-=-=-=-=-=-=-=-=-=-=-=-=-=-=-=-=-=

Forward: 	Score: 148.000000 	Q:2 to 23 	R:74 to 99 Align Len (23) (69.57%) (78.26%)

Query: 	3' cgGUUGUGGUC-AGAC-UAUUUCGAu 5'
||| :| || | || ||||||:|
Ref: 	5' taCAAGGCAAGCTGTGAATAAAGTTa 3'

Energy: 	-15.930000 kCal/Mol


efna5 (Axolotl)

Read Sequence:lcl|comp39265_c0_seq1:c3038-1 comp39265_c0_seq1 len=4415 path=[92481651:0-824 92499022:825-1420 92510971:1421-1591
92514583:1592-1603 92514824:1604-2901 92542103:2902-3158 92548049:3159-
3779 92591516:3780-4134 92567806:4135-4414](3038 nt)
=-=-=-=-=-=-=-=-=-=-=-=-=-=-=-=-=-=-=-=-=-=-=-=-=-=-=-=-=-=-=-= Performing Scan: ame-miR-21 vs lcl|comp39265_c0_seq1:c3038-1
=-=-=-=-=-=-=-=-=-=-=-=-=-=-=-=-=-=-=-=-=-=-=-=-=-=-=-=-=-=-=-=

Forward: 	Score: 173.000000 	Q:2 to 22 	R:2008 to 2030 Align Len
(20) (75.00%) (85.00%)

Query: 	3' caGUUGUAGUCAGACUAUUCGAu 5'
|| :||||| : |||||||| Ref:	5' gcCATTATCAGATCGATAAGCTg 3'

Energy: 	-23.080000 kCal/Mol


hsd3b7 (Zebrafish)

Read Sequence:ENSDARG00000036966_ENSDART00000053684_hsd3b7_hydroxy- delta-5-steroid dehydrogenase, 3 beta- and steroid delta-isomerase [Source:ZFIN;Acc:ZDB-GENE-030131-5673](520 nt)
=-=-=-=-=-=-=-=-=-=-=-=-=-=-=-=-=-=-=-=-=-=-=-=-=-=-=-=-=-=-=-= Performing Scan: dre-miR-21-1-5p vs ENSDARG00000036966_ENSDART00000053684_hsd3b7_hydroxy-delta-5-steroid
=-=-=-=-=-=-=-=-=-=-=-=-=-=-=-=-=-=-=-=-=-=-=-=-=-=-=-=-=-=-=-=

Forward: 	Score: 159.000000 	Q:2 to 22 	R:252 to 273 Align Len
(20) (70.00%) (80.00%)

Query: 	3' cgGUUGUGGUCAGACUAUUCGAu 5'
:|:| 	||||| ||||||| Ref:	5' gtTAGCTAAAGTCT-ATAAGCTg 3'

Energy: 	-21.500000 kCal/Mol

hsd3b7 (Bichir)

Read Sequence:lcl|comp56957_c0_seq2:c2107-1 comp56957_c0_seq2 len=3620 path=[104068072:0-921 104080794:922-971 104204699:972-974
104204703:975-978 104081370:979-1136 104204508:1137-1140
104204512:1141-1160 104204532:1161-1165 104204537:1166-1177
104098971:1178-2235 104204598:2236-2360 104115035:2361-3270
104204734:3271-3521 104192182:3522-3619](2107 nt)
=-=-=-=-=-=-=-=-=-=-=-=-=-=-=-=-=-=-=-=-=-=-=-=-=-=-=-=-=-=-=-= Performing Scan: dre-miR-21-1-5p vs lcl|comp56957_c0_seq2:c2107-1
=-=-=-=-=-=-=-=-=-=-=-=-=-=-=-=-=-=-=-=-=-=-=-=-=-=-=-=-=-=-=-=

Forward: 	Score: 151.000000 	Q:2 to 16 	R:1283 to 1305 Align Len
(14) (78.57%) (78.57%)

Query: 	3' cgguugugGUCAGACUAUUCGAu 5'
||| 	| ||||||| Ref:	5' tggccgtaCAGAATTATAAGCTc 3'

Energy: 	-17.200001 kCal/Mol


hsd3b7 (Axolotl) T-bulge

Read Sequence:lcl|comp15354_c0_seq1:c1571-1 comp15354_c0_seq1 len=2791 path=[17345230:0-1707 17382241:1708-2790](1571 nt)
=-=-=-=-=-=-=-=-=-=-=-=-=-=-=-=-=-=-=-=-=-=-=-=-=-=-=-=-=-=-=-= Performing Scan: ame-miR-21 vs lcl|comp15354_c0_seq1:c1571-1
=-=-=-=-=-=-=-=-=-=-=-=-=-=-=-=-=-=-=-=-=-=-=-=-=-=-=-=-=-=-=-=

Forward: 	Score: 145.000000 	Q:2 to 22 	R:1286 to 1311 Align Len
(23) (69.57%) (82.61%)

Query: 	3' cagUUGUAG-UCA--GACUAUUUCGAu 5'
|::||| ||| 	|| ||||||:| Ref:	5' agaAGTATCTAGTAGCT-ATAAAGTTg 3'

Energy: 	-14.900000 kCal/Mol


mocs1 (Zebrafish) T-bulge

Read Sequence:ENSDARG00000078479_ENSDART00000156405_mocs1_molybdenum cofactor synthesis 1 [Source:ZFIN;Acc:ZDB-GENE-130215-1](1136 nt)
=-=-=-=-=-=-=-=-=-=-=-=-=-=-=-=-=-=-=-=-=-=-=-=-=-=-=-=-=-=-=-=
Performing Scan: dre-miR-21-1-5p vs
ENSDARG00000078479_ENSDART00000156405_mocs1_molybdenum
=-=-=-=-=-=-=-=-=-=-=-=-=-=-=-=-=-=-=-=-=-=-=-=-=-=-=-=-=-=-=-=

Forward: 	Score: 142.000000 	Q:2 to 23 	R:946 to 969 Align Len
(21) (57.14%) (76.19%)

Query: 	3' cgGUUGUGGUCAGACUAUUUCGAu 5'
:| ||:|| | 	:|||||:|
Ref: 		5' atTATCATCACTGATGTAAAGTTa 3' Energy: 	-16.549999 kCal/Mol
mocs1 (Bichir) T-bulge

Read Sequence:lcl|comp57751_c0_seq2:3603-4026 comp57751_c0_seq2
len=4026 path=[110940030:0-2715 110978125:2716-3082 110982880:3083-3411
110987383:3412-3438 110987743:3439-3502 110988607:3503-3571
110989503:3572-3593 110989778:3594-3995 111068359:3996-4025](424 nt)
=-=-=-=-=-=-=-=-=-=-=-=-=-=-=-=-=-=-=-=-=-=-=-=-=-=-=-=-=-=-=-= Performing Scan: dre-miR-21-1-5p vs lcl|comp57751_c0_seq2:3603-4026
=-=-=-=-=-=-=-=-=-=-=-=-=-=-=-=-=-=-=-=-=-=-=-=-=-=-=-=-=-=-=-=


Forward: 	Score: 142.000000 	Q:3 to 22 	R:221 to 247 Align Len
(22) (72.73%) (81.82%)

Query: 	3' cggUUGUGGU-CA-GA-CUAUUUCGau 5'
||:: || || || ||||||||
Ref: 		5' tcaAATGGCATGTACTCGATAAAGCgc 3' Energy: 	-14.210000 kCal/Mol


mocs1 (Axolotl) T-bulge

Read Sequence:lcl|comp15468_c0_seq2:1558-3321 comp15468_c0_seq2 len=3321 path=[17737889:0-1086 17756788:1087-2645 17784643:2646-
3320](1764 nt)
=-=-=-=-=-=-=-=-=-=-=-=-=-=-=-=-=-=-=-=-=-=-=-=-=-=-=-=-=-=-=-= Performing Scan: ame-miR-21 vs lcl|comp15468_c0_seq2:1558-3321
=-=-=-=-=-=-=-=-=-=-=-=-=-=-=-=-=-=-=-=-=-=-=-=-=-=-=-=-=-=-=-=

Forward: 	Score: 144.000000 	Q:3 to 23 	R:441 to 463 Align Len
(20) (65.00%) (85.00%)

Query: 	3' caGUUGUAGUCAGACUAUUUCGau 5'
::||| ::|| || ||||||
Ref: 		5' aaTGACACTGGT-TGTTAAAGCac 3' Energy: 	-11.760000 kCal/Mol


pm20d1.2 (Zebrafish)

Read Sequence:ENSDARG00000062096_ENSDART00000104592_pm20d1.2_peptidase
M20 domain containing 1, tandem duplicate 2 [Source:ZFIN;Acc:ZDB-GENE-
061013-637](2308 nt)
=-=-=-=-=-=-=-=-=-=-=-=-=-=-=-=-=-=-=-=-=-=-=-=-=-=-=-=-=-=-=-= Performing Scan: dre-miR-21-1-5p vs ENSDARG00000062096_ENSDART00000104592_pm20d1.2_peptidase
=-=-=-=-=-=-=-=-=-=-=-=-=-=-=-=-=-=-=-=-=-=-=-=-=-=-=-=-=-=-=-=

Forward: 	Score: 143.000000 	Q:2 to 21 	R:1644 to 1669 Align Len
(22) (59.09%) (68.18%)

Query: 	3' cggUUGUGGUCAGAC---UAUUCGAu 5'
||:: | | 	|| 	||||||| Ref:	5' ctgAATGACTGAGTGAACATAAGCTa 3'

Energy: 	-11.480000 kCal/Mol

pm20d1 (Bichir) T-bulge

Read Sequence:lcl|comp58155_c0_seq18:c1554-1 comp58155_c0_seq18 len=3312 path=[115843970:0-21 115784311:22-36 115856478:37-37
115856479:38-38 115856480:39-39 115273807:40-42 115273840:43-45
115856483:46-66 115856504:67-68 115856506:69-77 115856515:78-78

115609721:79-300 115612849:301-1536 115630330:1537-2490 115644095:2491-
3019 115650961:3020-3311](1554 nt)
=-=-=-=-=-=-=-=-=-=-=-=-=-=-=-=-=-=-=-=-=-=-=-=-=-=-=-=-=-=-=-= Performing Scan: dre-miR-21-1-5p vs lcl|comp58155_c0_seq18:c1554-1
=-=-=-=-=-=-=-=-=-=-=-=-=-=-=-=-=-=-=-=-=-=-=-=-=-=-=-=-=-=-=-=

Forward: 	Score: 148.000000 	Q:2 to 23 	R:460 to 482 Align Len
(21) (76.19%) (85.71%)

Query: 	3' cgGUUGUGGUCAGACUAUUUCGAu 5'
|||||:|||| | :|||| || Ref:	5' taCAACATCAGT-TAGTAAAACTt 3'

Energy: 	-19.000000 kCal/Mol


pm20d1 (Axolotl) T-bulge

Read Sequence:lcl|comp15468_c0_seq2:1558-3321 comp15468_c0_seq2 len=3321 path=[17737889:0-1086 17756788:1087-2645 17784643:2646-
3320](1764 nt)
=-=-=-=-=-=-=-=-=-=-=-=-=-=-=-=-=-=-=-=-=-=-=-=-=-=-=-=-=-=-=-= Performing Scan: ame-miR-21 vs lcl|comp15468_c0_seq2:1558-3321
=-=-=-=-=-=-=-=-=-=-=-=-=-=-=-=-=-=-=-=-=-=-=-=-=-=-=-=-=-=-=-=

Forward: 	Score: 144.000000 	Q:3 to 23 	R:441 to 463 Align Len
(20) (65.00%) (85.00%)

Query: 	3' caGUUGUAGUCAGACUAUUUCGau 5'
::||| ::|| || ||||||
Ref: 		5' aaTGACACTGGT-TGTTAAAGCac 3' Energy: 	-11.760000 kCal/Mol
sort1a (Zebrafish)

Read Sequence:ENSDARG00000029402_ENSDART00000020187_sort1a_sortilin 1a
[Source:ZFIN;Acc:ZDB-GENE-040426-2329](704 nt)
=-=-=-=-=-=-=-=-=-=-=-=-=-=-=-=-=-=-=-=-=-=-=-=-=-=-=-=-=-=-=-= Performing Scan: dre-miR-21-1-5p vs ENSDARG00000029402_ENSDART00000020187_sort1a_sortilin
=-=-=-=-=-=-=-=-=-=-=-=-=-=-=-=-=-=-=-=-=-=-=-=-=-=-=-=-=-=-=-=

Forward: 	Score: 145.000000 	Q:2 to 22 	R:563 to 586 Align Len
(20) (75.00%) (80.00%)

Query: 	3' cggUUGUGGUCAGACUAUUUCGAu 5'
|| |: ||||| |||||| | Ref:	5' aaaAAAATGAGTCTCATAAAGATt 3'

Energy: 	-13.390000 kCal/Mol

sort1  (Bichir)

Read Sequence:lcl|comp57712_c0_seq1:373-2306 comp57712_c0_seq1 len=2306 path=[110570619:0-256 110576999:257-412 110579095:413-586

110581555:587-795 110585003:796-1087 110589372:1088-1113
110589652:1114-1180 110590450:1181-1504 110595176:1505-2305](1934 nt)
=-=-=-=-=-=-=-=-=-=-=-=-=-=-=-=-=-=-=-=-=-=-=-=-=-=-=-=-=-=-=-= Performing Scan: dre-miR-21-1-5p vs lcl|comp57712_c0_seq1:373-2306
=-=-=-=-=-=-=-=-=-=-=-=-=-=-=-=-=-=-=-=-=-=-=-=-=-=-=-=-=-=-=-=

Forward: 	Score: 147.000000 	Q:2 to 21 	R:1267 to 1290 Align Len
(20) (60.00%) (75.00%)

Query: 	3' cggUUGUGGUCAGAC-UAUUCGAu 5'
|: |:| 	:|| |||||||
Ref: 		5' atgAGGATCTCCTTGAATAAGCTg 3' Energy: 	-15.050000 kCal/Mol


sort1  (Axolotl)

Read Sequence:lcl|comp37104_c0_seq2:2410-5194 comp37104_c0_seq2 len=5194 path=[77012802:0-5049 77096783:5050-5073 77152511:5074-5104
77214924:5105-5129 77153395:5130-5193](2785 nt)
=-=-=-=-=-=-=-=-=-=-=-=-=-=-=-=-=-=-=-=-=-=-=-=-=-=-=-=-=-=-=-= Performing Scan: ame-miR-21 vs lcl|comp37104_c0_seq2:2410-5194
=-=-=-=-=-=-=-=-=-=-=-=-=-=-=-=-=-=-=-=-=-=-=-=-=-=-=-=-=-=-=-=

Forward: 	Score: 156.000000 	Q:2 to 13 	R:1092 to 1114 Align Len
(11) (90.91%) (100.00%)

Query: 	3' caguuguagucAGACUAUUCGAu 5'
|:||||||||| Ref:	5' ggggggggtgaTTTGATAAGCTa 3'

Energy: 	-16.780001 kCal/Mol
