# Supplementary material for: A Conserved MicroRNA Regulatory Circuit Is Differentially Controlled during Limb/Appendage Regeneration
Source: PLoS One. 2016 Jun 29;11(6):e0157106. doi: 10.1371/journal.pone.0157106 (PMC4927183; doi:10.1371/journal.pone.0157106)
Supplement: S3 File — Sequences of mature miRNAs for the common up- and downregulated miRNAs along with sequences that allow a nucleation bulge at positions 5–6 [76] for zebrafish, bichir and axolotl. (DOC) [file pone.0157106.s004.doc]

S3 File: Mature miRNA sequences  used for miRNA target prediction.

Zebrafish mature miRNA sequences.

>dre-miR-21-1-5p grp3295-4000898
TAGCTTATCAGACTGGTGTTGGC
>dre-miR-181c-5p grp2236-3214
CACATTCATTGCTGTCGGTGGGTT
>dre-miR-181b-1-5p grp11650-57168
AACATTCATTGCTGTCGGTGGGT
>dre-miR-31-5p grp10983-3372
TGGCAAGATGTTGGCATAGCTG
>dre-miR-7b-5p grp8085-202
TGGAAGACTTGTGATTTTGTTGTT
>dre-miR-2184-1-5p grp10995-43606
AACAGTAAGAGTTTATGTGCTG
>dre-miR-204-2-5p grp6554-150213
TTCCCTTTGTCATCCTATGCCT
>dre-miR-338-1-3p grp8564-6698
TCCAGCATCAGTGATTTTGTT
>dre-miR-133a-1-3p grp7726-94
TTTGGTCCCCTTCAACCAGCTGT
>dre-miR-24-1-3p grp12832-2176
TGGCTCAGTTCAGCAGGAAC

Zebrafish mature miRNA sequences  with nucleation bulge at positions 5-6.

>dre-miR-21-1-5p grp3295-4000898
TAGCTTTATCAGACTGGTGTTGGC
>dre-miR-181c-5p grp2236-3214
CACATTTCATTGCTGTCGGTGGGTT
>dre-miR-181b-1-5p grp11650-57168
AACATTTCATTGCTGTCGGTGGGT
>dre-miR-31-5p grp10983-3372
TGGCAAAGATGTTGGCATAGCTG
>dre-miR-7b-5p grp8085-202
TGGAAAGACTTGTGATTTTGTTGTT
>dre-miR-2184-1-5p grp10995-43606
AACAGGTAAGAGTTTATGTGCTG
>dre-miR-204-2-5p grp6554-150213
TTCCCCTTTGTCATCCTATGCCT
>dre-miR-338-1-3p grp8564-6698
TCCAGGCATCAGTGATTTTGTT
>dre-miR-133a-1-3p grp7726-94
TTTGGGTCCCCTTCAACCAGCTGT
>dre-miR-24-1-3p grp12832-2176

TGGCTTCAGTTCAGCAGGAAC

Bichir  mature miRNA sequences.

>pse-miR-21-1-5p grp27078-848326
TAGCTTATCAGACTGGTGTTGGC
>pse-miR-181c-5p grp21835-2551
AACATTCATTGCTGTCGGTGGGTT
>pse-miR-181b-1-5p grp27615-1644
AACATTCATTGCTGTCGGTGGGT
>pse-miR-31-5p grp24882-954
TGCTATGCCTACATACTGCCATC
>pse-miR-7b-5p grp15169-548
TGGAAGACTAGTGATTTTGTTGTT
>pse-miR-2184-1-5p grp15092-1913
AACAGTAAGAGTTTATGTGTTG
>pse-miR-204-2-5p grp1308-555
TTCCCTTTGTCATCCTATGCCT
>pse-miR-338-1-3p grp24338-321
TCCAGCATCAGTGATTTTGTTG
>pse-miR-133a-1-3p grp13373-5941
TTGGTCCCCTTCAACCAGCTGT
>pse-miR-24-1-3p grp9625-8875
TGGCTCAGTTCAGCAGGAACAGT

Bichir  mature miRNA sequences  with nucleation bulge at positions 5-6.

>pse-miR-21-1-5p grp27078-848326
TAGCTTTATCAGACTGGTGTTGGC
>pse-miR-181c-5p grp21835-2551
AACATTTCATTGCTGTCGGTGGGTT
>pse-miR-181b-1-5p grp27615-1644
AACATTTCATTGCTGTCGGTGGGT
>pse-miR-31-5p grp24882-954
TGCTAATGCCTACATACTGCCATC
>pse-miR-7b-5p grp15169-548
TGGAAAGACTAGTGATTTTGTTGTT
>pse-miR-2184-1-5p grp15092-1913
AACAGGTAAGAGTTTATGTGTTG
>pse-miR-204-2-5p grp1308-555
TTCCCCTTTGTCATCCTATGCCT
>pse-miR-338-1-3p grp24338-321
TCCAGGCATCAGTGATTTTGTTG
>pse-miR-133a-1-3p grp13373-5941
TTGGTTCCCCTTCAACCAGCTGT
>pse-miR-24-1-3p grp9625-8875
TGGCTTCAGTTCAGCAGGAACAGT


Axolotl mature miRNA sequences.

>ame-miR-21-1-5p grp6404-240235
TAGCTTATCAGACTGATGTTGAC
>ame-miR-181c-5p grp7734-669
AACATTCATTGCTGTCGGTGGGTT
>ame-miR-181b-1-5p grp19391-1327
AACATTCATTGCTGTCGGTGG
>ame-miR-31-5p grp5637-99
AGGCAAGATGTTGGCATAGCTGA
>ame-miR-7b-5p grp5349-125
TGGAAGACTAGTGATTTTGTTGTT
>ame-miR-2184-1-5p grp1566-17
AACAGTAAGAGTTAATGTGCTG
>ame-miR-204-2-5p grp434-265
TTCCCTTTGTCATCCTATGCCT
>ame-miR-338-1-3p grp9403-72
TCCAGCATCAGTGATTTTGTTGA
>ame-miR-133a-1-3p grp14422-10074
TTGGTCCCCTTCAACCAGCTGT
>ame-miR-24-1-3p grp12482-2925
TGGCTCAGTTCAGCAGGAACAG

Axolotl mature miRNA sequences  with nucleation bulge at positions 5-6.

>ame-miR-21-1-5p grp6404-240235
TAGCTTTATCAGACTGATGTTGAC
>ame-miR-181c-5p grp7734-669
AACATTTCATTGCTGTCGGTGGGTT
>ame-miR-181b-1-5p grp19391-1327
AACATTTCATTGCTGTCGGTGG
>ame-miR-31-5p grp5637-99
AGGCAAAGATGTTGGCATAGCTGA
>ame-miR-7b-5p grp5349-125
TGGAAAGACTAGTGATTTTGTTGTT
>ame-miR-2184-1-5p grp1566-17
AACAGGTAAGAGTTAATGTGCTG
>ame-miR-204-2-5p grp434-265
TTCCCCTTTGTCATCCTATGCCT
>ame-miR-338-1-3p grp9403-72
TCCAGGCATCAGTGATTTTGTTGA
>ame-miR-133a-1-3p grp14422-10074
TTGGTTCCCCTTCAACCAGCTGT
>ame-miR-24-1-3p grp12482-2925
TGGCTTCAGTTCAGCAGGAACAG
